# N-Acetylcysteine protects the developing brain in neonatal sepsis-like inflammation via a redox–neurovascular pathway

**DOI:** 10.1186/s12974-026-03942-9

**Published:** 2026-06-30

**Authors:** Ping Cheng, Shan Zhang, Xiaoli Zhang, Wenhua Li, Mengmeng Du, Mingchao Li, Zengyuan Yu, Jinjin Zhu, Guixiang Zeng, Yiran Xu, Huiqing Sun, Wenqing Kang, Xiaoyang Wang, Changlian Zhu

**Affiliations:** 1 Henan Key Laboratory of Child Brain Injury and Henan International Joint Laboratory for Child Brain Injury, Henan Pediatric Clinical Research Center, Institute of Neuroscience, Third Affiliated Hospital of Zhengzhou University, Zhengzhou, 450052 China; 2 Department of Neonatology, Children’s Hospital Affiliated to Zhengzhou University, Henan Children’s Hospital, Zhengzhou Children’s Hospital, Zhengzhou, 450018 China; 3 Center for Brain Repair and Rehabilitation, Institute of Neuroscience and Physiology, University of Gothenburg, Goteborg, 40530 Sweden; 4 Department of Neonatology, Nanning Maternity and Child Health Hospital, Nanning, 530011 China; 5 Centre of Perinatal Medicine and Health, Institute of Clinical Science, University of Gothenburg, Goteborg, 40530 Sweden

**Keywords:** N-acetylcysteine, Neonatal sepsis, Blood-brain barrier, White matter injury

## Abstract

**Background:**

Neonatal sepsis can disrupt brain development through oxidative stress, blood-brain barrier (BBB) dysfunction, peripheral leukocyte infiltration, and white matter injury. N-acetylcysteine (NAC), a glutathione precursor with antioxidant and immunomodulatory properties, is a promising neuroprotective candidate, but its effects in neonatal sepsis-like brain injury remain incompletely defined. The purpose of this study was to investigate whether early NAC pretreatment, followed by continued treatment, was associated with protection in a neonatal LPS model and examine the principal mechanisms associated with its effects.

**Methods:**

In this study, neonatal C57BL/6J mice received lipopolysaccharide (LPS; 3 mg/kg, subcutaneously) on postnatal day (PND) 3 to model sepsis-like injury. NAC was administered intraperitoneally 2 h before LPS (200 mg/kg), followed by daily treatment (100 mg/kg/day) through PND14. Survival was monitored to PND21. Acute outcomes at PND4 included oxidative stress, glial/inflammatory markers, MPO-positive cell accumulation, apoptosis, and BBB-related injury. Longer-term outcomes included myelination, dentate gyrus proliferation, and behavior.

**Results:**

NAC improved survival after neonatal LPS exposure, with exploratory sex-stratified analyses suggesting greater benefit in males. NAC partially restored cortical glutathione levels and reduced lipid peroxidation, indicating improved redox balance. Although it did not significantly alter galectin-3, GFAP, or NLRP3 at the time point examined, NAC reduced cortical MPO-positive cell burden and attenuated markers associated with neurovascular injury and BBB-associated pathology, including matrix metallopeptidase-9 expression and albumin extravasation. NAC also decreased apoptosis in selected brain regions and partly improved white matter-related outcomes, including oligodendrocyte precursor cell abundance, myelination, and early sensorimotor performance. However, long-term behavioral performance in the open field and novel object recognition tests was not significantly improved at PND60.

**Conclusions:**

Overall, early NAC pretreatment followed by continued treatment mitigated neonatal LPS-induced sepsis-like brain injury and improved survival, with protection associated with restoration of redox homeostasis and reduction of MPO-positive leukocyte and markers associated with neurovascular injury and BBB-associated pathology rather than broad suppression of measured inflammatory mediators. These findings are consistent with a potential role for a redox-neurovascular-leukocyte pathway in NAC-associated neuroprotection and support further evaluation of NAC in prevention-oriented experimental paradigms relevant to high-risk preterm populations.

**Supplementary Information:**

The online version contains supplementary material available at 10.1186/s12974-026-03942-9.

## Introduction

Approximately 13.4 million infants are born preterm worldwide each year, and these infants constitute a particularly vulnerable population at increased risk of infection [1]. Neonatal sepsis is a major cause of death and long-term neurodevelopmental disability, particularly in preterm infants [2–4]. In addition to acute systemic instability, early-life infection can disrupt critical stages of brain maturation and increase the risk of white matter injury, motor impairment, and later cognitive deficits [5, 6]. The developing brain is especially vulnerable during the preterm-equivalent period, when pre-oligodendrocytes predominate and are highly sensitive to inflammatory and oxidative injury [7, 8]. Although microglial activation and cytokine signaling are established contributors to perinatal brain injury, oxidative stress, blood-brain barrier (BBB) dysfunction, and peripheral immune cell recruitment are increasingly recognized as key drivers of inflammation-associated damage in the immature brain [8–10]. Therefore, there remains a critical need to identify neuroprotective strategies capable of reducing infection-associated brain injury in vulnerable preterm infants.

Systemic lipopolysaccharide (LPS) exposure is a widely used model of neonatal sepsis-like inflammation because it reproduces several features relevant to infection-associated brain injury, including BBB disruption, glial and vascular activation, and impaired white matter development [9, 11–13]. In neonatal rodents, exposure during the first postnatal days is particularly informative because this developmental stage broadly corresponds to the vulnerable human preterm brain [14–16]. This model therefore provides an opportunity to investigate how systemic inflammation affects redox homeostasis, neurovascular integrity, immune cell trafficking, and region-specific injury in the developing brain.

N-acetylcysteine (NAC) is a glutathione precursor with antioxidant and immunomodulatory properties and documented central nervous system bioavailability [17–20]. Because the immature brain has limited antioxidant defenses, NAC is an attractive candidate for neonatal neuroprotection, particularly in conditions where oxidative stress is prominent [8, 21–25]. Previous studies have shown that NAC can attenuate inflammation-sensitized brain injury, preserve oligodendrocyte lineage cells, and improve myelination in perinatal models [26–35]. NAC has also been reported to inhibit neutrophil transmigration into the developing brain, raising the possibility that its protective effects involve not only redox regulation but also modulation of brain-immune interfaces [36]. However, its role in postnatal neonatal sepsis-like brain injury remains incompletely understood, particularly with respect to neutrophil-associated injury, BBB dysfunction, regional vulnerability, and the relationship between early mechanistic effects and later structural and functional outcomes.

In the present study, we used a neonatal mouse model of LPS-induced sepsis-like brain injury to determine whether early NAC pretreatment followed by continued treatment improves survival and attenuates brain injury. We specifically examined whether NAC-associated protection is linked to restoration of redox balance, reduced leukocyte-associated brain infiltration, and attenuation of markers associated with neurovascular dysfunction, and whether these effects are accompanied by improved white matter-related and neurobehavioral outcomes. By administering NAC before LPS exposure and maintaining treatment thereafter, we adopted a prevention-oriented proof-of-concept design to determine whether early modulation of redox homeostasis could increase the resilience of the developing brain to subsequent inflammatory insult. This approach allowed us to characterize the neuroprotective profile of NAC, with particular focus on the redox–neurovascular–leukocyte axis of injury.

## Materials and methods

### Animals and ethical approval

C57BL/6J male and female mice (8–10 weeks old) were obtained from Janvier Labs (Paris, France) and housed under specific-pathogen-free conditions in a temperature- and humidity-controlled environment with a 12-h light/dark cycle. All experimental procedures were approved by the Gothenburg Animal Ethics Committee (approval no. 6165/24) and were performed in accordance with Swedish national guidelines (SJVFS 2019:9).

Litters were monitored daily, and postnatal day 0 (PND0) was defined as the day of birth. On PND3, pups weighing < 1.7 g were temporarily excluded and re-evaluated after 12–24 h, whereas pups weighing > 2.5 g were excluded to minimize variability in brain injury outcomes. In total, 9 pups were excluded based on these predefined criteria. A total of 477 pups (244 males and 233 females) were included in the study. Both sexes were included and considered as a biological variable where appropriate.

Pups were randomly allocated to experimental groups using a random number generator, with individuals from each litter distributed across groups to ensure balanced sex representation whenever feasible. Investigators were blinded to group allocation throughout all stages of data acquisition and analysis, including behavioral testing, image analysis, biochemical assays, and data processing. Litter distribution was considered during group assignments to avoid overrepresentation of any single litter within an experimental group.

### Experimental design and treatment

To model neonatal LPS-induced sepsis-like inflammatory injury and evaluate the neuroprotective effects of NAC, pups were assigned to four experimental groups: control (CON), NAC, LPS, and NAC + LPS.

On PND3, pups in the CON group received intraperitoneal (i.p.) saline (8 µL/g), followed by subcutaneous saline (6 µL/g) on the same day and daily i.p. saline (4 µL/g) from PND4 to PND14. Pups in the NAC group received NAC (200 mg/kg, i.p.) on PND3, followed by subcutaneous saline and daily NAC treatment (100 mg/kg/day, i.p.) from PND4 to PND14. Pups in the LPS group received i.p. saline pretreatment (8 µL/g), followed by subcutaneous LPS (3 mg/kg; 6 µL/g) and daily i.p. saline from PND4 to PND14. Pups in the NAC + LPS group received NAC (200 mg/kg, i.p.) 2 h prior to LPS exposure, followed by daily NAC treatment (100 mg/kg/day, i.p.) from PND4 to PND14.

Ultrapure LPS (*Escherichia coli* O55:B5; InvivoGen, tlrl-pb5lps; lot #9588-45-02) with an endotoxin potency of 3.3 × 10^6^ EU/mg according to the manufacturer’s Endotoxin Level Certificate, was prepared at 0.5 mg/mL in sterile saline. The LPS dose was selected based on preliminary experiments evaluating mortality outcomes (Supplementary Fig. S1). NAC (Sigma-Aldrich, A7250) was freshly dissolved in distilled water (25 mg/mL; pH 6.8–7.2) before each use. The NAC dosing regimen (200 mg/kg loading dose followed by 100 mg/kg/day maintenance treatment) was selected based on previous neonatal and perinatal studies demonstrating tolerability, central nervous system exposure, antioxidant activity, and neuroprotective effects in inflammatory and hypoxic-ischemic brain injury models [29, 32–34]. Preliminary experiments further demonstrated lower mortality with NAC pretreatment compared with post-treatment paradigms (Supplementary Table S1). The treatment period from P3 to P14 was selected to encompass a critical developmental window of active oligodendrocyte maturation and myelination in the neonatal mouse brain [37].

### Survival and growth monitoring

Survival was recorded daily from PND3 to PND21. Body weight was measured every 24 h using a calibrated digital scale. Growth trajectories were analyzed in the overall cohort and stratified by sex.

### Tissue collection and histology

At PND4 and PND21, pups were anesthetized with sodium pentobarbital and transcardially perfused with phosphate-buffered saline (PBS). Brains were post-fixed overnight in 5% buffered formaldehyde at 4 °C, dehydrated in graded ethanol, embedded in paraffin, and sectioned sagittally. Section  (5 μm) were collected at 150 μm intervals (PND4) or 250 μm intervals (PND21).

For immunohistochemistry, sections were deparaffinized, rehydrated, and subjected to antigen retrieval in citrate buffer (10 mM, pH 6.0). Endogenous peroxidase activity was quenched with 3% H₂O₂, and nonspecific binding was blocked with 4% donkey serum in PBS. Sections were incubated overnight at 4 °C with primary antibodies against MBP, cleaved caspase-3, GFAP, galectin-3, PDGFR-α, MPO, Ki-67, and DCX (details provided in Supplementary Table S2).

Sections were then incubated with the appropriate biotinylated secondary antibodies (1:200; Vector Laboratories) for 60 min at room temperature. Immunoreactivity was visualized using the VECTASTAIN ABC Elite system with 3,3′-diaminobenzidine (DAB) and nickel enhancement. Nuclear Fast Red (Vector Laboratories, ZF1226) was used as a counterstain where indicated.

### Quantitative image analysis

GFAP-, galectin-3-, PDGFR-α-, cleaved caspase-3-, and MPO-positive cells were quantified within a defined area corresponding to one visual field using a 10× objective in the cerebral cortex, subcortical white matter (SCWM), and hippocampus across three consecutive sagittal sections. These regions were selected based on their recognized vulnerability to neonatal inflammatory brain injury and their association with later neurodevelopmental impairment [38, 39]. The markers evaluated at 24 h were used to assess glial activation, leukocyte infiltration, and oligodendrocyte lineage vulnerability following neonatal inflammatory injury [12, 38]. Cell density was expressed as cells/mm².

Markers evaluated at PND21, including MBP, Ki-67, and doublecortin (DCX), were selected to assess myelination, cell proliferation, and neurodevelopmental alterations during the later recovery phase. Ki-67-positive cells and DCX immunoreactivity were quantified in the dentate gyrus (DG) across three consecutive sagittal sections using a 10× objective. Ki-67 was expressed as cells per millimeter of granule cell layer (GCL), whereas DCX was expressed as the ratio of positive area to GCL area. SCWM volume was estimated by summing MBP-immunoreactive areas across eight sagittal Sect. (1.25× objective) according to the formula: *V* = *SA* × *p* × *T*, where *SA* is the summed area, *p* is the inverse sampling frequency, and *T* is section thickness [13, 40]. Neocortical area was quantified from three consecutive sagittal Sect. (1.25× objective) and expressed as mm². All image analyses were performed in ImageJ by investigators blinded to group allocation.

### Immunoblotting

Cortical tissue was homogenized on ice, and protein concentration was determined using a bicinchoninic acid (BCA) assay. Samples were prepared in LDS buffer with reducing agent, heated at 70 °C for 10 min, separated by SDS-PAGE, and transferred to nitrocellulose membranes.

Membranes were incubated overnight at 4 °C with primary antibodies against cleaved caspase-3, MMP-9, albumin, NLRP3, SOD2, catalase, claudin-5, and β-actin (details provided in Supplementary Table S3). After incubation with the appropriate horseradish peroxidase-conjugated secondary antibodies (1:2,000, Vector). Immunoreactive bands were visualized using Super Signal West Pico PLUS chemiluminescent substrate (Thermo Fisher Scientific, 34580) and an LAS-3000 cooled CCD camera (Fujifilm, Japan).

### Measurement of glutathione redox balance, lipid peroxidation, and nitrosative stress

Cortical homogenates were used to assess glutathione (GSH), malondialdehyde (MDA), and 3-nitrotyrosine (3-NT) using commercially available assay kits (Abcam; ab239709, ab118970, and ab116691, respectively) according to the manufacturers’ instructions. Values were normalized to protein concentration. GSH and MDA levels were expressed as nmol/mg protein, whereas 3-NT levels were expressed as ng/mg protein. All analyses were performed by investigators blinded to group allocation.

### Behavioral assessments

Behavioral testing was conducted at fixed times to minimize circadian variability. Males and females were tested separately, and investigators were blinded.

#### Short-term sensorimotor tests (PND8)

For the negative geotaxis test (NGT), pups were placed head-down on a 45° incline, and the time required to rotate 180° was recorded. For the surface righting reflex (SRR) test, latency to turn from the supine to the prone position was measured [41, 42]. Each test was repeated three times with at least 15 min between trials.

#### Long-term behavioral tests (PND60)

Open field testing (OFT) was used to assess locomotor activity and anxiety-related behavior. Total distance traveled, time spent in the center zone, distance traveled in the center zone, and the number of center entries were recorded during a 10-min trial [43]. Novel object recognition (NOR) testing was used to assess recognition memory. Discrimination indices were calculated on the basis of time spent exploring the novel versus familiar object and the number of visits to each object [44, 45]. The testing arena was cleaned with 70% ethanol between trials.

### Statistical analysis

Statistical analyses were performed using SPSS (version 27) and GraphPad Prism (version 10). Survival was analyzed using Kaplan–Meier curves and log-rank tests. Body weight trajectories were analyzed using linear mixed-effects models with Sidak post hoc tests. Pups were distributed across treatment groups within litters at the design stage; however, litter-level clustering was not explicitly modeled in all pup-level analyses. For most endpoints, two-way ANOVA was used to evaluate the effects of group and sex and their interaction. Group comparisons were then assessed by one-way ANOVA in the overall sample, with sex-stratified one-way analyses performed as exploratory analyses. Normality was assessed using the Shapiro–Wilk test prior to parametric analyses, and homogeneity of variance was evaluated using Levene’s test. When significant differences were detected, post hoc comparisons were conducted using Tukey’s or Games–Howell tests, as appropriate. When assumptions of normality were not met, Kruskal–Wallis test followed by Dunn’s correction was used. Behavioral outliers were screened using the Z-score method (|Z| ≥ 3), and individual outlying data points were excluded when identified [46]. No formal outlier exclusion was applied to histological, Western blot, or ELISA datasets. Data are presented as mean ± SD, as specified in the figure legends. Statistical significance was defined as *P* < 0.05. Sample sizes were based on preliminary data and previous studies [13].

## Results

### NAC increases survival after neonatal LPS challenge but only modestly improves postnatal growth

**Fig. 1:**
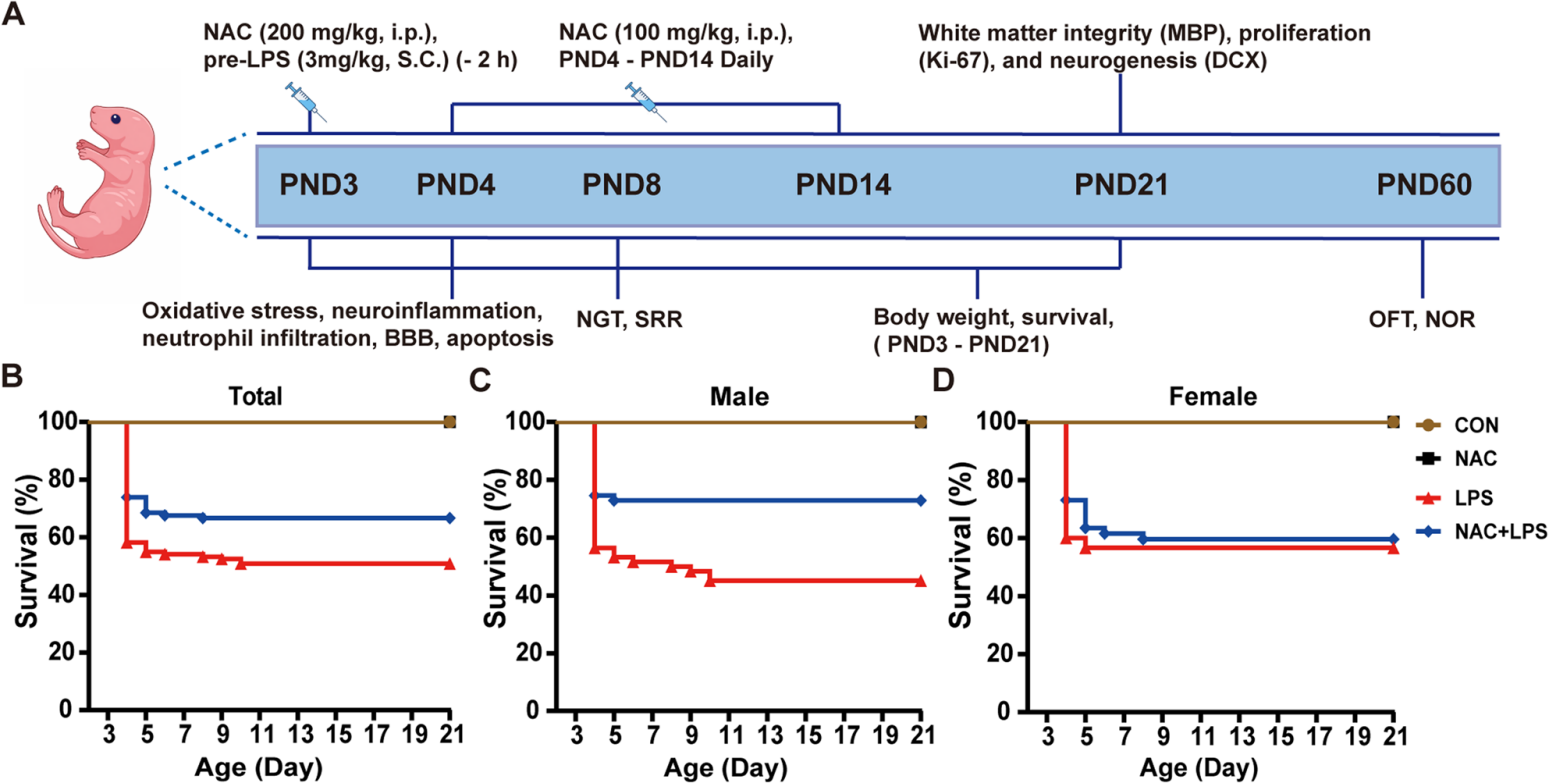
NAC enhances survival after neonatal LPS exposure.(**A**) Schematic overview of the study design and timing of treatments and outcome assessments. (**B**) Kaplan–Meier survival curves for the full cohort. No mortality occurred in the CON or NAC groups. Survival was significantly reduced after LPS exposure compared with controls (*P* < 0.001), whereas NAC treatment significantly improved survival in LPS-exposed pups (*P* < 0.05 vs. LPS). (**C**) Kaplan–Meier survival curves for male pups. LPS markedly reduced survival relative to controls (*P* < 0.001), and NAC significantly increased survival compared with the LPS group (*P* < 0.05). (**D**) Kaplan–Meier survival curves for female pups. LPS significantly reduced survival compared with controls (*P* < 0.001), but NAC did not significantly improve survival in females. Survival was analyzed using Kaplan–Meier curves with log-rank testing. Group sizes were as follows: males, CON (*n* = 33), NAC (*n* = 34), LPS (*n* = 62), NAC + LPS (*n* = 59); females, CON (*n* = 32), NAC (*n* = 33), LPS (*n* = 60), NAC + LPS (*n* = 52). Abbreviations: BW, body weight; ELISA, enzyme-linked immunosorbent assay; IHC, immunohistochemistry; NGT, negative geotaxis test; NOR, novel object recognition; OFT, open field test; PND, postnatal day; SRR, surface righting reflex; WB, western blot; i.p., intraperitoneal; s.c., subcutaneous

An overview of the experimental design is shown (Fig. 1A). Neonatal LPS exposure caused marked mortality and impaired postnatal growth. Survival was significantly lower in LPS-treated pups than in saline controls (50.8% vs. 100%, *P* < 0.001; Fig. 1B). NAC treatment increased survival in LPS-exposed animals to 66.7% (*P* < 0.05 vs. LPS), indicating a protective effect in this model. Exploratory sex-specific analyses suggested that this survival benefit was driven mainly by males, in whom survival rose from 45.2% in the LPS group to 72.9% with NAC (Fig. 1C), whereas no clear improvement was detected in females (56.7% vs. 59.6%; Fig. 1D).

LPS also produced sustained postnatal growth restriction. In both sexes, body weight trajectories diverged from controls beginning at PND4 and remained lower through PND21(Supplementary Fig. S2A–C). NAC treatment modestly lessened this growth impairment, but the effect was limited, as body weights in NAC-treated LPS pups did not return to control levels and improvement in weight gain by PND21 was small. Overall, these findings show that NAC meaningfully improves survival after neonatal LPS challenge while providing only partial benefit for LPS-associated postnatal growth failure.

### NAC improves cortical redox status after neonatal LPS exposure without altering antioxidant enzyme expression

**Fig. 2:**
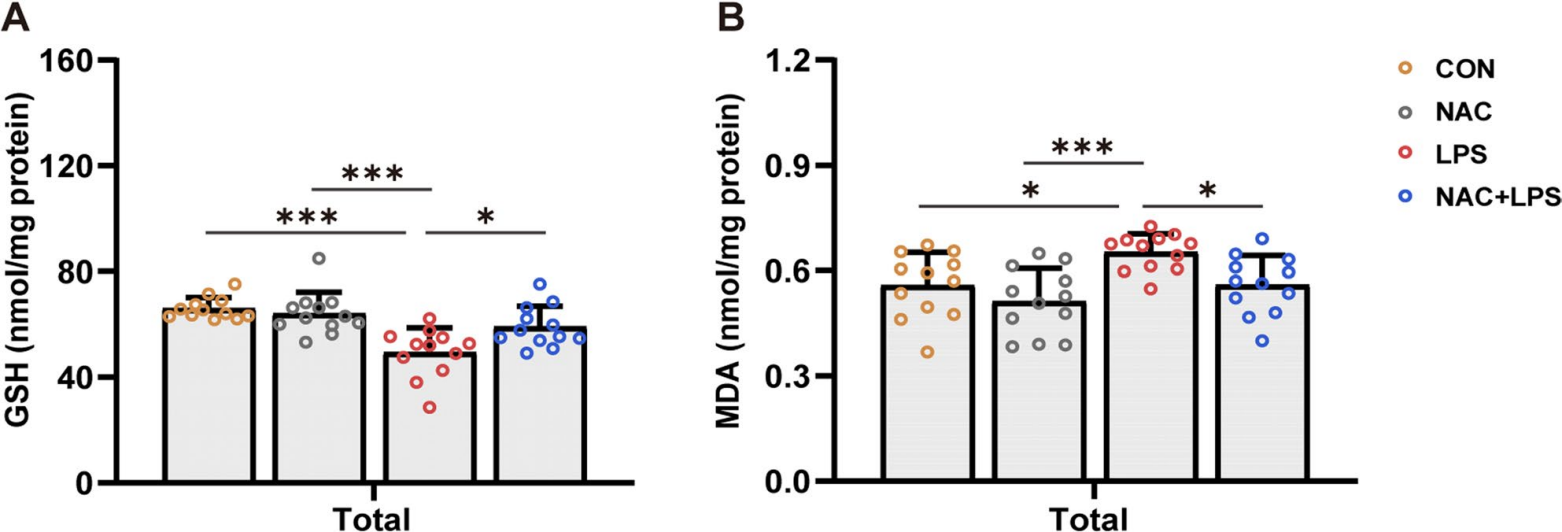
NAC partially restores cortical glutathione and reduces lipid peroxidation after neonatal LPS exposure. (**A**) Glutathione (GSH) levels in the cerebral cortex at 24 h after treatment in the four experimental groups (*n* = 12 per group; 6 males and 6 females). (**B**) Malondialdehyde (MDA) levels in the cerebral cortex at 24 h after treatment in the four experimental groups (*n* = 12 per group; 6 males and 6 females). Data are presented as mean ± SD. Effects of treatment group, sex, and their interaction were evaluated by two-way ANOVA. Between-group comparisons were performed using one-way ANOVA followed by Tukey’s post hoc tests. *P < 0.05, ***P < 0.001

To evaluate the effect of NAC on oxidative stress in the neonatal cortex after LPS exposure, we measured GSH, MDA, and 3-NT. LPS challenge disrupted cortical redox balance, as shown by lower GSH levels and higher MDA levels compared with saline-treated controls. NAC treatment significantly increased GSH relative to the LPS group (Fig. 2A), although levels did not fully normalize, and also reduced MDA to near-control values (Fig. 2B), indicating attenuation of lipid peroxidation. In contrast, 3-NT levels were not significantly different between groups (Supplementary Fig. S3A). Analyses by sex showed no significant group-by-sex interaction for GSH, MDA, or 3-NT (Supplementary Fig. S3B–D).

We next asked whether these changes were accompanied by altered expression of major antioxidant enzymes in the cortex. Representative immunoblots of superoxide dismutase 2 (SOD2) and catalase are shown (Supplementary Fig. S3E). Immunoblot analysis showed no significant differences in SOD2 or catalase levels across experimental groups in either males or females (Supplementary Fig. S3F–I). Together, these findings indicate that NAC alleviates LPS-induced oxidative imbalance in the neonatal cortex primarily by partially replenishing glutathione and reducing lipid peroxidation, without detectable changes in nitrative stress or in the expression of SOD2 and catalase at the time point examined.

### NAC has little effect on early glial activation or cortical NLRP3 expression after neonatal LPS challenge

**Fig. 3:**
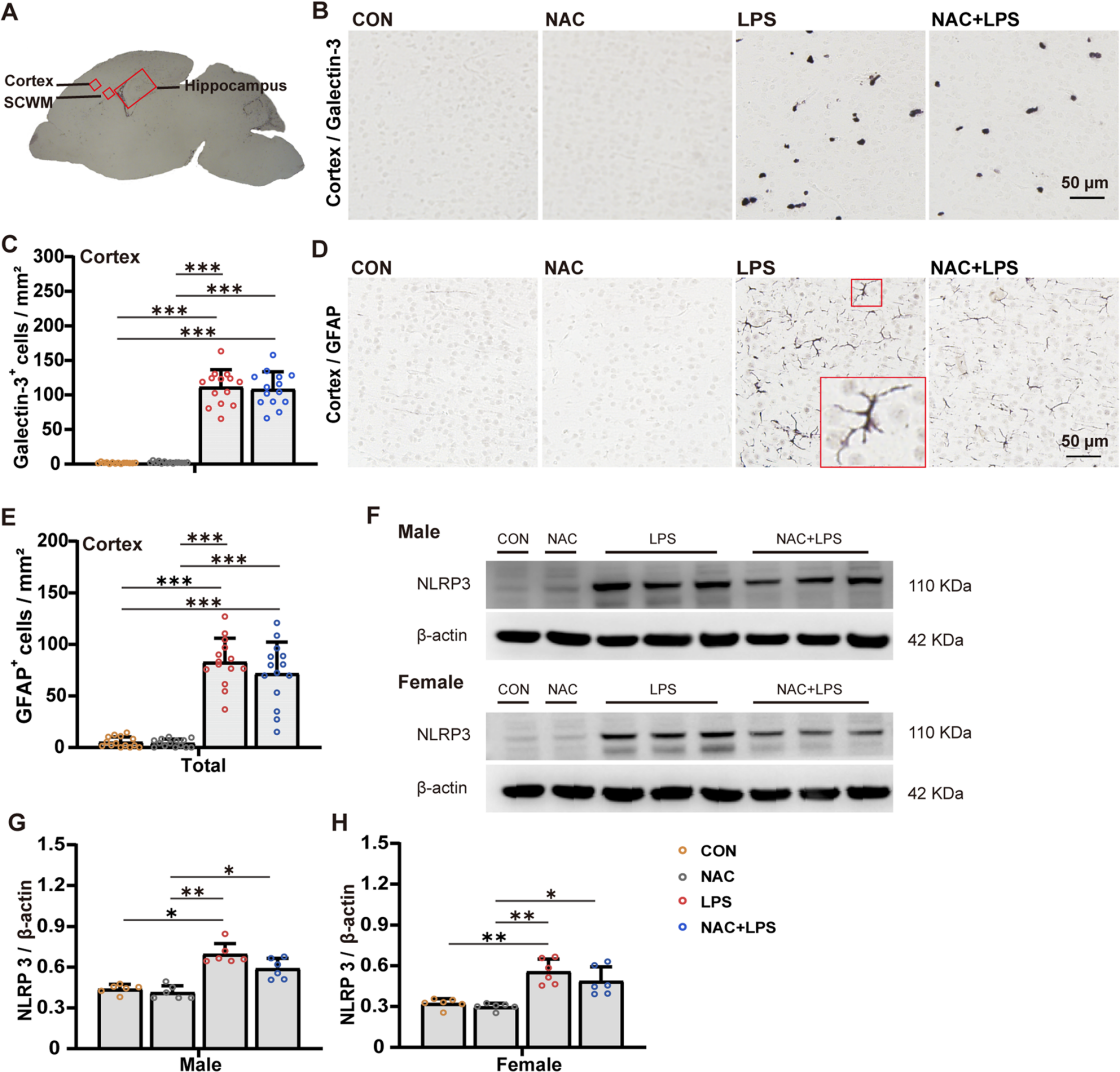
NAC does not alter LPS-induced galectin-3, GFAP, or cortical NLRP3 expression 24 h after neonatal LPS exposure. (**A**) Schematic illustrating the brain regions used for quantitative analysis. (**B**) Representative immunohistochemical images of galectin-3 staining in sagittal sections of the cerebral cortex. (**C**) Galectin-3-positive cells in the cerebral cortex at 24 h after treatment in the four experimental groups (*n* = 14 per group; 7 males and 7 females). (**D**) Representative immunohistochemical images of GFAP staining in sagittal sections of the cerebral cortex. (**E**) GFAP-positive cells in the cerebral cortex at 24 h after treatment in the four experimental groups (*n* = 14 per group; 7 males and 7 females). (**F**) Representative immunoblots of NLRP3 in the cerebral cortex. (**G**–**H**) Densitometric analysis of cortical NLRP3 expression normalized to β-actin in male and female pups at 24 h after treatment (*n* = 6 per group per sex). Data are presented as mean ± SD. Effects of treatment group, sex, and their interaction for GFAP and Galectin-3 were evaluated by two-way ANOVA. Between-group comparisons were performed using one-way ANOVA with Games–Howell post hoc tests, or Kruskal–Wallis analysis with Dunn’s post hoc correction when parametric assumptions were not met. *P < 0.05, **P < 0.01, ***P < 0.001

To determine whether NAC attenuates the early inflammatory response in the neonatal brain, we examined galectin-3-positive cells, GFAP-positive cells, and cortical NLRP3 protein levels 24 h after LPS administration. The region of interest used for quantification is shown (Fig. 3A). Representative galectin-3 immunohistochemical staining in sagittal sections of the cerebral cortex, SCWM, CA, and DG are presented (Fig. 3B; Supplementary Fig. S4A). LPS triggered a robust increase in galectin-3-positive cells across several brain regions, including the cerebral cortex, SCWM, CA, and DG, confirming a widespread glial inflammatory response (Fig. 3C; Supplementary Fig. S4B–D). However, NAC treatment did not significantly lower galectin-3-positive cell numbers in any of the regions analyzed. These effects were similar in males and females, with no significant group-by-sex interactions detected (Supplementary Fig. S4E–H).

A comparable pattern was observed for GFAP. Representative GFAP immunohistochemical staining in sagittal sections of the cerebral cortex and SCWM is presented (Fig. 3D; Supplementary Fig. S5A). LPS markedly increased GFAP-positive cell counts in both the cerebral cortex and SCWM, whereas NAC did not significantly modify this response relative to LPS alone (Fig. 3E; Supplementary Fig. S5B). Again, there was no evidence of sex-dependent treatment effects (Supplementary Fig. S5C–D).

We also assessed cortical NLRP3 expression to examine whether NAC influenced inflammasome-related signaling at this early time point. Representative immunoblots of NLRP3 are shown (Fig. 3F). Although LPS increased NLRP3 protein expression relative to controls in both sexes, NAC did not significantly reduce cortical NLRP3 levels (Fig. 3G–H). Overall, these findings indicate that, at 24 h after neonatal LPS exposure, NAC did not measurably suppress the glial inflammatory markers or inflammasome-related protein expression evaluated in this study.

### NAC limits cortical leukocyte accumulation and reduces markers of BBB disruption after neonatal LPS challenge

**Fig. 4:**
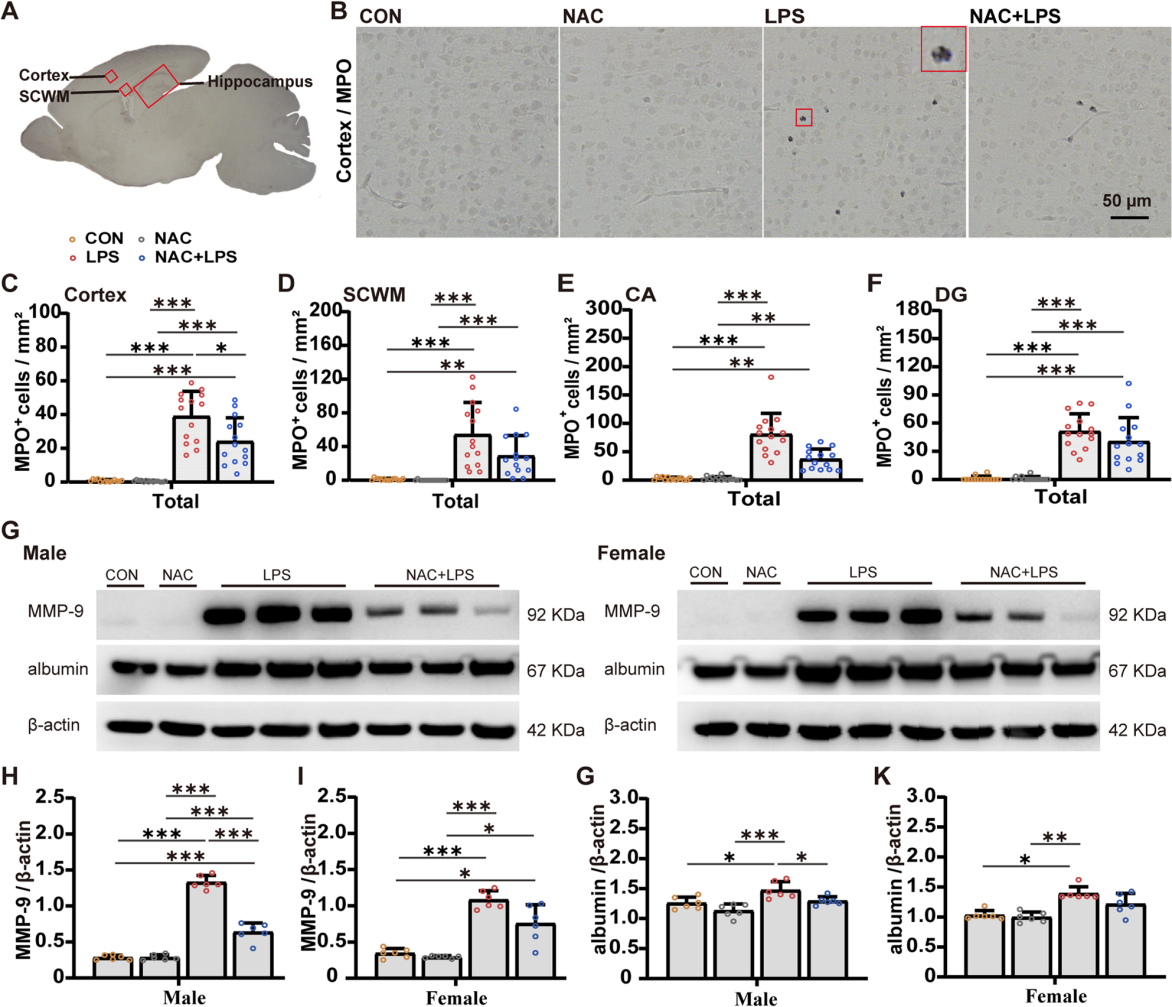
NAC reduces cortical MPO-positive cell accumulation and attenuates LPS-induced alterations in proteins associated with BBB integrity. (**A**) Schematic illustrating the brain regions used for quantitative analysis. (**B**) Representative immunohistochemical images of myeloperoxidase (MPO) staining in sagittal sections of the cerebral cortex. (**C**–**F**) MPO-positive cells in the cerebral cortex, subcortical white matter (SCWM), cornu ammonis (CA), and dentate gyrus (DG) at 24 h after treatment in the four experimental groups (*n* = 14 per group; 7 males and 7 females). (**G**) Representative immunoblots of matrix metalloproteinase-9 (MMP-9) and albumin in the cerebral cortex. (**H**–**K**) Densitometric analysis of cortical MMP-9 and albumin expression normalized to β-actin in male and female pups at 24 h after treatment (*n* = 6 per group per sex). Data are presented as mean ± SD. Effects of treatment group, sex, and their interaction were assessed by two-way ANOVA. Between-group comparisons were performed using one-way ANOVA with Tukey’s or Games–Howell post hoc tests, or Kruskal–Wallis analysis with Dunn’s post hoc correction when parametric assumptions were not met. *P < 0.05, **P < 0.01, ***P < 0.001

To investigate whether NAC affects leukocyte entry into the brain and neurovascular injury after neonatal LPS exposure, we quantified myeloperoxidase (MPO)-positive cells in multiple brain regions and examined cortical levels of matrix metallopeptidase-9 **(**MMP-9), albumin, and claudin-5. The region of interest used for quantification is shown (Fig. 4A). Representative MPO immunohistochemical staining in sagittal sections of the cerebral cortex, SCWM, CA, and DG are presented (Fig. 4B; Supplementary Fig. S6A). LPS markedly increased MPO-positive cell accumulation in the cerebral cortex, SCWM, CA, and DG compared with controls. NAC significantly lowered MPO-positive cell numbers in the cerebral cortex (Fig. 4C), but not in the other regions examined (Fig. 4D–F). No significant sex-dependent treatment effects were detected for MPO-positive cell counts (Supplementary Fig. S6B–E).

We next evaluated markers related to BBB integrity in the cortex. In cortical immunoblot analyses, representative immunoblots of MMP-9, albumin, and claudin-5 are shown (Fig. 4G; Supplementary Fig. S6F). LPS increased MMP-9 expression and albumin extravasation, consistent with BBB-associated injury. In exploratory sex-stratified analyses, NAC reduced both MMP-9 and albumin levels in males, whereas these effects were not evident in females (Fig. 4H–K). In contrast, claudin-5 expression was not significantly altered by LPS or NAC in either sex at this time point (Supplementary Fig. S6G–H). Taken together, these findings indicate that after neonatal LPS exposure, NAC reduced cortical MPO-positive cell accumulation and attenuated changes in MMP-9 and albumin, two indicators of neurovascular injury and BBB-associated pathology. These effects occurred in the absence of detectable changes in claudin-5 expression at the time point examined.

### NAC attenuates LPS-induced apoptotic injury, with stronger effects in exploratory analyses of males

**Fig. 5:**
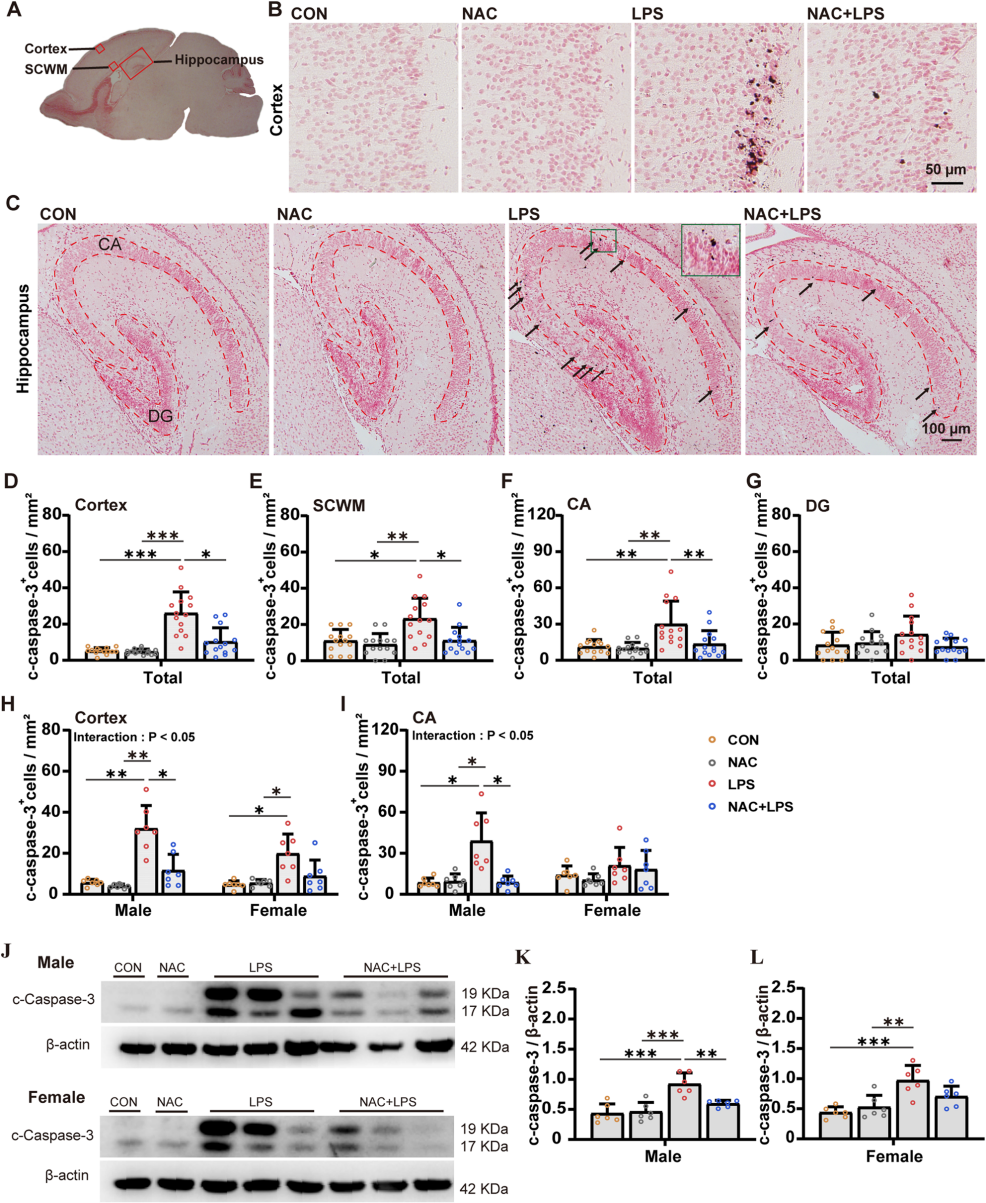
NAC reduces cleaved caspase-3 expression after neonatal LPS exposure. (**A**) Schematic illustrating the brain regions used for apoptotic cell quantification. (**B**–**C**) Representative immunohistochemical images of cleaved caspase-3 (c-caspase-3) staining in sagittal sections of the cerebral cortex and hippocampus. The red outline indicates the cornu ammonis (CA) and dentate gyrus (DG) regions, and arrows indicate c-caspase-3-positive cells. (**D**–**G**) C-caspase-3-positive cells in the cerebral cortex, subcortical white matter (SCWM), CA, and DG at 24 h after treatment in the four experimental groups (*n* = 14 per group; 7 males and 7 females). (**H**–**I**) Sex-stratified quantification of c-caspase-3-positive cells in the cerebral cortex and CA at 24 h after treatment (*n* = 7 per group per sex). (**J**) Representative immunoblots of c-caspase-3 in the cerebral cortex. (**K–L**) Densitometric analysis of cortical c-caspase-3 expression normalized to β-actin in male and female pups at 24 h after treatment (*n* = 6 per group per sex). For Western blot analyses, male and female samples were run and normalized separately; therefore, sex-stratified one-way ANOVA was used for these datasets. Effects of treatment group, sex, and their interaction were evaluated by two-way ANOVA, with significant interactions indicated above the corresponding panels when present. Between-group comparisons were performed using one-way ANOVA with Tukey’s or Games–Howell post hoc tests, or Kruskal–Wallis analysis with Dunn’s post hoc correction when parametric assumptions were not met. *P < 0.05, **P < 0.01, ***P < 0.001

We next examined whether NAC influenced cell death after neonatal LPS exposure by quantifying cleaved caspase-3 (c-caspase-3)-positive cells in multiple brain regions (Fig. 5A). Representative c-caspase-3 immunohistochemical staining in sagittal sections of the cerebral cortex, CA, DG and SCWM are presented (Fig. 5B–C; Supplementary Fig. S7A). LPS increased apoptotic cell counts in the cerebral cortex, SCWM, and CA, whereas no significant change was detected in the DG (Fig. 5D–G). NAC treatment significantly lowered c-caspase-3-positive cell numbers in the cerebral cortex, SCWM, and CA, but did not affect the DG.

When sex was taken into account, significant group-by-sex interactions were detected in the cerebral cortex and CA (Fig. 5H–I), but not in SCWM or DG (Supplementary Fig. S7B–C). Exploratory sex-stratified analyses indicated that the anti-apoptotic effect of NAC in the cerebral cortex and CA was mainly evident in males, whereas corresponding reductions were not detected in females.

These histological findings were supported by cortical protein analyses. Representative immunoblots of c-caspase-3 are shown (Fig. 5J). LPS increased c-caspase-3 expression in both sexes, and NAC reduced cortical c-caspase-3 levels in males but not in females (Fig. 5K–L). Overall, these data indicate that NAC limits apoptotic injury after neonatal LPS exposure in selected brain regions, with some protective effects appearing more pronounced in exploratory analyses of male pups.

### NAC provides partial protection of oligodendrocyte-related and white matter-associated outcomes after neonatal LPS exposure

**Fig. 6:**
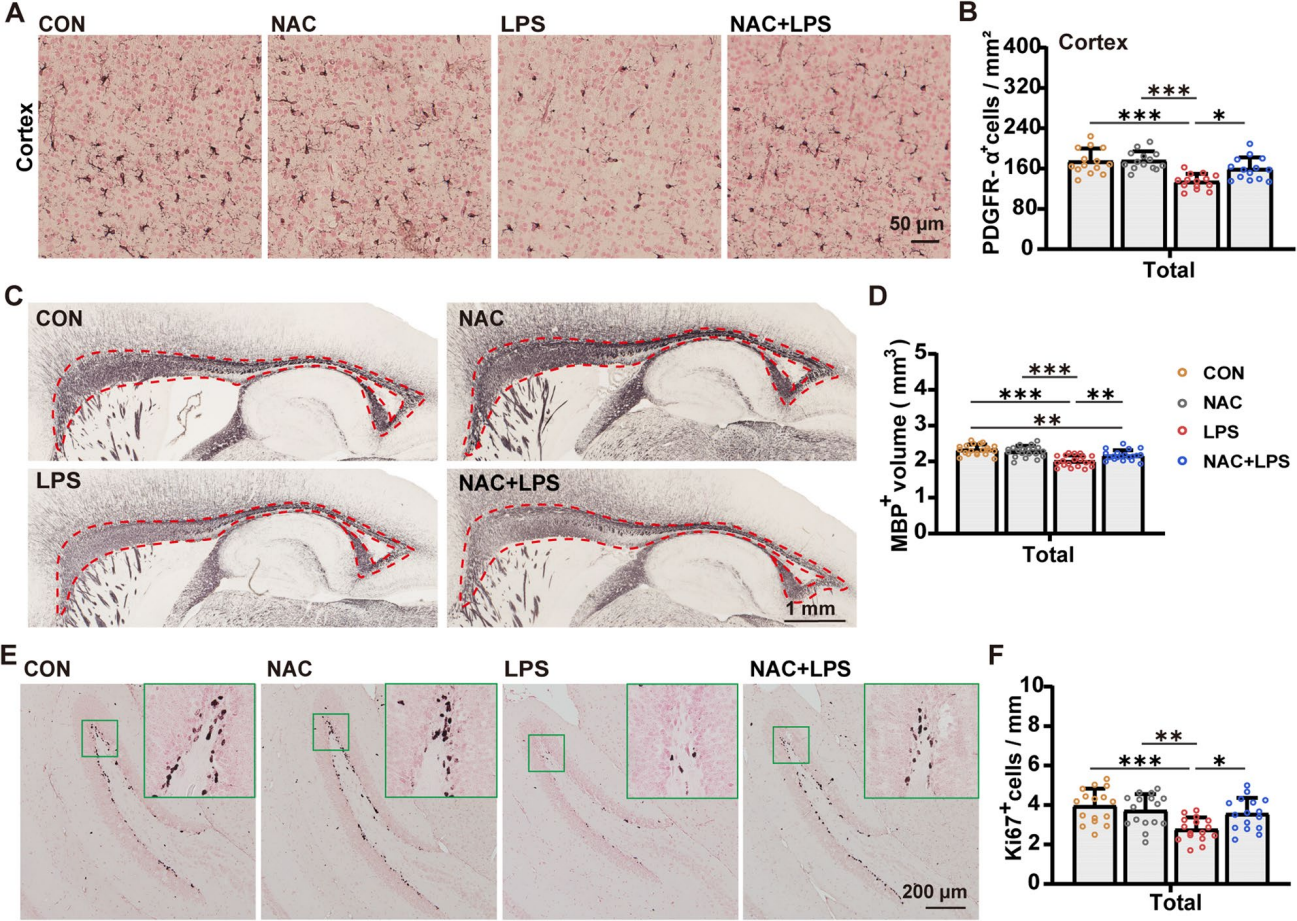
NAC partially improves oligodendrocyte lineage-, myelin-, and proliferation-related outcomes after neonatal LPS exposure. (**A**) Representative immunohistochemical images of platelet-derived growth factor receptor-α (PDGFR-α) staining in sagittal sections of the cerebral cortex. (**B**) Quantification of PDGFR-α-positive cells in the cerebral cortex at 24 h after LPS exposure in the four experimental groups (*n* = 14 per group; 7 males and 7 females). (**C**) Representative immunohistochemical images of myelin basic protein (MBP) staining in sagittal sections of the subcortical white matter (SCWM); the red outline indicates the analyzed region. (**D**) MBP-positive volume in the SCWM at postnatal day 21 (PND21) in the four experimental groups (*n* = 20 per group; 10 males and 10 females). (**E**) Representative immunohistochemical images of Ki-67 staining in sagittal sections of the dentate gyrus (DG). (**F**) Ki-67-positive cells in the DG at PND21 in the four experimental groups (*n* = 16 per group; 8 males and 8 females). Effects of treatment group, sex, and their interaction were assessed by two-way ANOVA. Between-group comparisons were performed using one-way ANOVA with Tukey’s post hoc tests. *P < 0.05, **P < 0.01, ***P < 0.001

To determine whether NAC influenced early oligodendrocyte lineage cells, we quantified platelet-derived growth factor receptor-α (PDGFR-α)-positive oligodendrocyte precursor cells in the cerebral cortex and SCWM 24 h after LPS administration. The region of interest used for quantification is shown (Supplementary Fig. S8A). PDGFR-α immunohistochemical staining in sagittal sections of the cerebral cortex and SCWM are presented (Fig. 6A and Supplementary Fig. S8B). LPS reduced the number of PDGFR-α-positive cells in the cerebral cortex, whereas NAC significantly increased these counts relative to the LPS group (Fig. 6B), indicating partial preservation of the OPC population. In subcortical white matter, changes in PDGFR-α-positive cell numbers were less pronounced, and NAC did not produce a significant effect (Supplementary Fig. S8C). No sex-dependent treatment effects were detected in either region (Supplementary Fig. S8D–E).

We next assessed longer-term white matter-related changes at PND21. MBP immunohistochemical staining in sagittal sections of the SCWM is presented (Fig. 6C). In the subcortical white matter, LPS reduced MBP-positive volume, consistent with impaired myelination, and NAC partially restored this measure (Fig. 6D). No sex-dependent treatment effects were detected in either region (Supplementary Fig. S8F). Neocortical area in sagittal sections is presented (Supplementary Fig. S8G). Quantification of neocortical area revealed no significant differences among the four experimental groups in either the overall analysis or sex-stratified analyses (Supplementary Fig. S8H–I). These findings suggest that NAC confers partial protection against LPS-associated myelin loss without significant effects on overall cortical tissue preservation at the time point examined.

Because neonatal inflammatory injury may also affect neurogenic regions [47], we examined cell proliferation and immature neuronal markers in the DG. Ki-67 immunohistochemical staining in sagittal sections of the DG is presented (Fig. 6E). LPS significantly reduced the number of Ki-67-positive proliferating cells, whereas NAC restored proliferation relative to the LPS group (Fig. 6F). No sex-dependent treatment effects were detected in either region (Supplementary Fig. S8J). DCX immunohistochemical staining in sagittal sections of the DG is presented (Supplementary Fig. S8K). In contrast, DCX immunoreactivity did not differ among groups (Supplementary Fig. S8L-M), indicating no detectable effect on this measure of immature neurons at the time point examined.

Overall, these data show that NAC partially preserves oligodendrocyte lineage and white matter-related indices after neonatal LPS exposure and supports DG cell proliferation, without measurable changes in DCX immunoreactivity.

### NAC improves early geotaxis performance but does not affect other behavioral measures examined

**Fig. 7:**
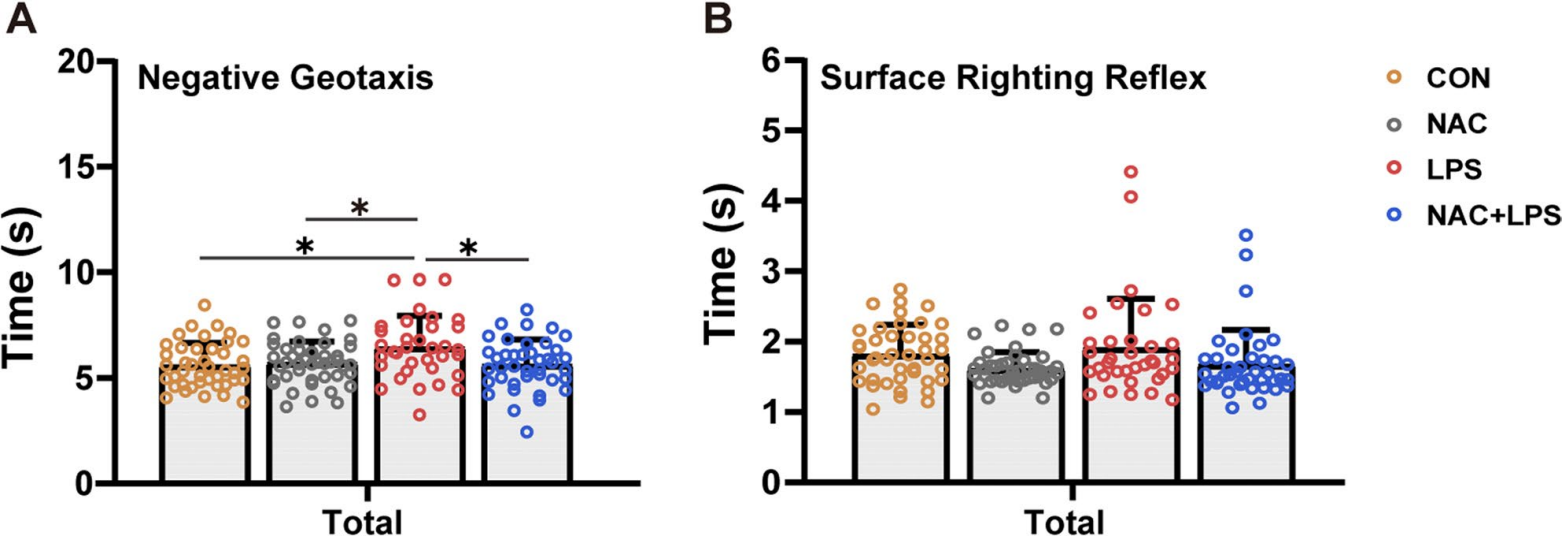
NAC improves negative geotaxis performance but does not alter surface righting at PND8 after neonatal LPS exposure. (**A**–**B**) Latency to complete the negative geotaxis test and surface righting reflex at postnatal day 8 (PND8) in the four experimental groups (*n* = 37–44 per group; 17–23 males and 19–22 females). Effects of treatment group, sex, and their interaction were evaluated by two-way ANOVA. Between-group comparisons were performed using one-way ANOVA with Tukey’s post hoc tests, or Kruskal–Wallis analysis with Dunn’s post hoc correction when parametric assumptions were not met. *P < 0.05

Early sensorimotor development was evaluated at PND8 using negative geotaxis and surface righting tests. LPS exposure impaired performance in the negative geotaxis task (Fig. 7A), indicating an early deficit in sensorimotor function. NAC treatment improved this deficit, bringing geotaxis latency closer to control values. In contrast, surface righting performance was similar across groups (Fig. 7B), indicating no detectable effect of either LPS or NAC on this reflex at the age tested. These outcomes were not modified by sex (Supplementary Fig. S9A–B).

To determine whether early treatment influenced later behavior, animals were also assessed at PND60 in the open field test and novel object recognition paradigm (Supplementary Fig. S9C). No group differences were detected in locomotor activity or anxiety-related exploratory behavior in the open field, including total movement and measures related to center-zone exploration (Supplementary Fig. S9D–G). Likewise, recognition memory performance in the novel object recognition task was unchanged across groups, whether assessed by exploration time or visit frequency (Supplementary Fig. S9H–I). No sex-dependent effects were identified in these long-term behavioral analyses (Supplementary Fig. S9J–O).

Overall, these data suggest that NAC provides a modest early functional benefit in sensorimotor performance after inflammation, but this improvement was not accompanied by detectable changes in the later behavioral outcomes examined.

## Discussion

This study identifies early NAC pretreatment followed by continued treatment as a partially effective neuroprotective intervention in neonatal LPS-induced sepsis-like brain injury. NAC improved survival, restored key aspects of cortical redox balance, reduced cortical MPO-positive cell accumulation, and attenuated markers associated with neurovascular injury, accompanied by reductions in apoptosis and partial preservation of white matter-related outcomes. In contrast, NAC did not significantly reduce galectin-3, GFAP, or NLRP3 at 24 h and did not improve long-term performance in the behavioral tests used here. Overall, these findings are consistent with a role for a redox-neurovascular-leukocyte mechanisms in NAC-associated neuroprotection rather than through broad suppression of the measured glial inflammatory response.

The redox data support glutathione restoration as a central mechanism of protection. LPS depleted cortical glutathione and increased lipid peroxidation [8], whereas NAC partially reversed both changes [17, 18]. This is biologically plausible in the immature brain, where antioxidant defenses are limited and oligodendrocyte lineage cells are highly vulnerable to oxidative injury [25, 48]. The absence of changes in SOD2 and catalase suggests that NAC acted mainly by replenishing antioxidant capacity rather than by inducing endogenous antioxidant enzymes. Similarly, the lack of effect on 3-nitrotyrosine indicates that NAC did not uniformly suppress all oxidative or nitrosative pathways at this time point. Instead, its action appears selective and sufficient to reduce downstream tissue injury.

A second key finding is that NAC attenuated LPS induced molecular changes associated with neurovascular and leukocyte-associated injury. Specifically, NAC reduced cortical MPO-positive leukocyte accumulation and decreased both MMP-9 expression and albumin extravasation, suggesting attenuation of neurovascular injury-related processes. Although these measures do not provide direct evidence of BBB preservation, they are consistent with evidence that systemic inflammation in the immature brain promotes BBB breakdown, leukocyte recruitment, and secondary tissue injury [9, 12, 13], and with prior work suggesting that NAC can inhibit neutrophil trafficking into the developing brain [36]. The absence of an effect on claudin-5 may indicate that it is not markedly affected at this time point, or that tight junction alterations occur in a temporally or regionally specific manner [49]. Nevertheless, the overall pattern is consistent with a contribution of neurovascular mechanisms to the observed protective effects of NAC [50]. These effects were most evident in the cortex, suggesting regional specificity in both injury mechanisms and treatment response [51, 52].

Notably, NAC did not significantly alter galectin-3-positive cells, GFAP-positive cells, or cortical NLRP3 expression at 24 h despite improving redox balance and neurovascular-related outcomes after LPS. This apparent dissociation may reflect the temporal and compartment-specific nature of neonatal neuroinflammation, although the possibility that NAC exerts limited effects on some inflammatory pathways at this time point cannot be excluded [53, 54]. NAC may act preferentially on early redox-sensitive vascular and leukocyte pathways, while glial markers examined remain unchanged at the sampled time point. The present findings are therefore consistent with a selective rather than global anti-inflammatory effect [55].

The white matter data reinforce the biological relevance of this protective profile. NAC preserved cortical PDGFR-α-positive oligodendrocyte precursor cells early after injury and partially restored MBP-positive volume in subcortical white matter at PND21. These findings are consistent with the idea that limiting early oxidative and vascular injury helps maintain a more permissive environment for oligodendrocyte lineage progression and later myelination [26, 29, 35]. Restoration of dentate gyrus Ki-67-positive cell proliferation, without change in DCX immunoreactivity, further suggests that NAC supports selective reparative processes rather than broadly enhancing developmental plasticity.

Functionally, NAC improved negative geotaxis at PND8 but did not alter surface righting, open field behavior, or novel object recognition. This pattern suggests that early biochemical and histological protection translated into modest short-term sensorimotor benefit but was insufficient to produce detectable effects in the later behavioral battery used here. Such dissociation between early biological indicators of neuroprotection and later neurobehavioral outcomes has been reported in neonatal brain injury research, highlighting the complexity of translating early molecular or structural improvements into sustained functional benefit [56]. Early reduction of oxidative, neurovascular, and white matter injury may preserve selected cellular populations without fully preventing circuit-level alterations that influence later cognition and behavior. In addition, developmental compensation in rodents [57], limited sensitivity of the behavioral assays [58–60], and survivor bias related to substantial early mortality may have reduced the ability to detect long-term functional differences. Therefore, absence of significant behavioral improvement in the present study should not necessarily be interpreted as absence of biologically meaningful neuroprotection.

Exploratory analyses suggested possible male-biased benefit for survival and selected BBB- and apoptosis-related outcomes. Although these signals were not uniform and should be interpreted cautiously, they are consistent with evidence that male and female neonatal brains differ in inflammatory signaling [61], oxidative stress handling [62], and cell death pathways [63]. The present data do not support a broadly sex-specific action of NAC, but they may indicate potential sex-dependent differences that require confirmation in adequately powered studies.

Several limitations should be acknowledged. First, the LPS model captures endotoxin-driven inflammation but does not fully reproduce the complexity of clinical neonatal sepsis. Second, because NAC was administered before LPS exposure and continued thereafter, the study should be viewed as a prevention-oriented proof-of-concept paradigm rather than a therapeutic model of established sepsis. Mechanistic analyses were limited, with most assessments focused on the cortex, a single early acute time point, and selected inflammatory and cell death markers. Therefore, the temporal evolution of injury, the specific identity of MPO-positive leukocytes, and the potential contribution of non-apoptotic cell death pathways remain to be defined. In addition, lateral ventricular enlargement was not quantified because measurements from sagittal sections may be influenced by ventricular asymmetry and sampling bias. Long-term interpretation is further limited by substantial early mortality and the use of a relatively narrow behavioral test battery. Although LPS exposure represents an acute inflammatory insult, mortality extended beyond the immediate post-exposure period, indicating that the consequences of neonatal LPS exposure were not restricted to the acute phase. To reduce litter-related confounding, pups from each litter were distributed across treatment groups with sex balanced whenever feasible. However, litter was not included as a random factor in all statistical models. Therefore, residual litter-related influences cannot be completely excluded, although treatment allocation within litters was implemented to minimize this source of confounding. Finally, the study was not powered to detect sex-dependent effects across all endpoints, and the sex-stratified findings should therefore be considered exploratory. Future studies using post-insult treatment paradigms, broader molecular profiling, and more sensitive long-term functional assessments are warranted.

This study demonstrates that early NAC pretreatment followed by continued treatment provides partial neuroprotection in neonatal sepsis-like brain injury, with its most prominent effects linked to restoration of redox balance, attenuation of leukocyte-associated injury, and attenuation of neurovascular injury-associated changes. These pathways and injury processes are particularly relevant to the vulnerable preterm brain, in which oxidative stress and neurovascular dysfunction are key contributors to white matter injury [8, 9]. From a clinical standpoint, NAC may represent an attractive candidate because it is already clinically used, has a known safety profile, and can access the central nervous system [30, 31, 64, 65]. The findings raise the possibility that targeting redox imbalance and neurovascular injury, in addition to inflammatory pathways, may represent a promising therapeutic strategy for neonatal sepsis-associated brain injury. However, the limited impact on long-term behavioral outcomes and the use of a pretreatment paradigm highlights the need for further studies to define optimal timing, dosing, and patient selection. Importantly, whether similar neuroprotective effects can be achieved when NAC is administered after the onset of injury remains to be determined. Overall, these results are consistent with the potential of NAC as part of an early preventive neuroprotective strategy in neonatal sepsis, particularly for preserving white matter integrity.

## Conclusion

In this neonatal mouse model of LPS-induced sepsis-like brain injury, early NAC pretreatment followed by continued treatment improved survival and provided partial neuroprotection. This protection was mainly associated with restoration of redox balance and reduced MPO-positive leukocyte accumulation, and attenuation of neurovascular injury-related markers, while the glial inflammatory markers examined were not significantly affected at the assessed time point.

Together, these findings suggest that interactions involving redox imbalance, neurovascular injury, and leukocyte-associated responses may contribute to the observed neuroprotective effects of NAC. The present findings provide a rationale for further evaluation of NAC as a preventive neuroprotective strategy in high-risk preterm neonatal populations.

## Supplementary Information

Supplementary Material 1.

Supplementary Material 2.

## Data Availability

The datasets generated and/or analyzed during the current study are available from the corresponding author upon reasonable request.

## Abbreviations

BBB: Blood-brain barrier
BCA: Bicinchoninic acid
BW: Body weight
CA: Cornu ammonis
DAB: 3,3’-diaminobenzidine
DCX: Doublecortin
DG: Dentate gyrus
ELISA: Enzyme-linked immunosorbent assay
GCL: Granule cell layer
GSH: Glutathione
IHC: Immunohistochemistry
LPS: Lipopolysaccharide
MBP: Myelin basic protein
MDA: Malondialdehyde
MMP-9: Matrix metalloproteinase-9
MPO: Myeloperoxidase
NAC: N-acetylcysteine
3-NT: 3-nitrotyrosine
NGT: Negative geotaxis test
NOR: Novel object recognition
OFT: Open field test
OPC: Oligodendrocyte precursor cell
PBS: Phosphate-buffered saline
PDGFR-α: Platelet-derived growth factor receptor-α
PND: Postnatal day
SCWM: Subcortical white matter
SOD2: Superoxide dismutase 2
SRR: Surface righting reflex
WB: Western blot
