# Supplementary material for: N-Acetylcysteine protects the developing brain in neonatal sepsis-like inflammation via a redox–neurovascular pathway
Source: J Neuroinflammation. 2026 Jun 30;23:222. doi: 10.1186/s12974-026-03942-9 (PMC13317334; doi:10.1186/s12974-026-03942-9)
Supplement: Supplementary file 1 — Supplementary Material 1. [file 12974_2026_3942_MOESM1_ESM.docx]

**Supplementary Material 1**

[**Supplementary Table S1** Effect of NAC administration on mortality in PND3 mice.2](#_Toc226103513)

**Supplementary Table S2** Primary antibodies for immunohistochemistry…………2 **Supplementary Table S3** Primary antibodies for immunoblotting 3

**Supplementary Figure S1** Dose-dependent effect of LPS on mortality in PND3 mice.4

[**Supplementary Figure S2** Effects of NAC on growth after neonatal LPS exposure... 4](#_Toc226103514)

**Supplementary Figure S3** Effects of NAC on cortical oxidative stress markers after neonatal LPS exposure 5

**Supplementary Figure S4** Effects of NAC on galectin-3 after neonatal LPS exposure 6

**Supplementary Figure S5** Effects of NAC on GFAP after neonatal LPS exposure…7

**Supplementary Figure S6** Effects of NAC on MPO-positive cell infiltration and claudin-5 expression after neonatal LPS exposure 8

**Supplementary Figure S7** Effects of NAC on cleaved caspase-3 after neonatal LPS exposure 9

**Supplementary Figure S8** NAC effects on oligodendrocyte lineage markers, myelination, cortical tissue preservation, and neurodevelopmental alterations following neonatal sepsis. 10

**Supplementary Figure S9** Effects of NAC on early and late behavioral outcomes after neonatal LPS exposure 12

**Supplementary Table S1.** Effect of NAC administration on mortality in PND3 mice

| **Group** | **Number of pups, N** | **Mortality, n/N (%)** | **Notes** |
| --- | --- | --- | --- |
| CON | 3 | 0/3 (0.0) | No LPS, no NAC |
| NAC | 3 | 0/3 (0.0) | NAC only, no LPS |
| LPS | 8 | 4/8 (50.0) | LPS only |
| NAC (−2 h) + LPS | 10 | 3/10 (30.0) | NAC 2 h before LPS |
| NAC (+2 h) + LPS | 8 | 3/8 (37.5) | NAC 2 h after LPS |
| NAC (+24 h) + LPS | 9 | 4/9 (44.4) | NAC 24 h after LPS |

**Notes:** Experimental groups included untreated controls (CON), NAC-treated controls (NAC), LPS only (LPS), and NAC-treated groups receiving NAC either 2 h before LPS (−2 h), 2 h after LPS (+2 h), or 24 h after LPS (+24 h). All LPS-exposed groups received a subcutaneous injection of LPS (3 mg/kg). NAC was administered intraperitoneally to pups, with an initial dose of 200 mg/kg on postnatal day 3 (PND3), followed by daily doses of 100 mg/kg from PND4 to PND14. Mortality rates (%) are presented for each group.

**Supplementary Table S2.** Primary antibodies for immunohistochemistry

| **Target** | **Host** | **Dilution** | **Company** | **Catalog number** |
| --- | --- | --- | --- | --- |
| MBP | Mouse | 1:500 | BioLegend | 836504 |
| Cleaved caspase-3 | Rabbit | 1:500 | CST | 9661 |
| GFAP | Rabbit | 1:400 | Santa Cruz | sc-6171-R |
| Galectin-3 | Rat | 1:250 | Invitrogen | 14-5301-82 |
| PDGFR-α | Rabbit | 1:500 | CST | 3164 |
| MPO | Rabbit | 1:150 | Abcam | ab9535 |
| Ki-67 | Mouse | 1:500 | BD Pharmingen | 550609 |
| DCX | Rabbit | 1:800 | Abcam | ab18723 |

**Supplementary Table S3.** Primary antibodies for immunoblotting

| **Target** | **Host** | **Dilution** | **Company** | **Catalog number** |
| --- | --- | --- | --- | --- |
| Cleaved caspase-3 | Rabbit | 1:1000 | CST | 9661 |
| MMP-9 | Rabbit | 1:1000 | Abcam | ab228402 |
| Albumin | Goat | 1:1000 | Abcam | ab19194 |
| NLRP3 | Mouse | 1:1000 | Life Science | A41812012 |
| SOD2 | Rabbit | 1:500 | ABclonal | A1340 |
| Catalase | Rabbit | 1:1000 | CST | 14097 |
| Claudin-5 | Mouse | 1:1000 | Invitrogen | 35-2500 |
| β-actin | Rabbit | 1:1000 | Sigma-Aldrich | A2066 |


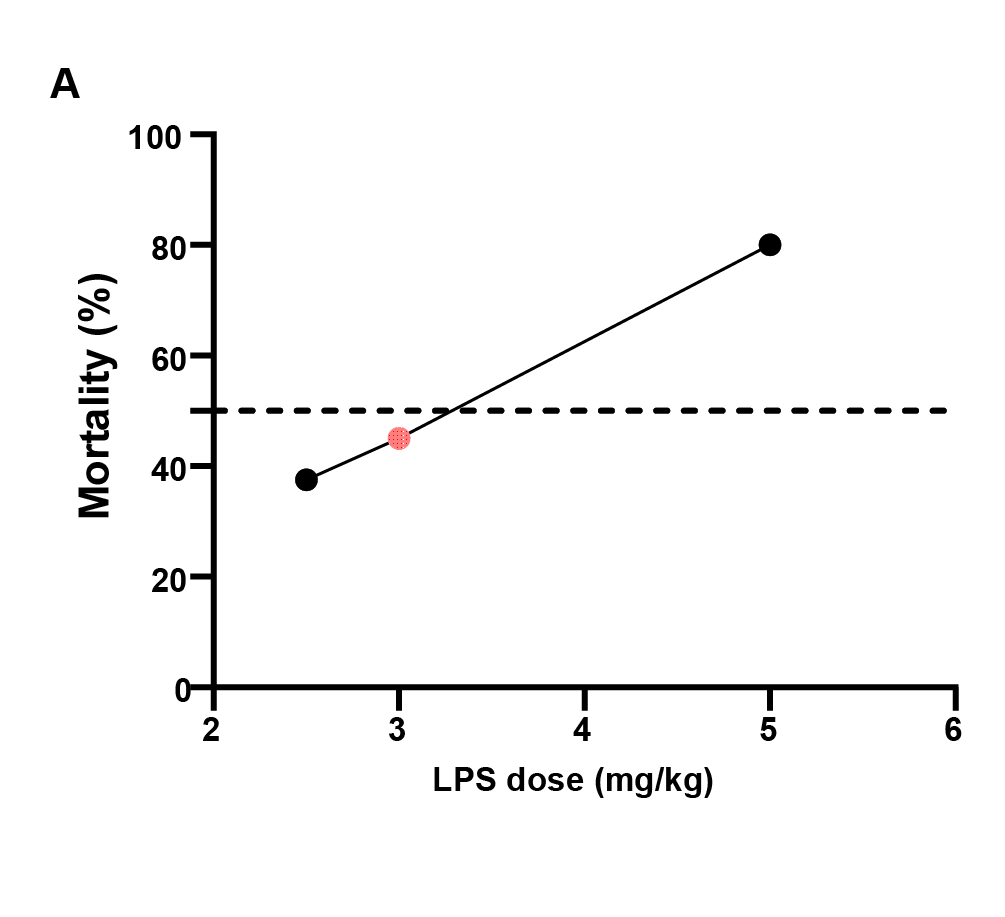


**Supplementary Figure S1. Dose-dependent effects of LPS on mortality in PND3 pups.** Mortality was assessed at PND21 following subcutaneous injection of LPS at different doses. A dose of 3 mg/kg resulted in approximately 45% mortality and was selected for subsequent experiments.


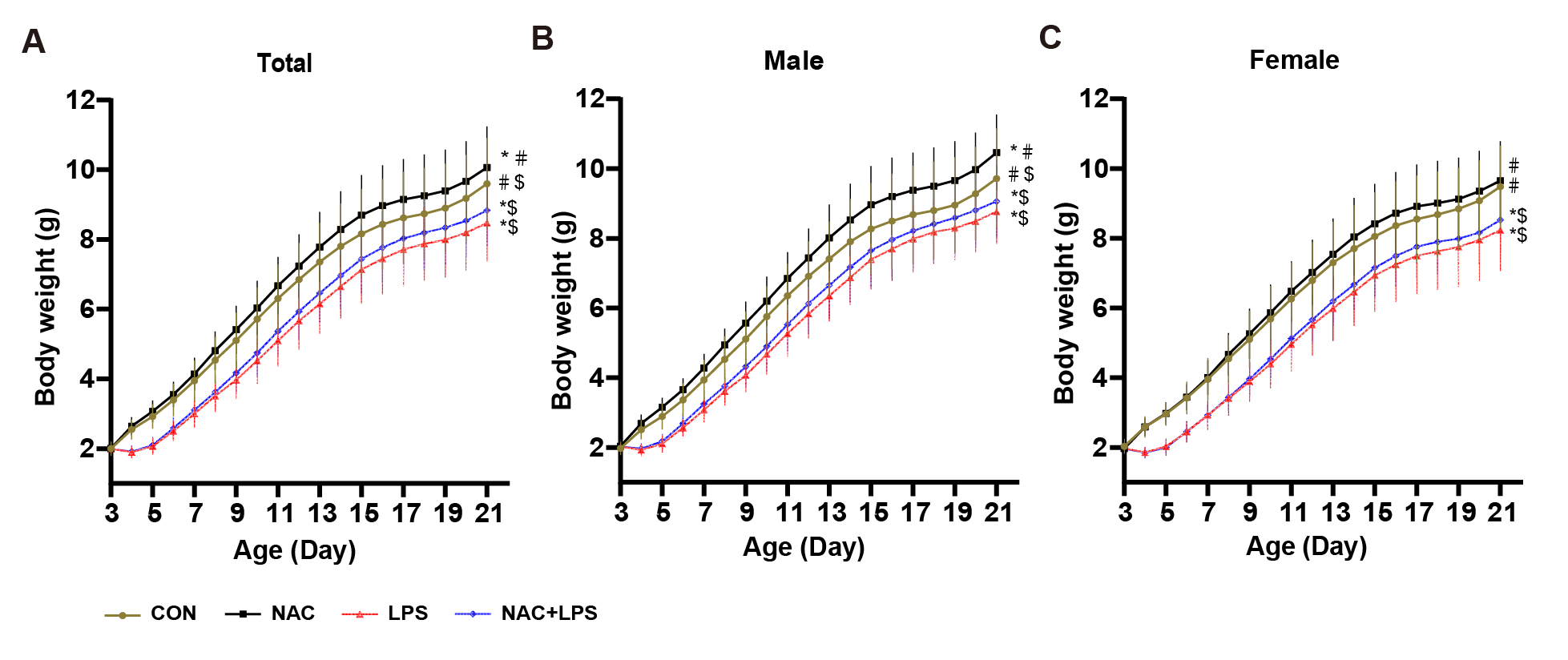


**Supplementary Figure S2. Effects of NAC on growth after neonatal LPS exposure.** (A) Body weight from postnatal day 3 (PND3) to PND21 in all animals. Significant main effects of group and group × age interaction were observed (P < 0.001). (B) Body weight from PND3 to PND21 in males. (C) Body weight from PND3 to PND21 in females. Group sizes were as follows: males, CON (n = 33), NAC (n = 34), LPS (n = 62), NAC+LPS (n = 59); females, CON (n = 32), NAC (n = 33), LPS (n = 60), NAC+LPS (n = 52). Data are presented as mean ± SD. **P* < 0.05 vs CON; #*P* < 0.05 vs LPS; $*P* < 0.05 vs NAC.


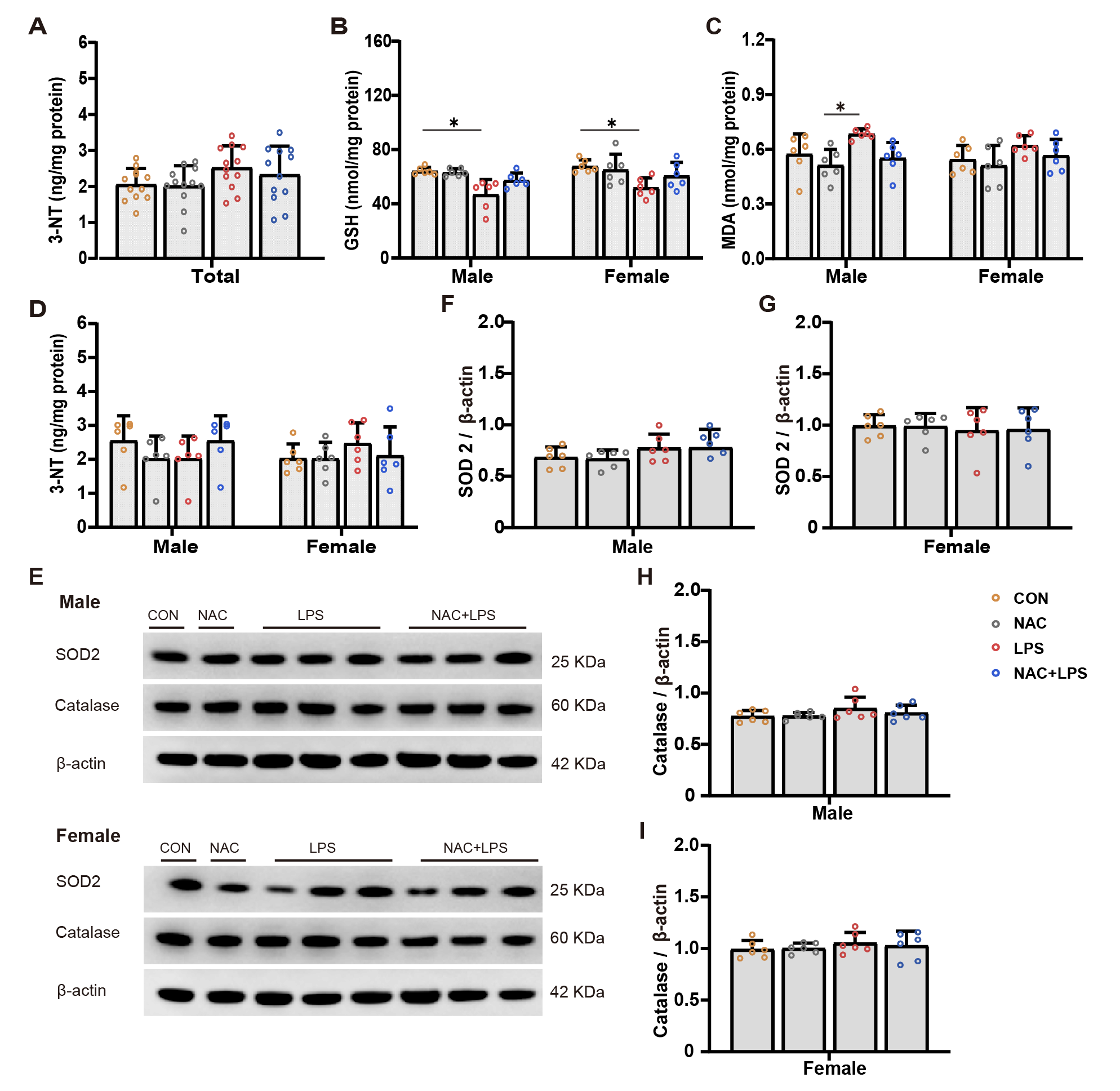


**Supplementary Figure S3. Effects of NAC on cortical oxidative stress markers after neonatal LPS exposure.** (A) 3-nitrotyrosine (3-NT) levels in the cerebral cortex at 24 h after LPS exposure in the four experimental groups (n = 12 per group; 6 males and 6 females). (B–D) Glutathione (GSH), malondialdehyde (MDA), and 3-NT levels in the cerebral cortex at 24 h after LPS exposure in male and female pups across the four groups (n = 6 per group per sex). (E) Representative immunoblots of superoxide dismutase 2 (SOD2) and catalase in the cerebral cortex. (F–I) Densitometric analysis of cortical SOD2 and catalase expression normalized to β-actin at 24 h after LPS exposure in male and female pups (n = 6 per group per sex). Data are presented as mean ± SD. Effects of treatment group, sex, and their interaction were evaluated by two-way ANOVA. Between-group comparisons were performed using one-way ANOVA followed by Tukey’s or Games–Howell post hoc tests, or by Kruskal–Wallis analysis with Dunn’s post hoc correction when parametric assumptions were not met. **P* < 0.05.


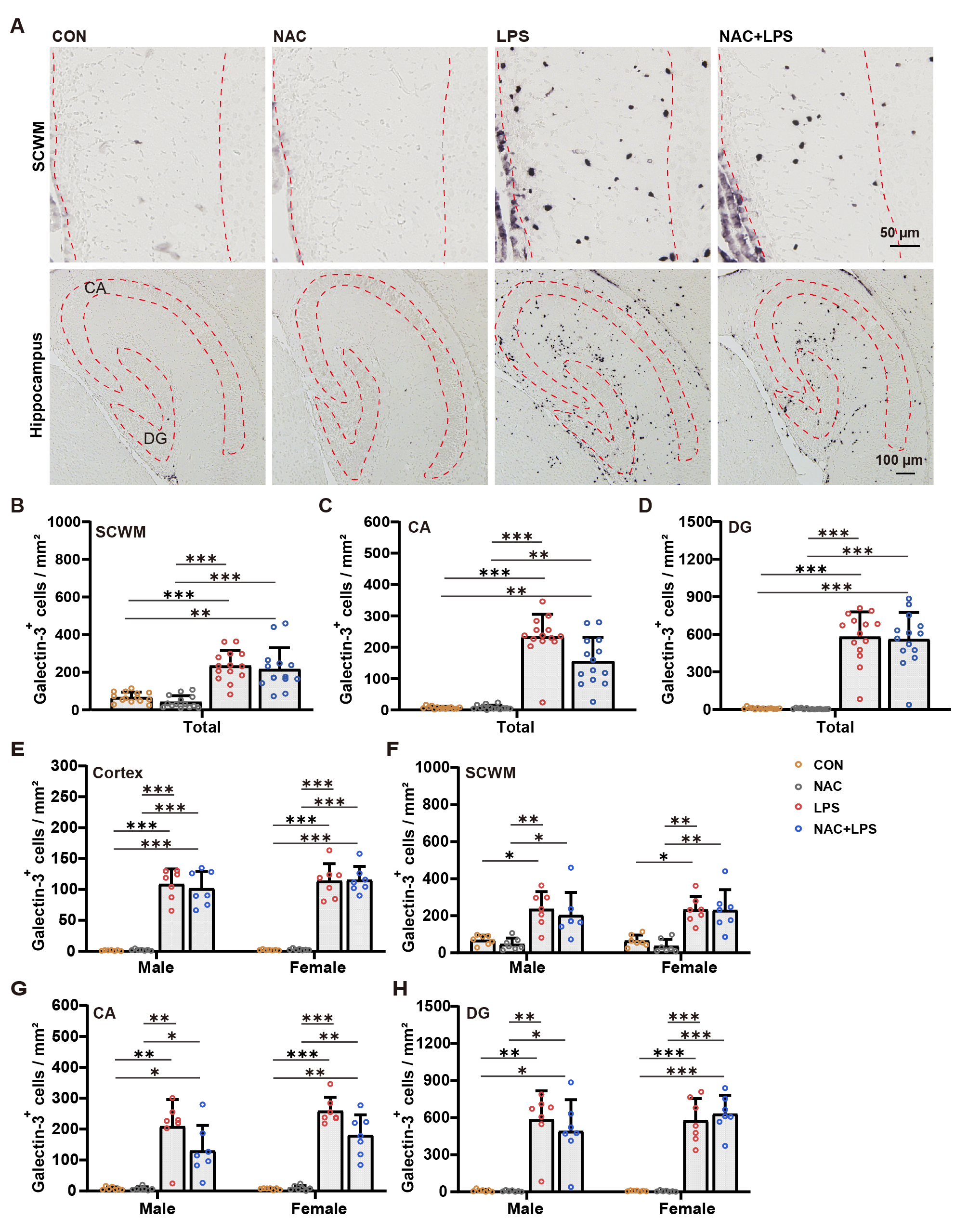


**Supplementary Figure S4. Effects of NAC on galectin-3 after neonatal LPS exposure.** (A) Representative immunohistochemical images of galectin-3 staining in sagittal sections of the subcortical white matter (SCWM) and hippocampus. The red outline indicates the SCWM, cornu ammonis (CA), and dentate gyrus (DG) regions. (B–D) Quantification of galectin-3-positive cells in the SCWM, CA, and DG at 24 h after LPS exposure in the four experimental groups (n = 14 per group; 7 males and 7 females). (E–H) Quantification of galectin-3-positive cells in the cerebral cortex, SCWM, CA, and DG at 24 h after LPS exposure in male and female pups across the four groups (n = 7 per group per sex). Data are presented as mean ± SD. Effects of treatment group, sex, and their interaction were evaluated by two-way ANOVA. Between-group comparisons were performed using one-way ANOVA followed by Games–Howell post hoc tests, or by Kruskal–Wallis analysis with Dunn’s post hoc correction when parametric assumptions were not met. **P* < 0.05, ***P* < 0.01, ****P* < 0.001.


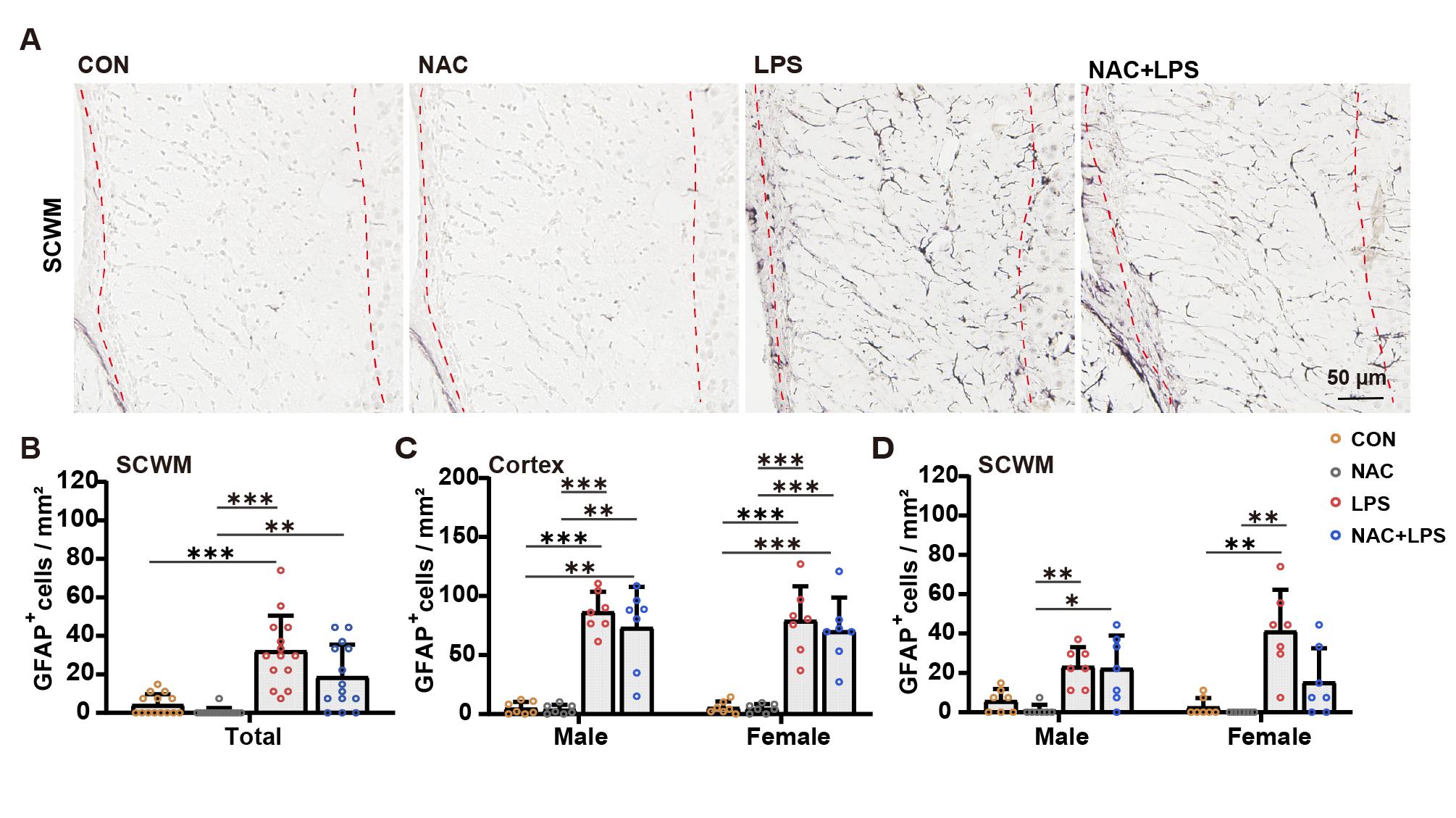


**Supplementary Figure S5. Effects of NAC on GFAP after neonatal LPS exposure.** (A) Representative immunohistochemical images of GFAP staining in sagittal sections of the subcortical white matter (SCWM). The red outline indicates the SCWM region. (B) Quantification of GFAP-positive cells in the SCWM at 24 h after LPS exposure in the four experimental groups (n = 14 per group; 7 males and 7 females). (C–D) Quantification of GFAP-positive cells in the cerebral cortex and SCWM at 24 h after LPS exposure in male and female pups across the four groups (n = 7 per group per sex). Data are presented as mean ± SD. Effects of treatment group, sex, and their interaction were evaluated by two-way ANOVA. Between-group comparisons were performed using one-way ANOVA followed by Tukey’s or Games–Howell post hoc tests, or by Kruskal–Wallis analysis with Dunn’s post hoc correction when parametric assumptions were not met. **P* < 0.05, ***P* < 0.01, ****P* < 0.001.


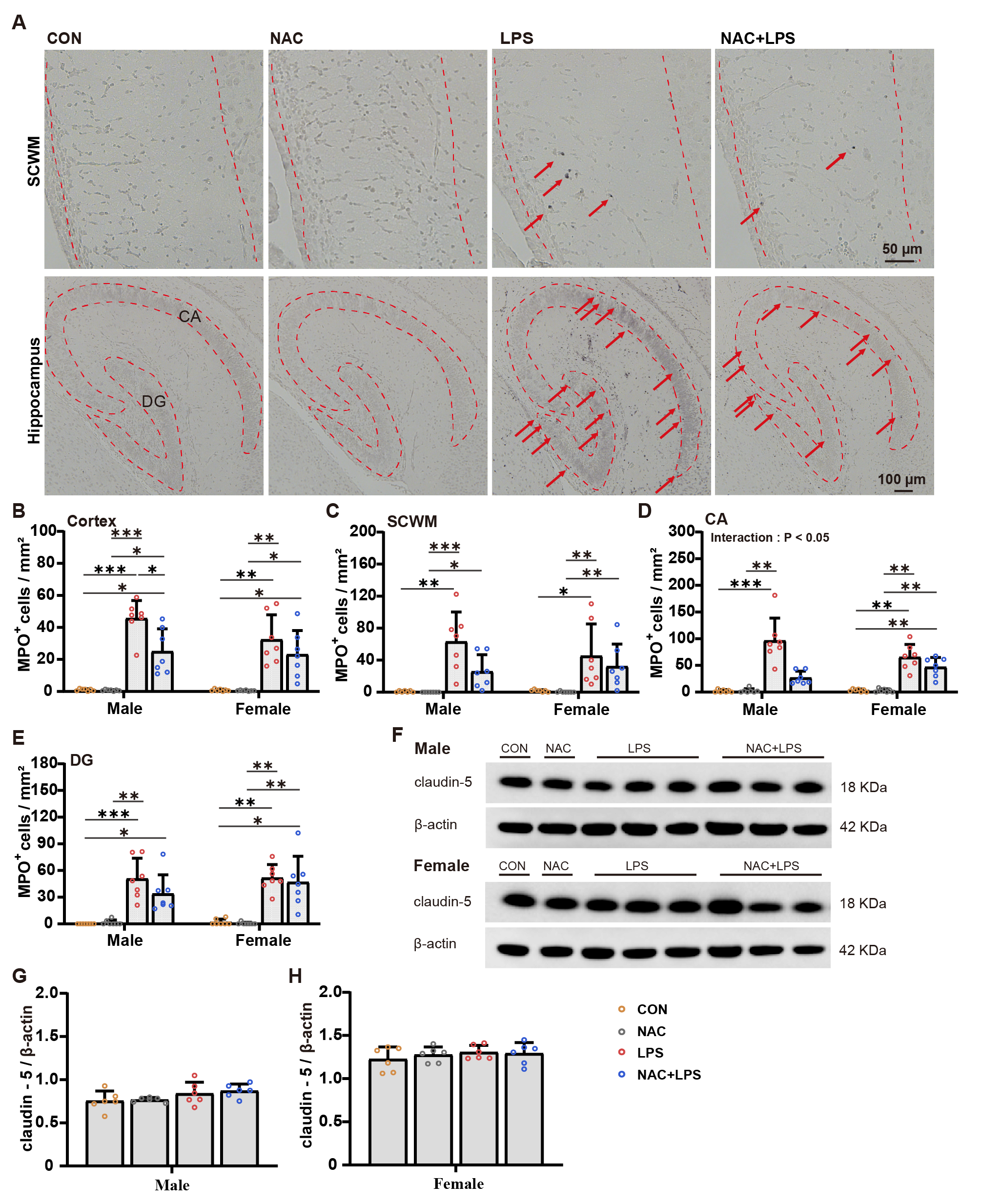


**Supplementary Figure S6. Effects of NAC on MPO-positive cell infiltration and claudin-5 expression after neonatal LPS exposure.** (A) Representative immunohistochemical images of myeloperoxidase (MPO) staining in sagittal sections of the subcortical white matter (SCWM) and hippocampus. The red outline indicates the SCWM, cornu ammonis (CA), and dentate gyrus (DG) regions, and arrows indicate MPO-positive cells. (B–E) Quantification of MPO-positive cells in the cerebral cortex, SCWM, CA, and DG at 24 h after LPS exposure in male and female pups across the four experimental groups (n = 7 per group per sex). (F) Representative immunoblots of claudin-5 in the cerebral cortex. (G–H) Densitometric analysis of cortical claudin-5 expression normalized to β-actin at 24 h after LPS exposure in male and female pups (n = 6 per group per sex). Data are presented as mean ± SD. Effects of treatment group, sex, and their interaction were evaluated by two-way ANOVA, with significant interactions indicated above the corresponding panels when present. Between-group comparisons were performed using one-way ANOVA followed by Tukey’s or Games–Howell post hoc tests, or by Kruskal–Wallis analysis with Dunn’s post hoc correction when parametric assumptions were not met. **P* < 0.05, ***P* < 0.01, ****P* < 0.001.


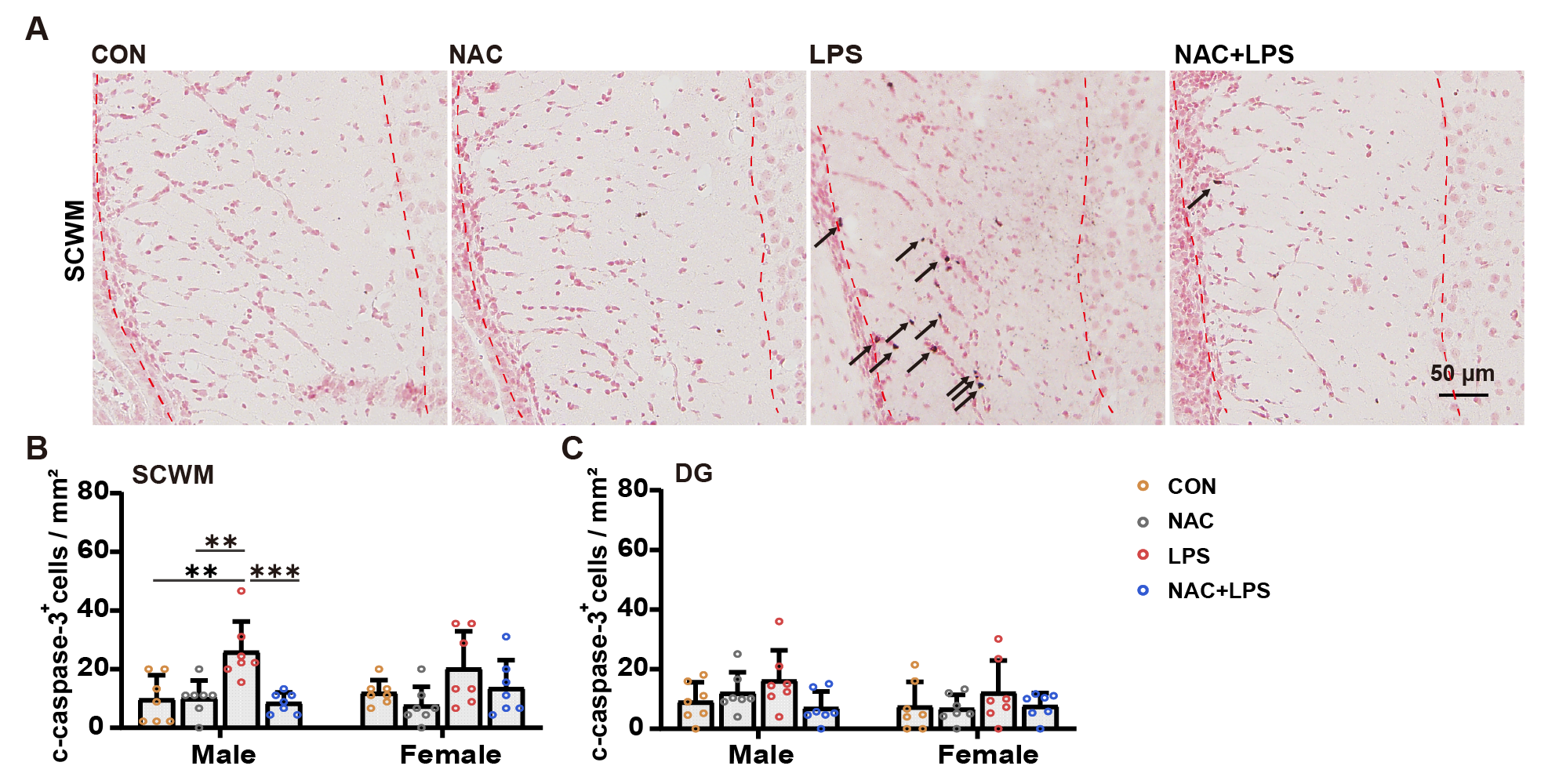


**Supplementary Figure S7. Effects of NAC on cleaved caspase-3 after neonatal LPS exposure.** (A) Representative immunohistochemical images of cleaved caspase-3 (c-caspase-3) staining in sagittal sections of the subcortical white matter (SCWM). The red outline indicates the SCWM region, and arrows indicate c-caspase-3-positive cells. (B–C) Quantification of c-caspase-3-positive cells in the SCWM and dentate gyrus (DG) at 24 h after LPS exposure in male and female pups across the four experimental groups (n = 7 per group per sex). Data are presented as mean ± SD. Effects of treatment group, sex, and their interaction were evaluated by two-way ANOVA. Between-group comparisons were performed using one-way ANOVA followed by Tukey’s or Games–Howell post hoc tests. ***P* < 0.01, ****P* < 0.001.


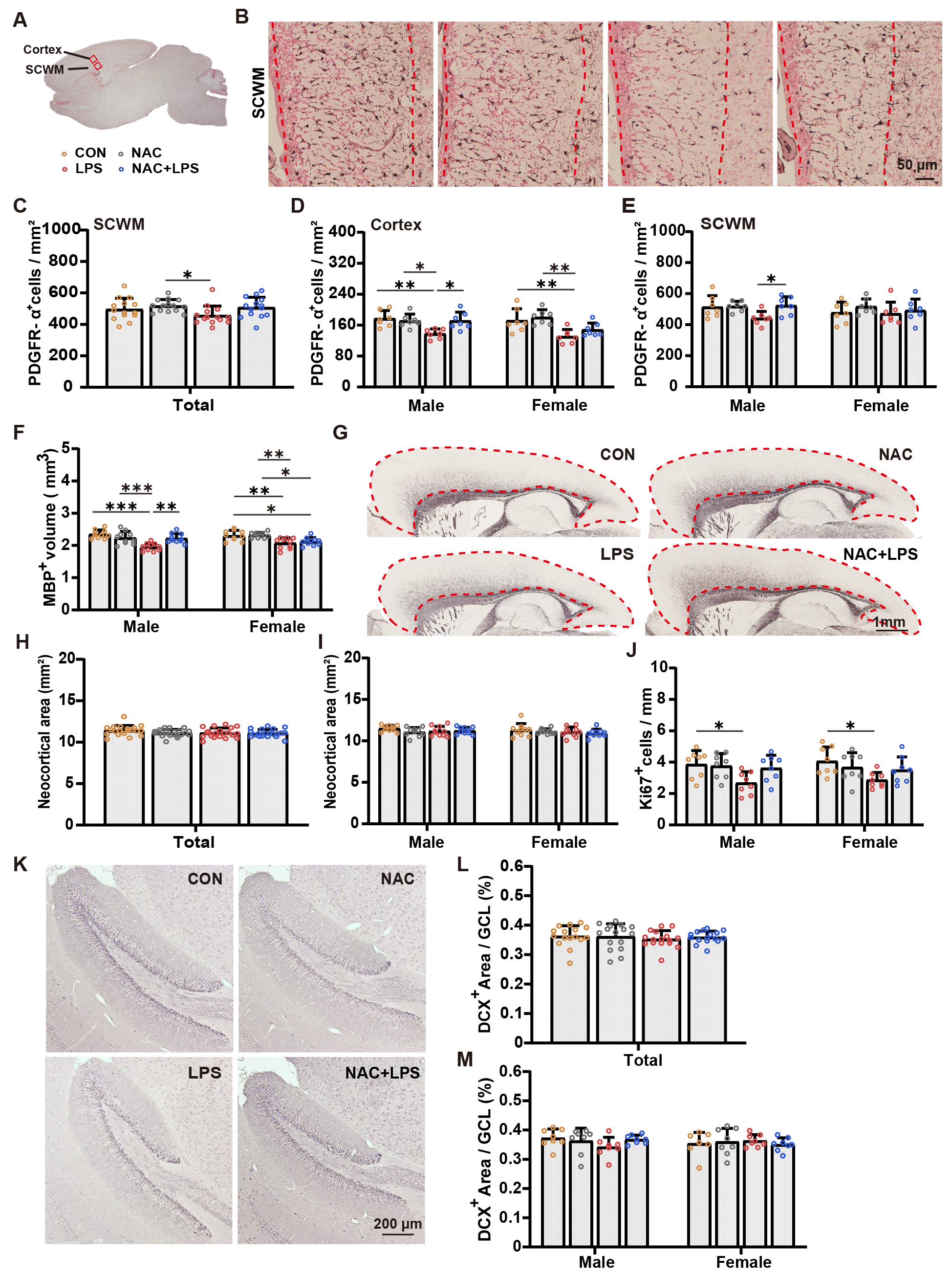


**Supplementary Figure S8. NAC effects on oligodendrocyte lineage markers, myelination, cortical tissue preservation, and neurodevelopmental alterations following neonatal sepsis.** (A) Schematic illustrating the regions used for quantification. (B) Representative immunohistochemical images of platelet-derived growth factor receptor-α (PDGFR-α) staining in sagittal sections of subcortical white matter (SCWM). The red outline indicates the SCWM region. (C) Quantification of PDGFR-α-positive cells in SCWM at 24 h after LPS exposure in the four experimental groups (n = 14 per group; 7 males and 7 females). (D–E) Quantification of PDGFR-α-positive cells in the cerebral cortex and SCWM at 24 h after LPS exposure in male and female pups across the four groups (n = 7 per group per sex). (F) MBP-positive volume in the subcortical white matter (SCWM) at postnatal day 21 (PND21) after LPS exposure in male and female pups across the four experimental groups (n = 10 per group per sex). (G) Representative MBP-stained sagittal sections used for quantification of neocortical area at PND21; the red outline indicates the analyzed region. (H–I) Quantification of neocortical area at PND21 after LPS exposure in the four experimental groups (n = 20 per group; 10 males and 10 females), including sex-stratified analysis (n = 10 per group per sex). (J) Ki-67-positive cells in the dentate gyrus (DG) at PND21 after LPS exposure in male and female pups across the four groups (n = 8 per group per sex). (K) Representative immunohistochemical images of doublecortin (DCX) staining in sagittal sections of the DG. (L–M) Ratio of DCX-positive immunoreactive area to granule cell layer (GCL) area in the DG at PND21 after LPS exposure in the four experimental groups (n = 16 per group; 8 males and 8 females), including sex-stratified analysis (n = 8 per group per sex). Data are presented as mean ± SD. Effects of treatment group, sex, and their interaction were evaluated by two-way ANOVA. Between-group comparisons were performed using one-way ANOVA followed by Tukey’s or Games–Howell post hoc tests, or by Kruskal–Wallis analysis with Dunn’s post hoc correction when parametric assumptions were not met. **P* < 0.05, ***P* < 0.01, ****P* < 0.001.
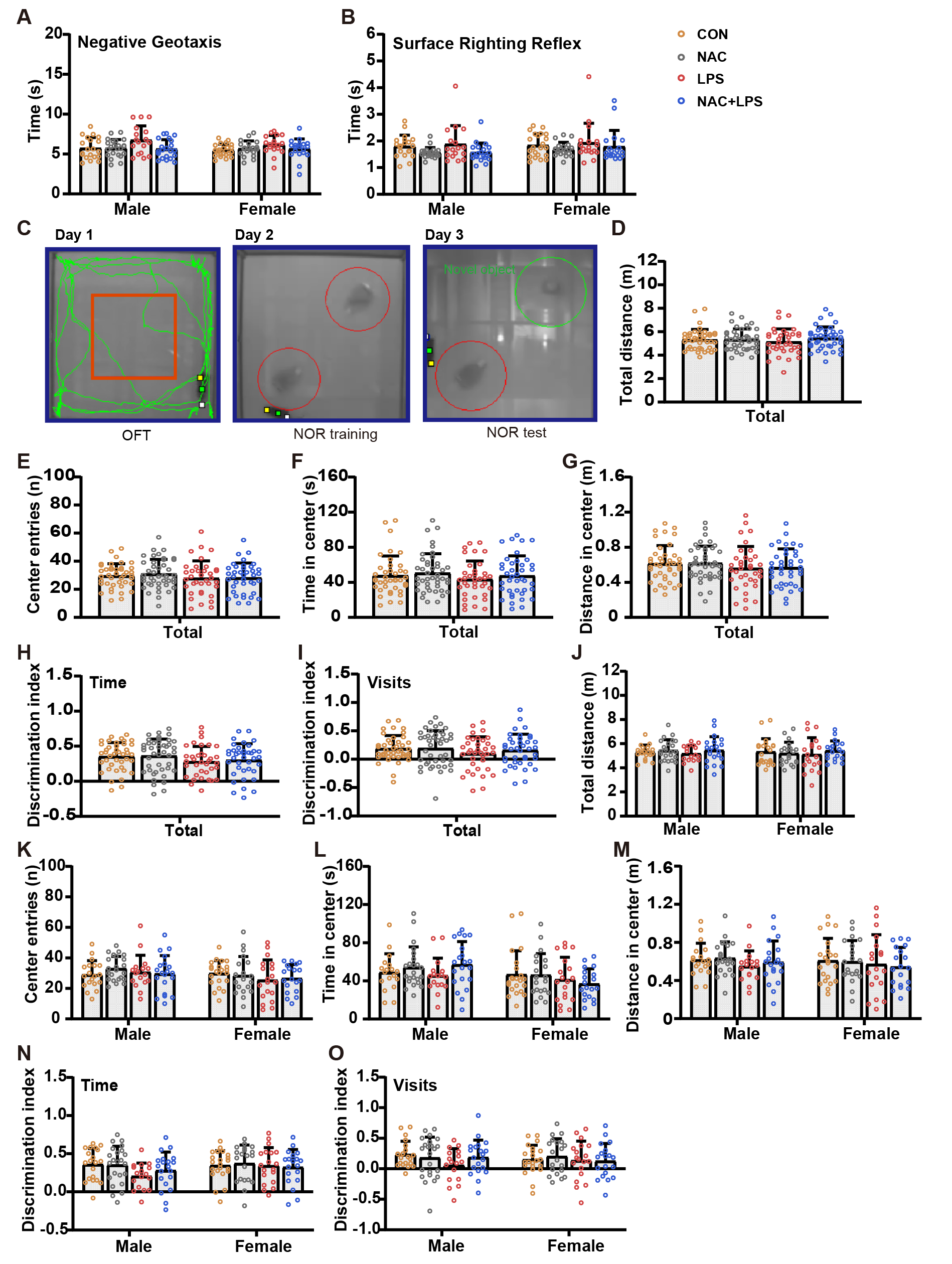


**Supplementary Figure S9. Effects of NAC on early and late behavioral outcomes after neonatal LPS exposure.** (A–B) Latency to complete the negative geotaxis and surface righting reflex tests at postnatal day 8 (PND8) after LPS exposure in male and female pups across the four experimental groups (n = 17–23 per group per sex). (C) Behavioral testing paradigm: open field test (OFT; day 1), novel object recognition (NOR) training (day 2), and NOR test (day 3). (D–G) OFT parameters, including total distance traveled, entries into the center zone, time spent in the center zone, and distance traveled in the center zone, at PND60 after LPS exposure in the four groups (n = 37–44 per group; 17–23 males and 19–22 females). (H–I) NOR parameters, including discrimination index based on time spent exploring each object and discrimination index based on the number of visits, at PND60 after LPS exposure in the four groups (n = 37–44 per group; 17–23 males and 19–22 females). (J–M) OFT parameters at PND60 after LPS exposure in male and female pups across the four groups (n = 17–23 per group per sex). (N–O) NOR parameters at PND60 after LPS exposure in male and female pups across the four groups (n = 17–23 per group per sex). Data are presented as mean ± SD. Effects of treatment group, sex, and their interaction were evaluated by two-way ANOVA. Between-group comparisons were performed using one-way ANOVA followed by Tukey’s post hoc tests, or by Kruskal–Wallis analysis with Dunn’s post hoc correction when parametric assumptions were not met.
