# Supplementary material for: N-Acetylcysteine protects the developing brain in neonatal sepsis-like inflammation via a redox–neurovascular pathway
Source: J Neuroinflammation. 2026 Jun 30;23:222. doi: 10.1186/s12974-026-03942-9 (PMC13317334; doi:10.1186/s12974-026-03942-9)

**Supplementary Material 2**

Original, unedited Western blot images used to generate the figures. Red rectangles indicate the bands shown in the figures. Bridge samples included in multiple blots for normalization are indicated.

**Figure 3F**

Male

NLPR 3


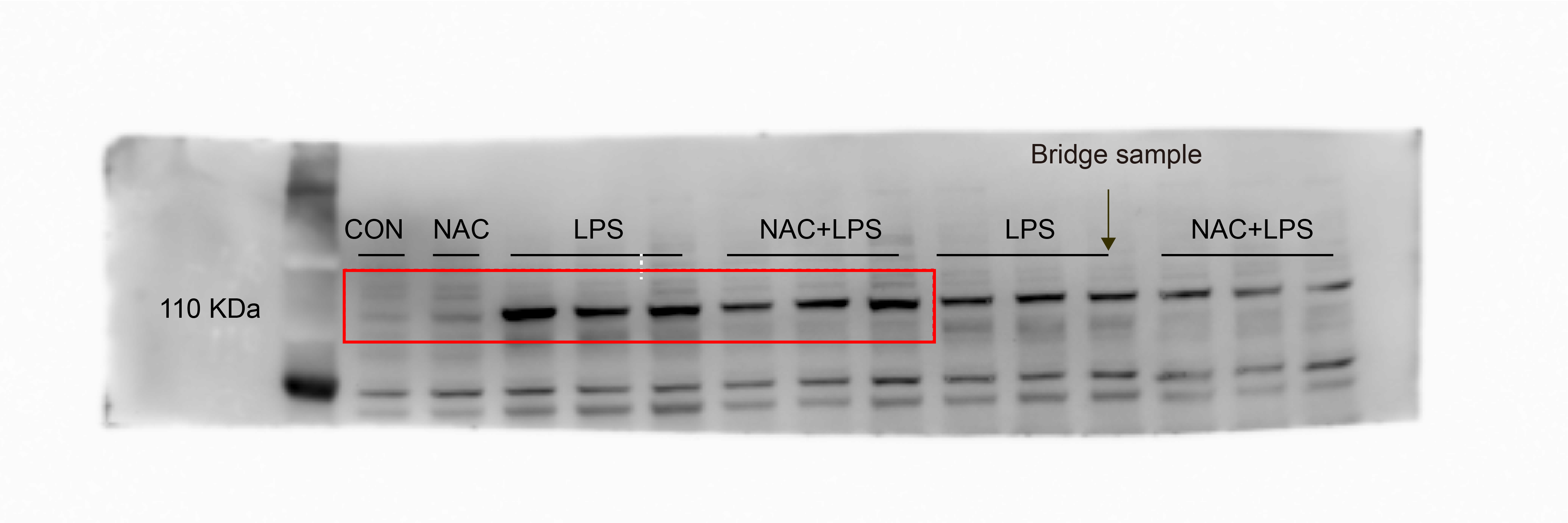

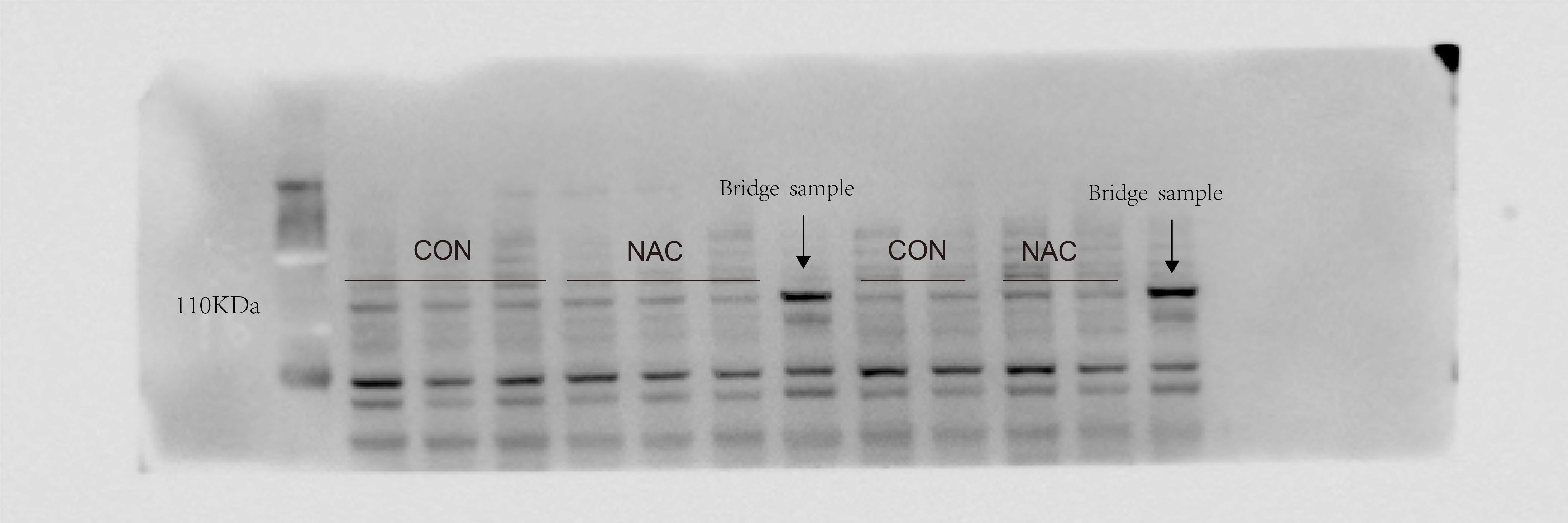


β-actin

NLPR 3


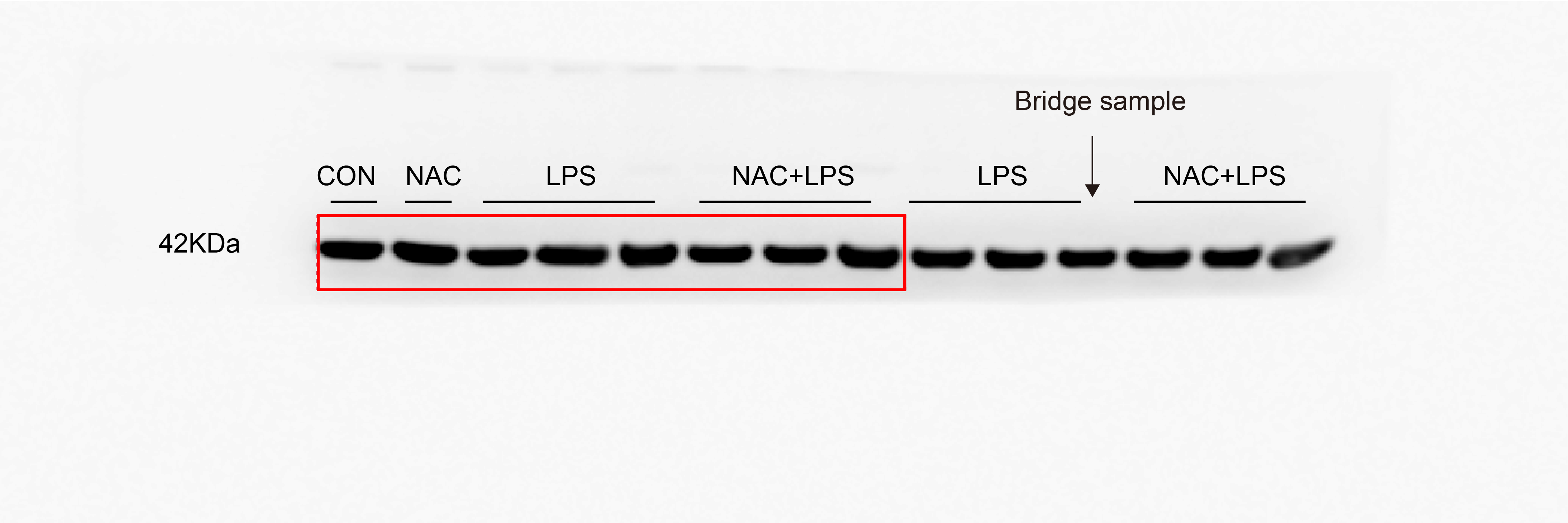

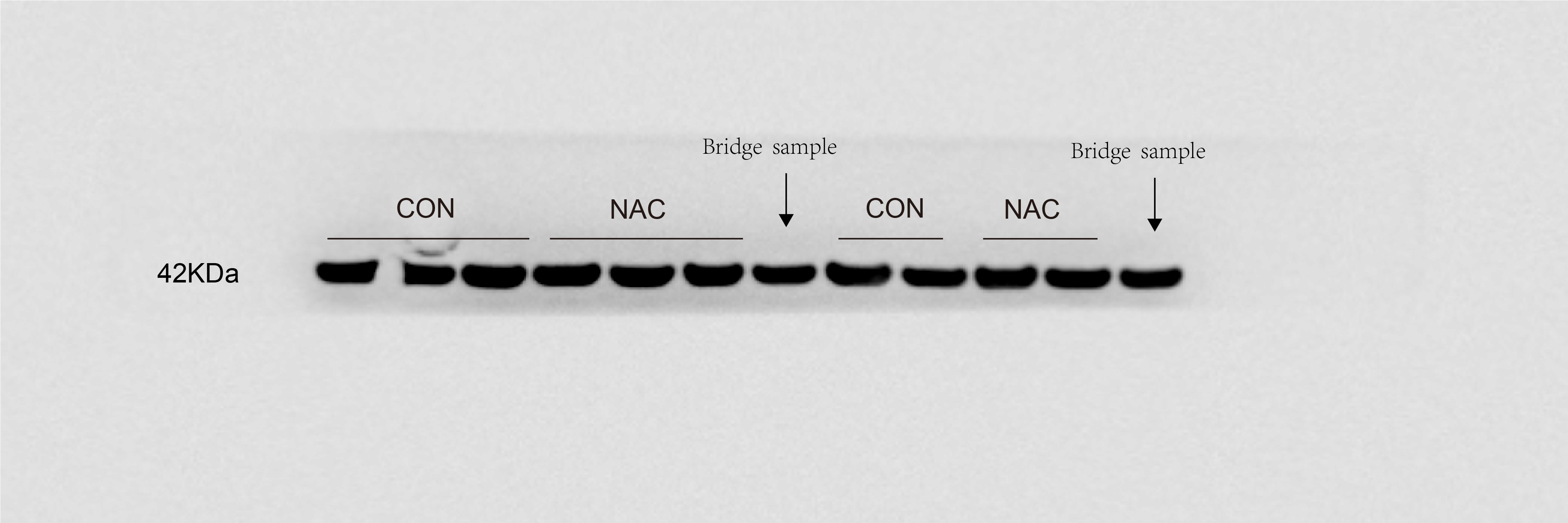


β-actin

Female


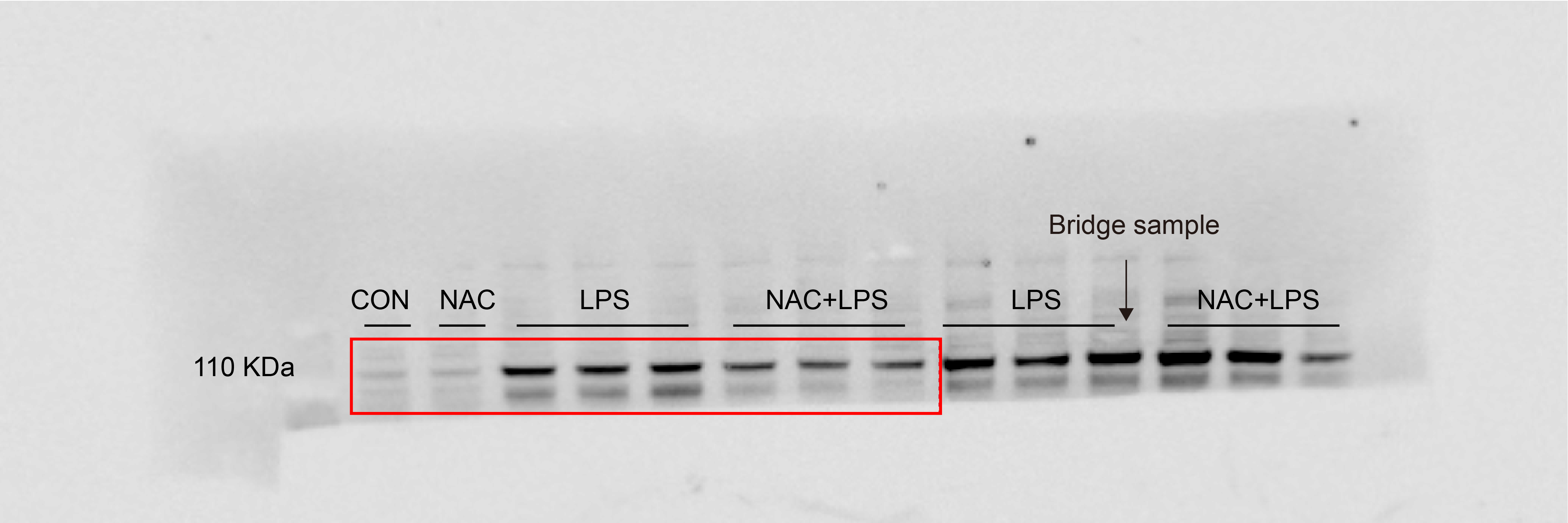

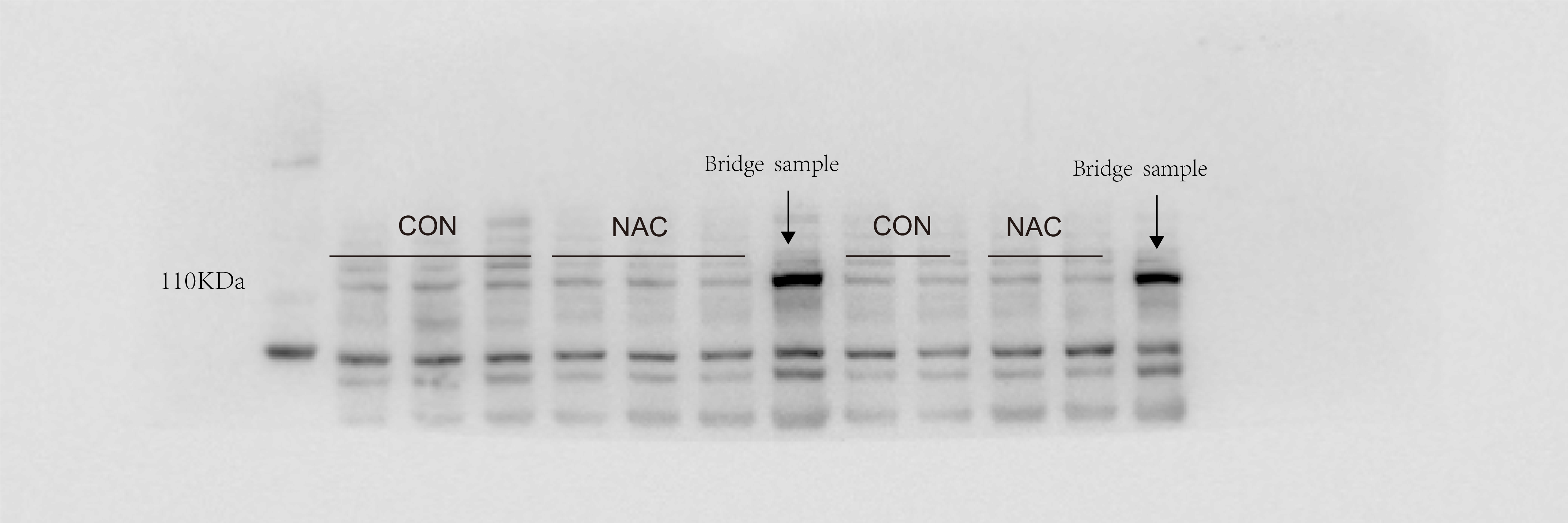


NLPR 3

NLPR 3


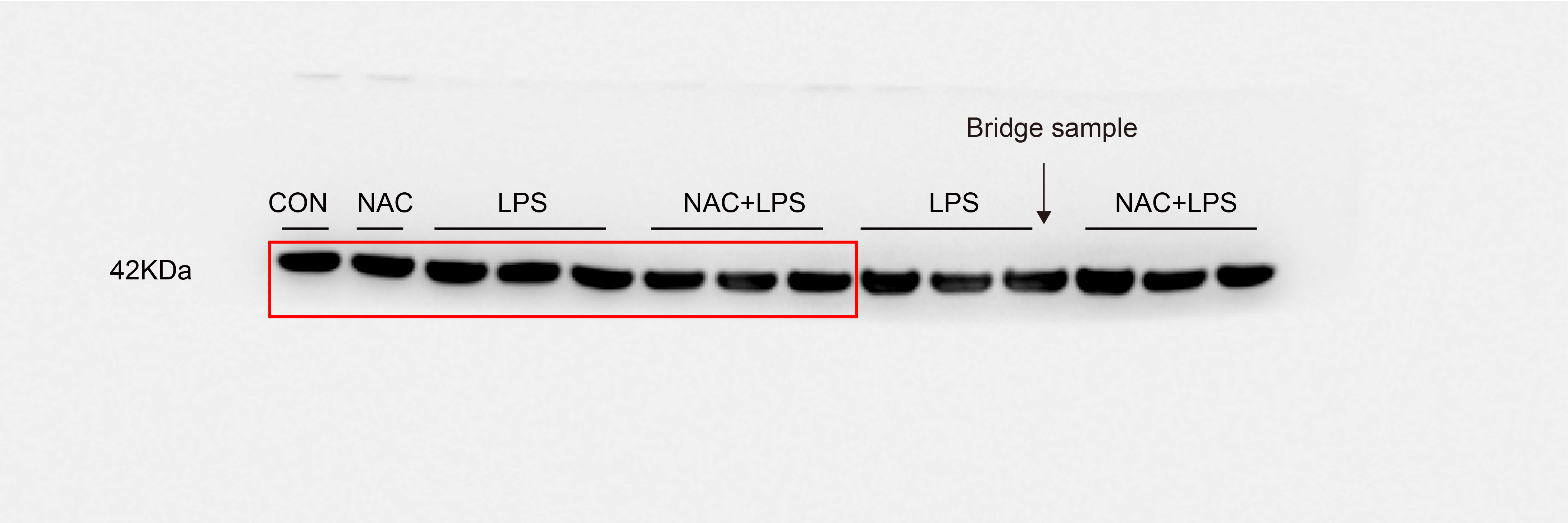

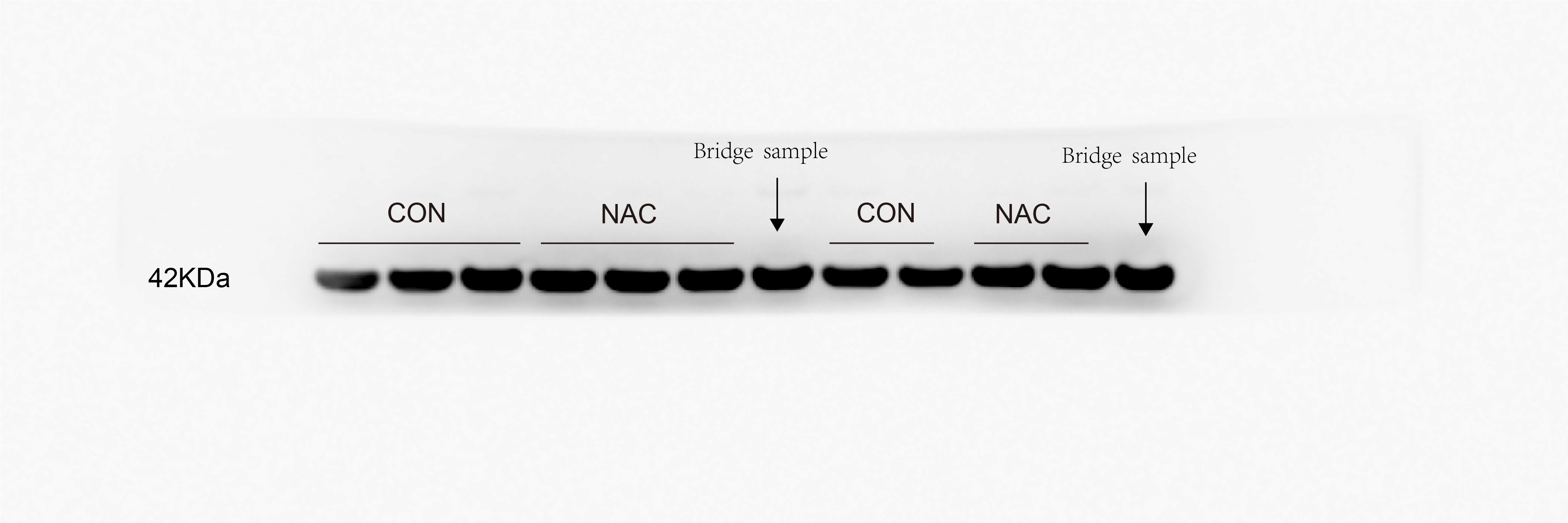


β-actin

β-actin

**Figure 4G**

Male


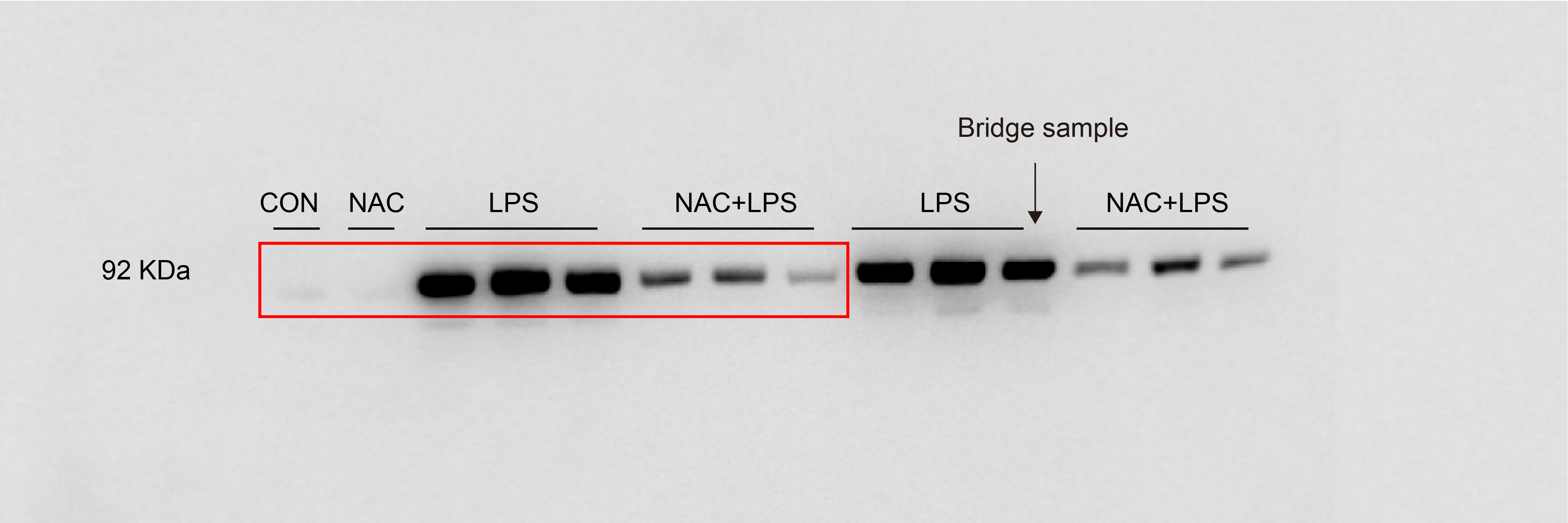

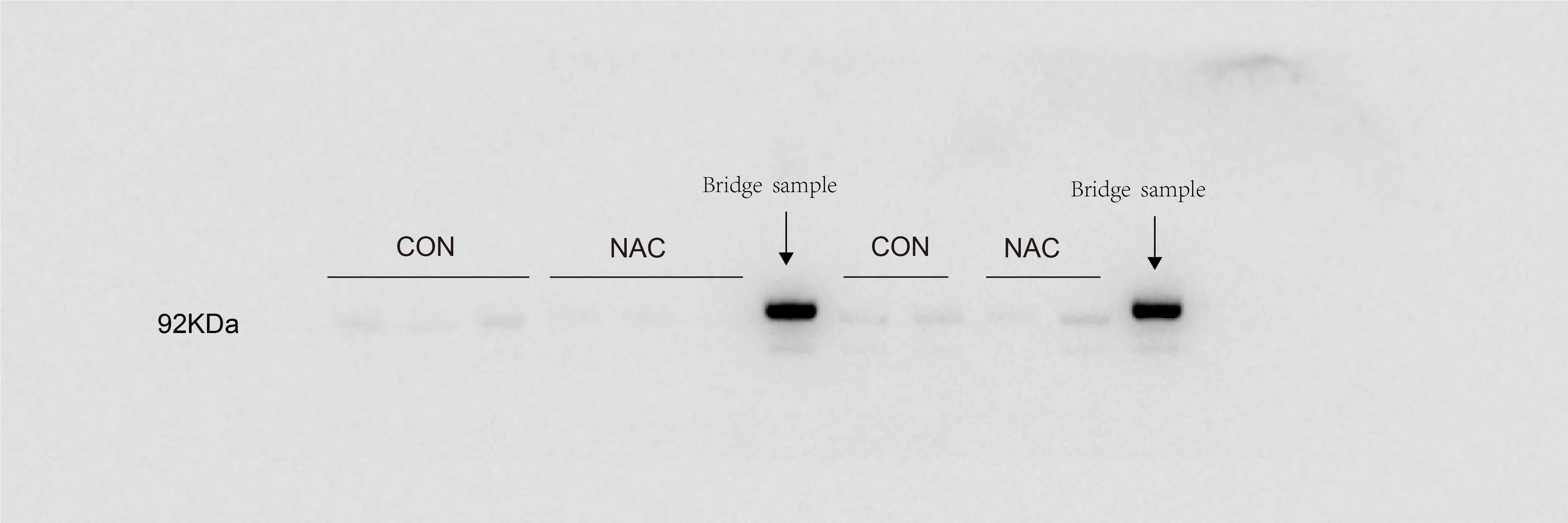


MMP-9

MMP-9


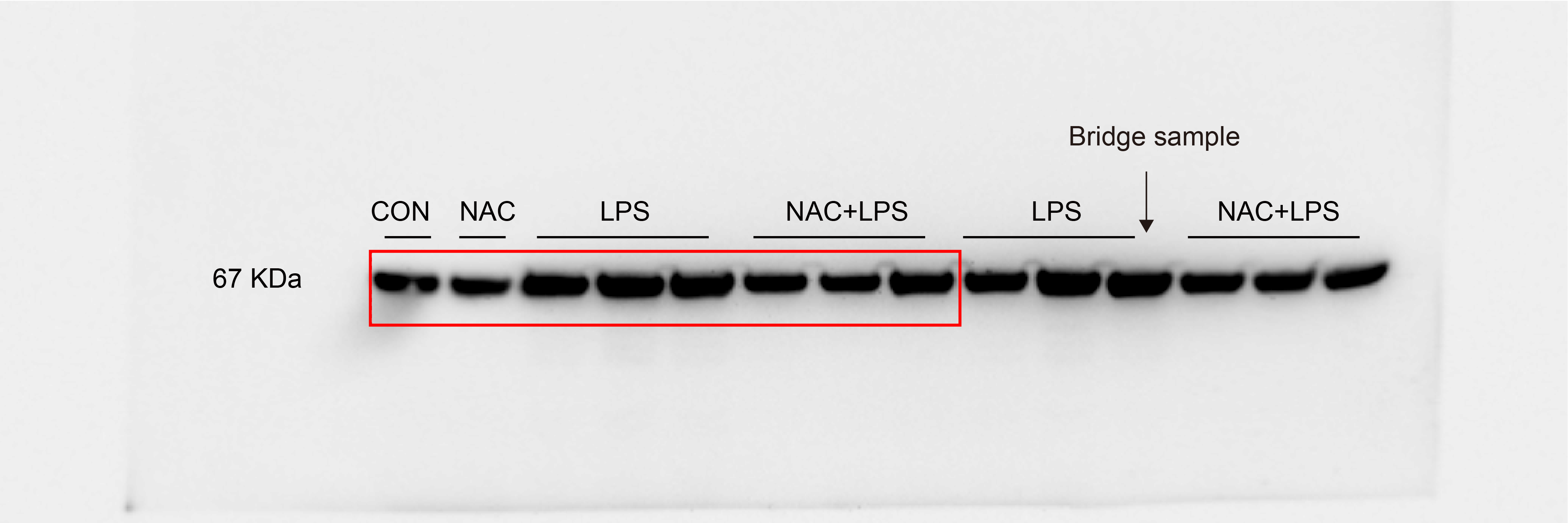

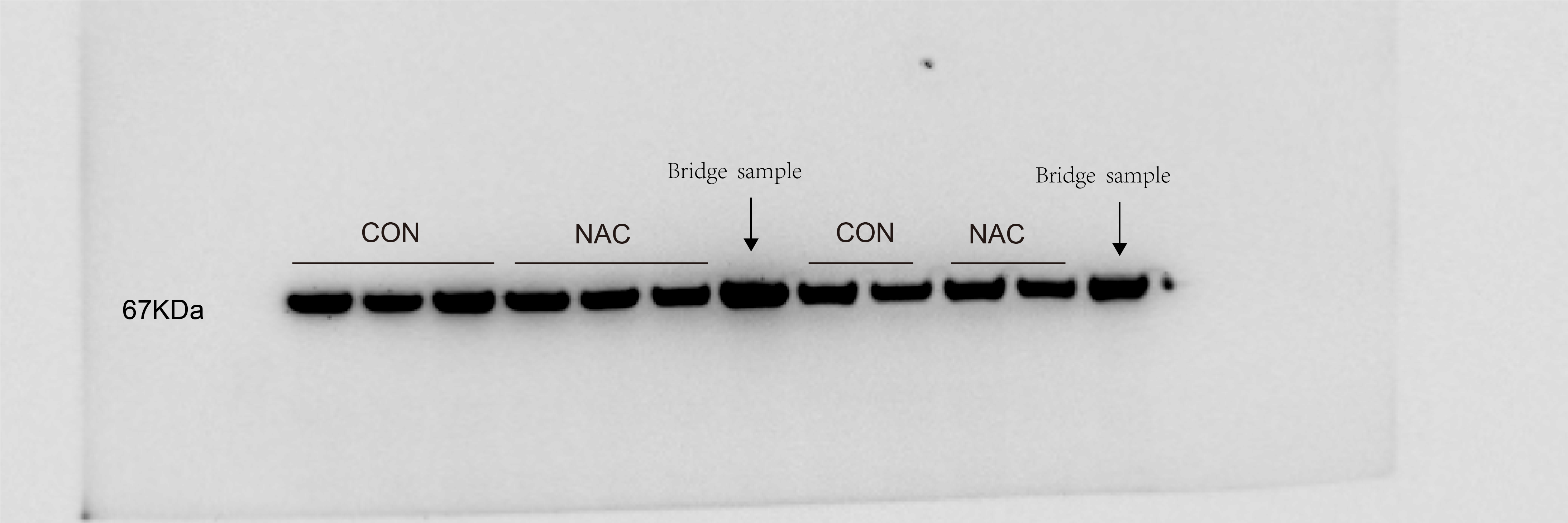


albumin

albumin


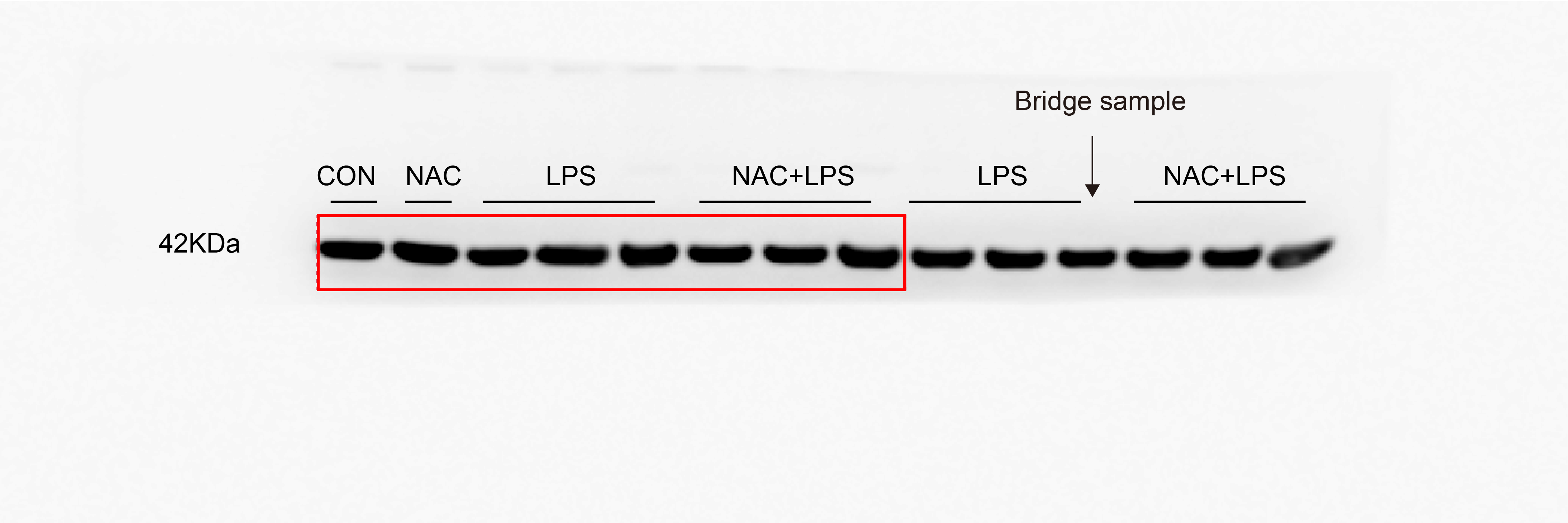

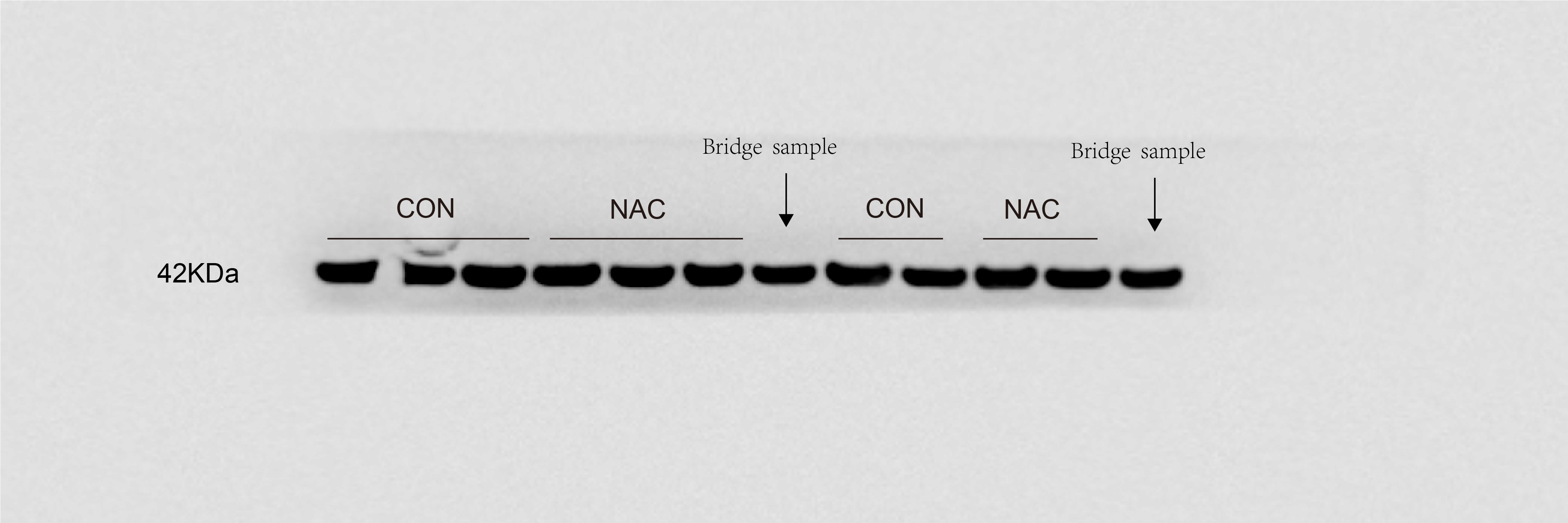


β-actin

β-actin

Female


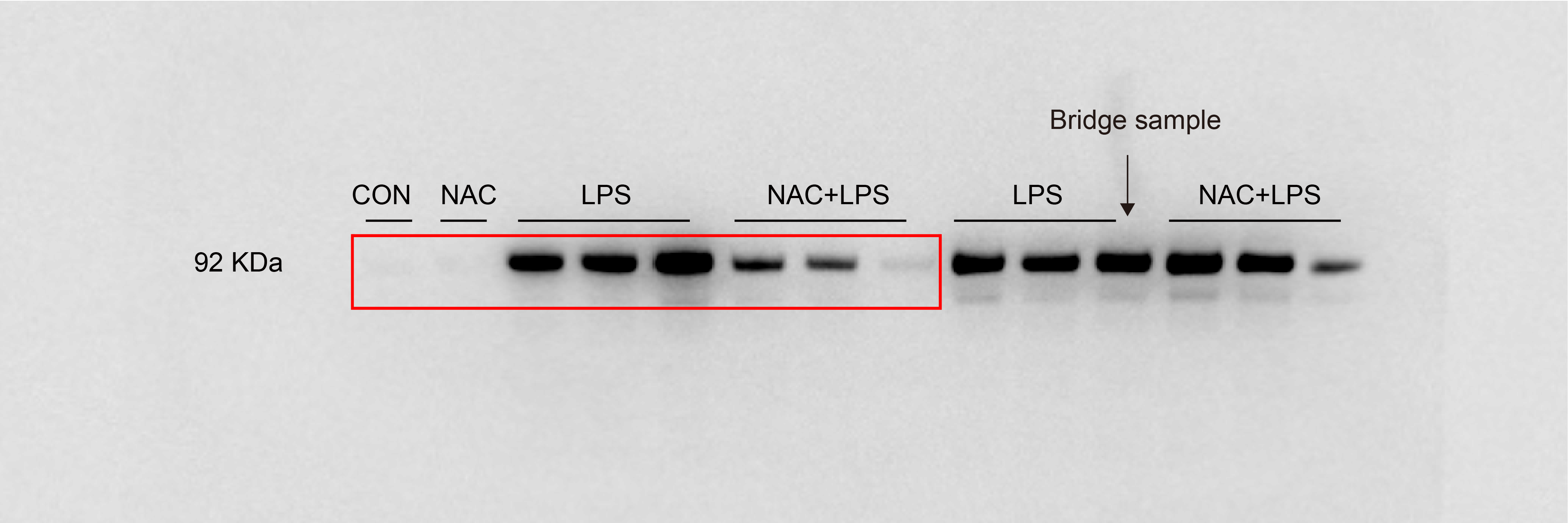

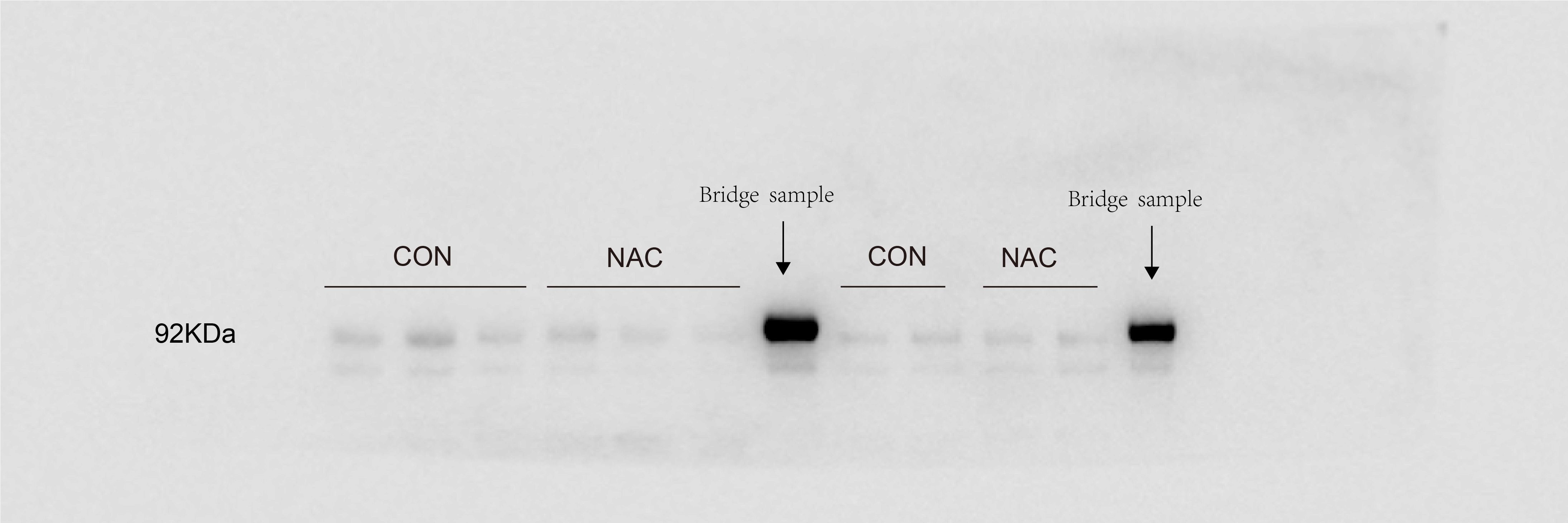


MMP-9

MMP-9


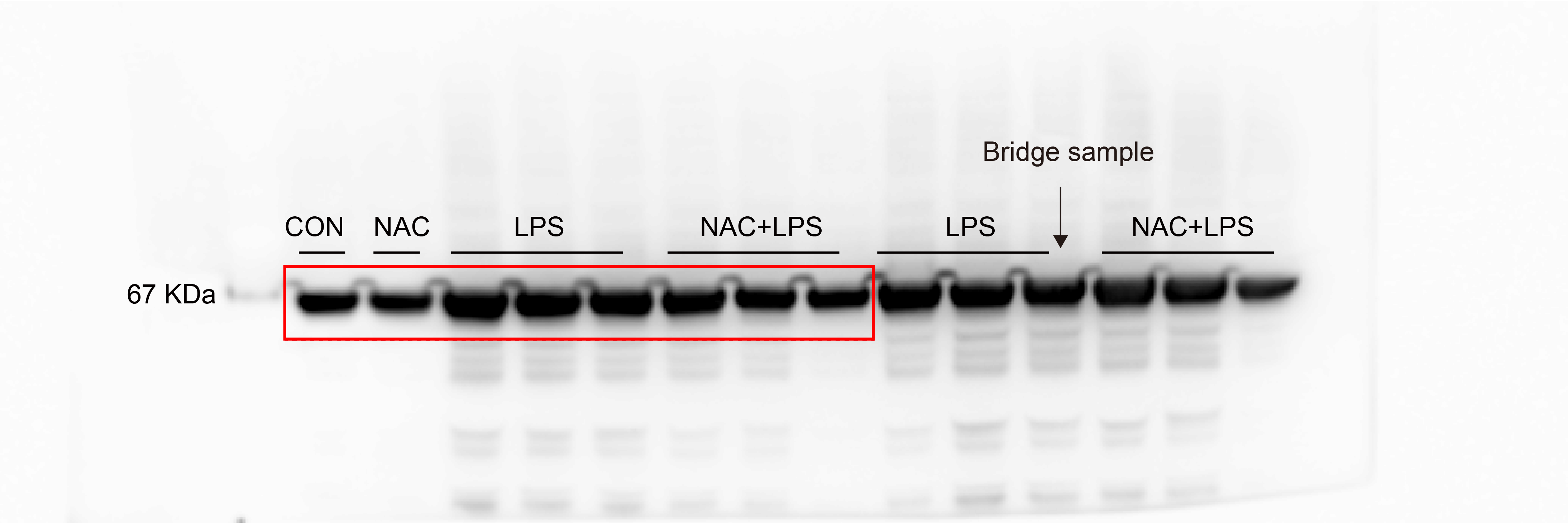

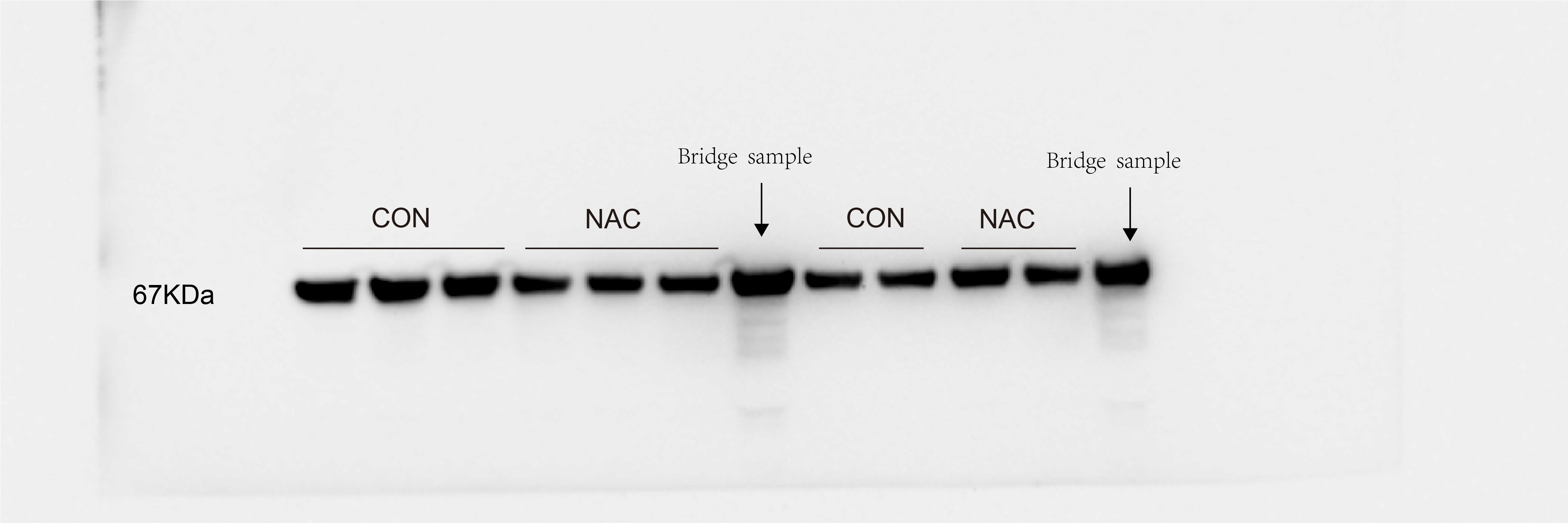


albumin

albumin


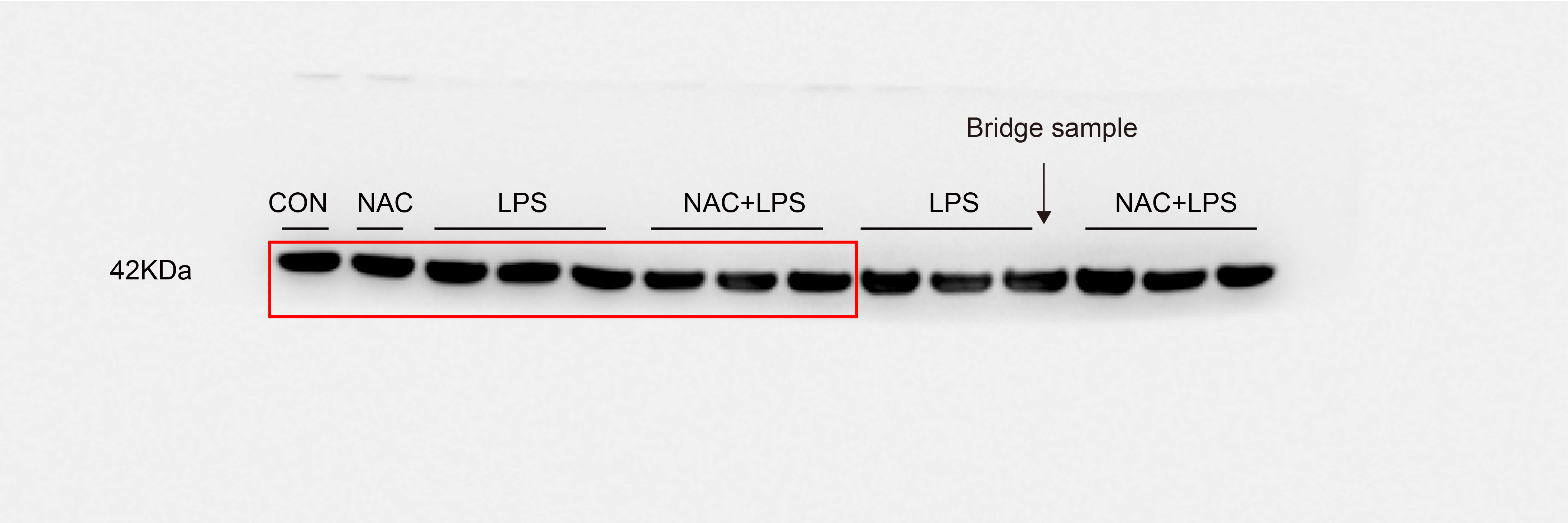

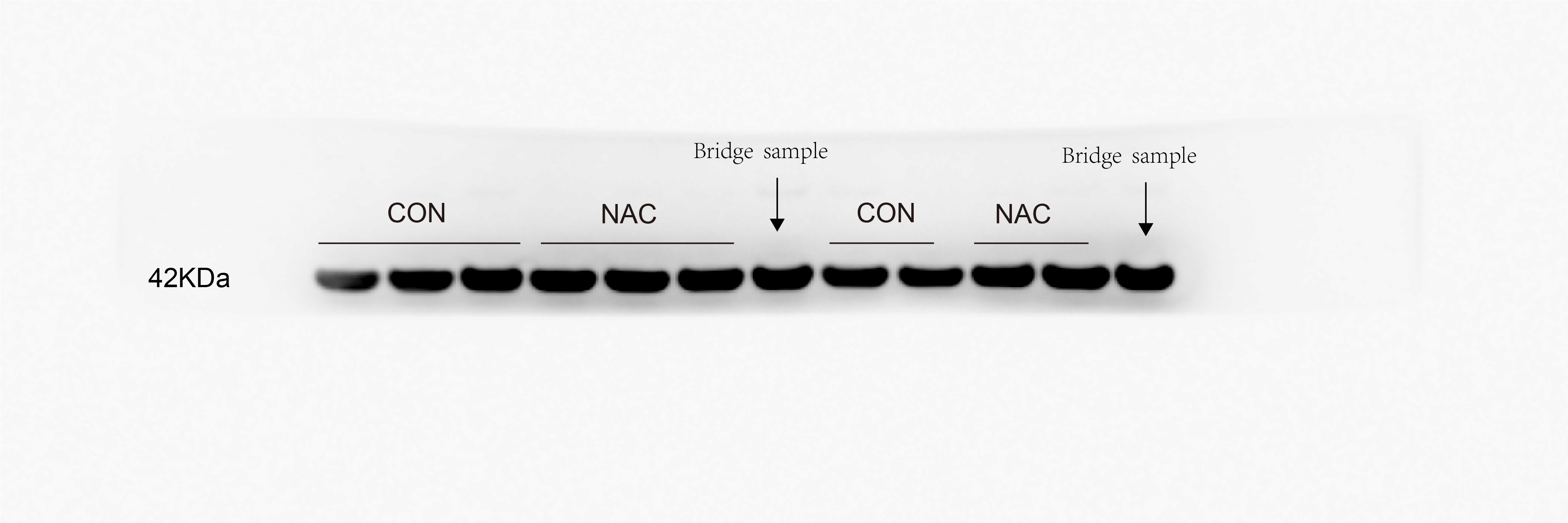


β-actin

β-actin

**Figure 5J**

Male

cleaved caspase-3

cleaved caspase-3


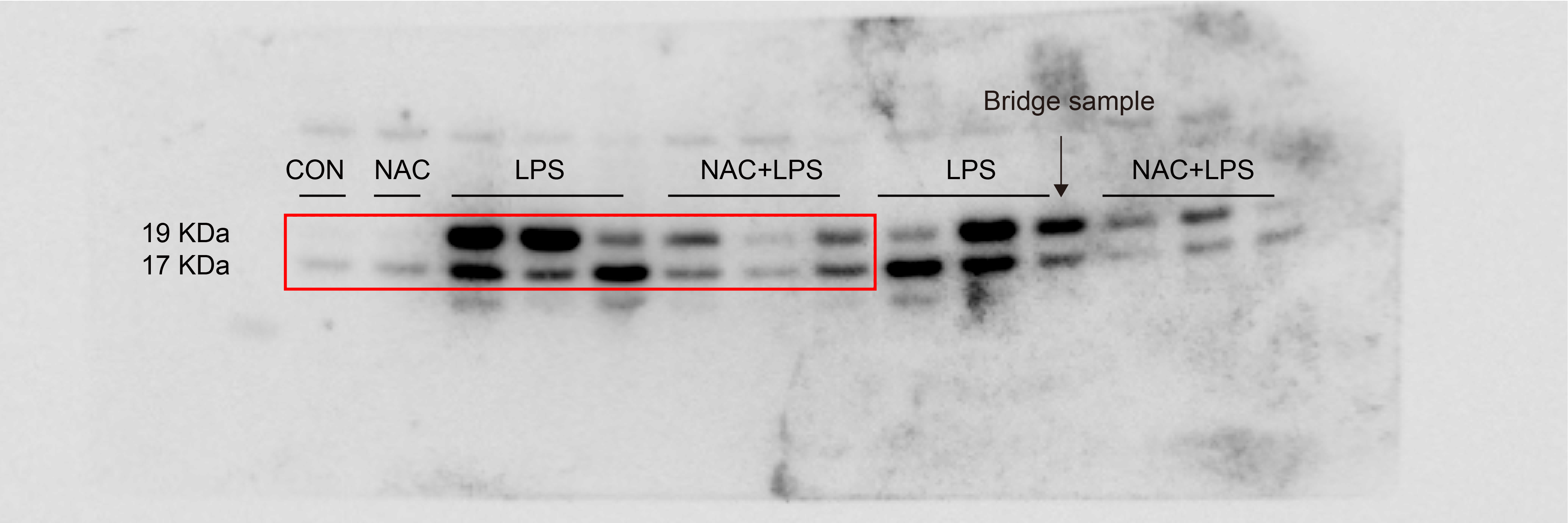

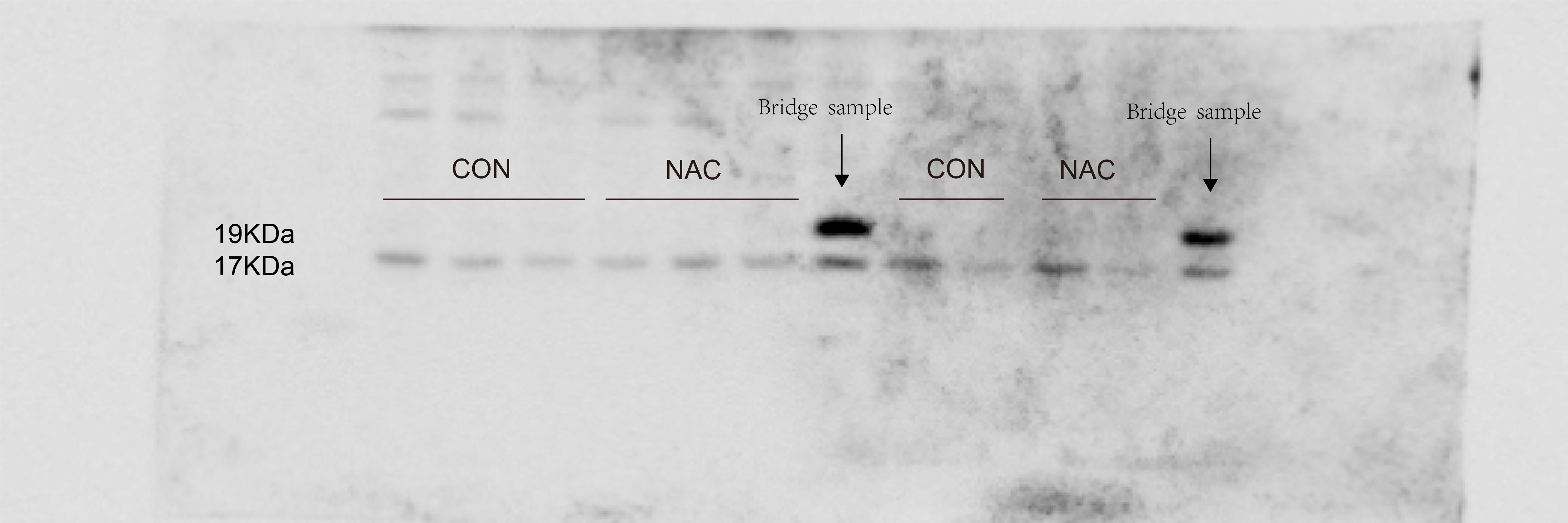


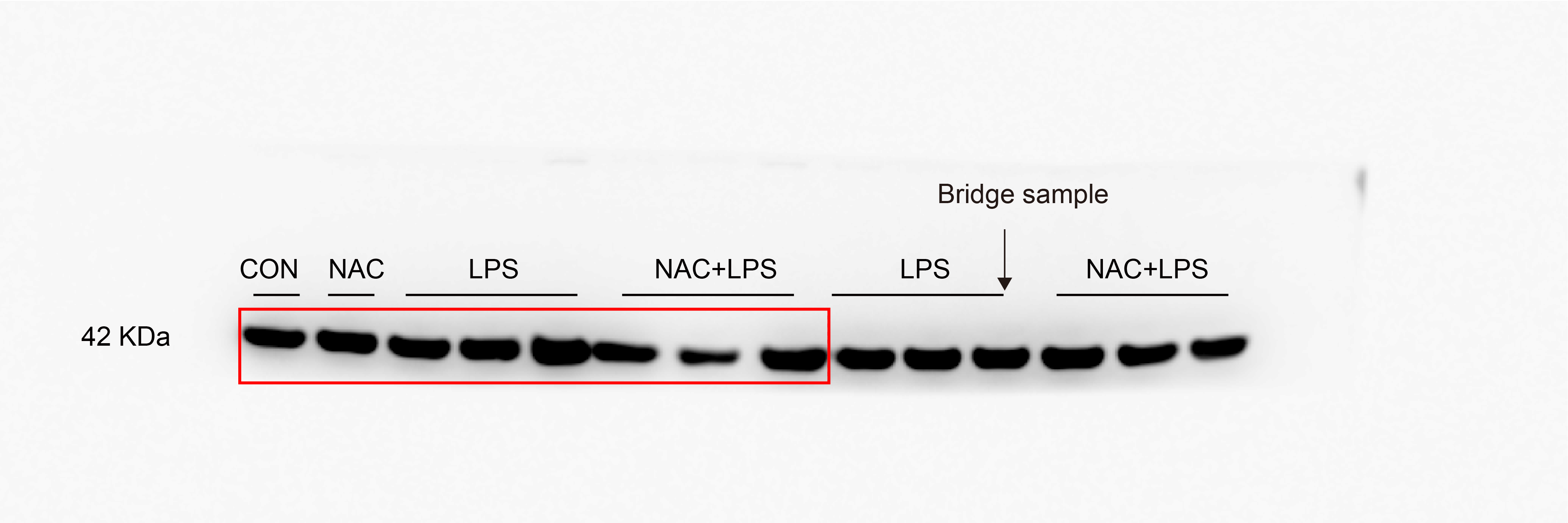

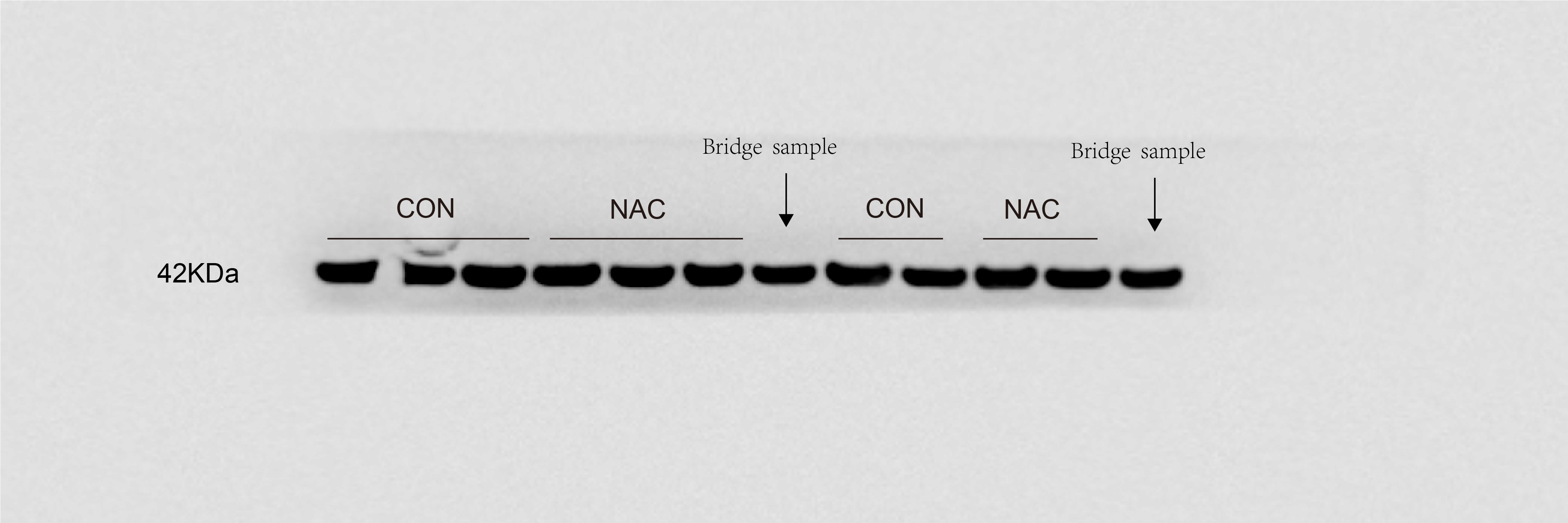


β-actin

β-actin

Female

cleaved caspase-3

cleaved caspase-3


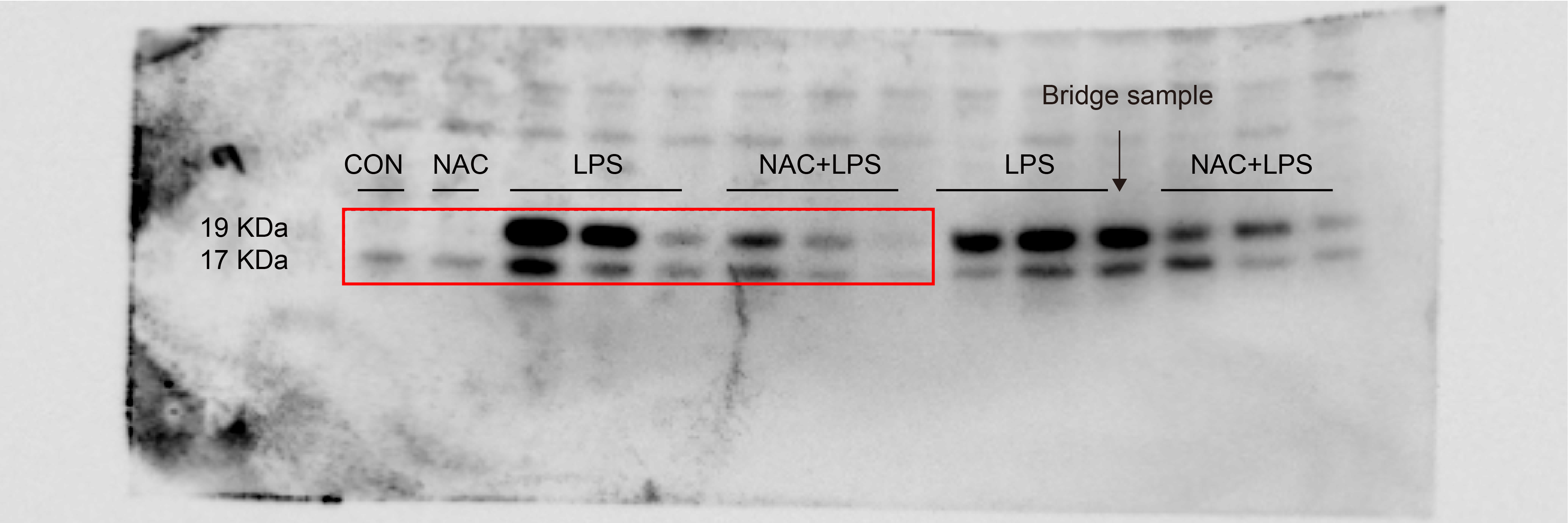

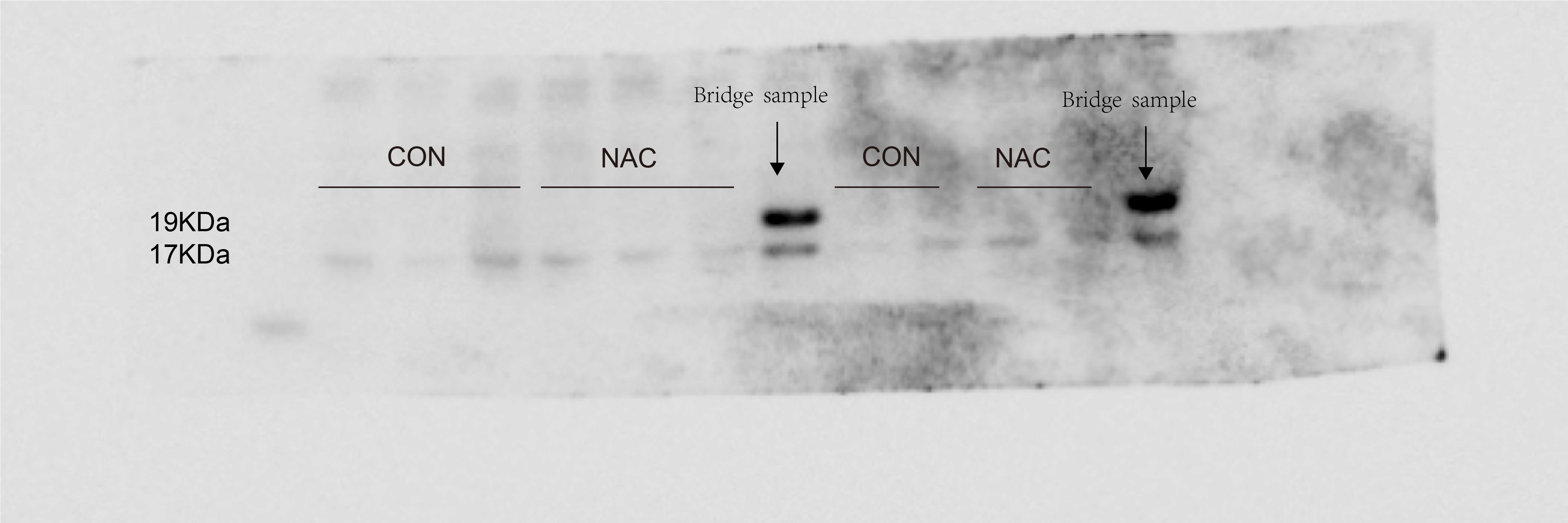


β-actin

β-actin


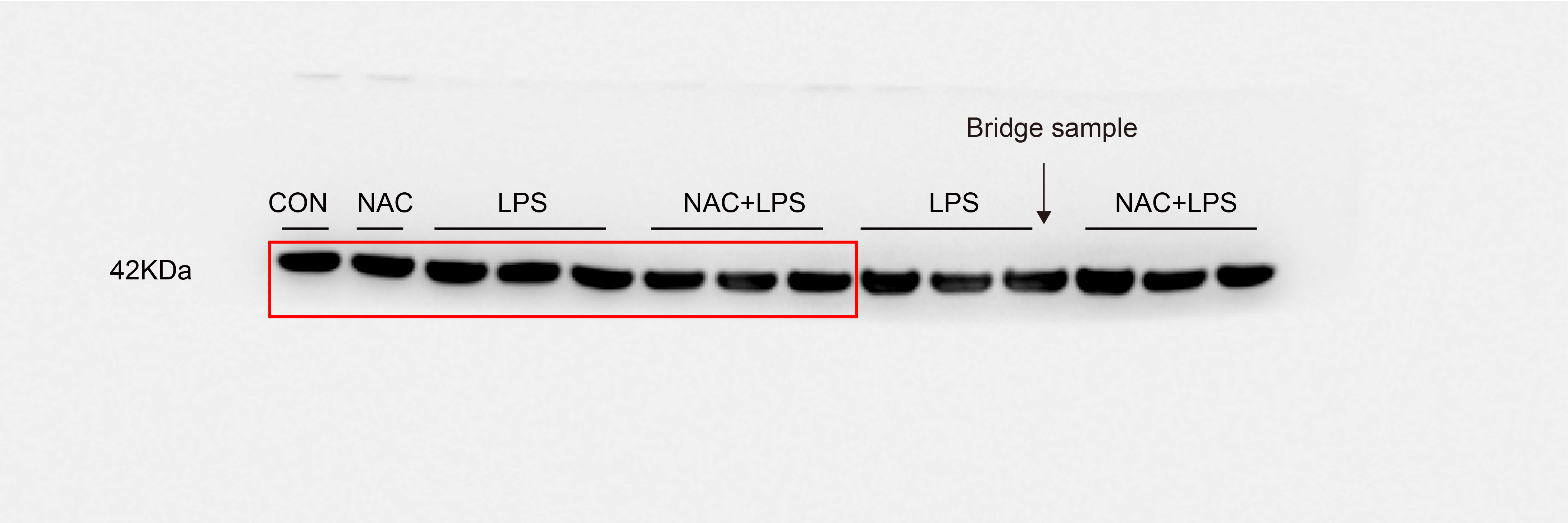

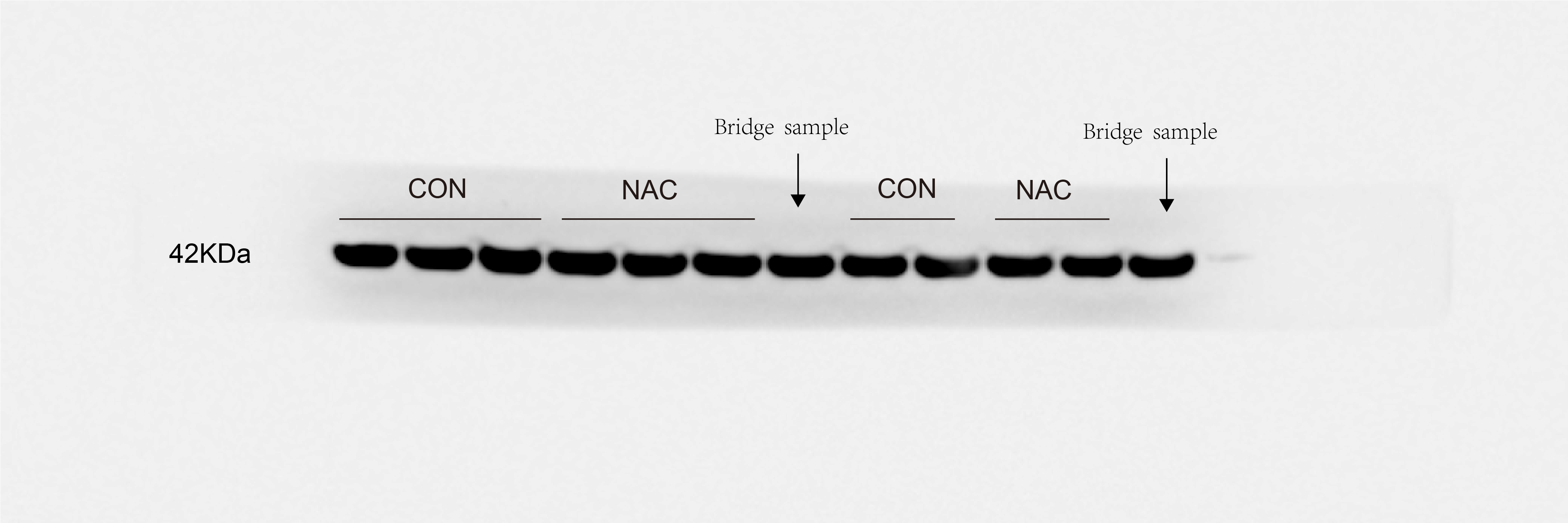


**Supplementary Figure S3E**

Male

SOD2

SOD2


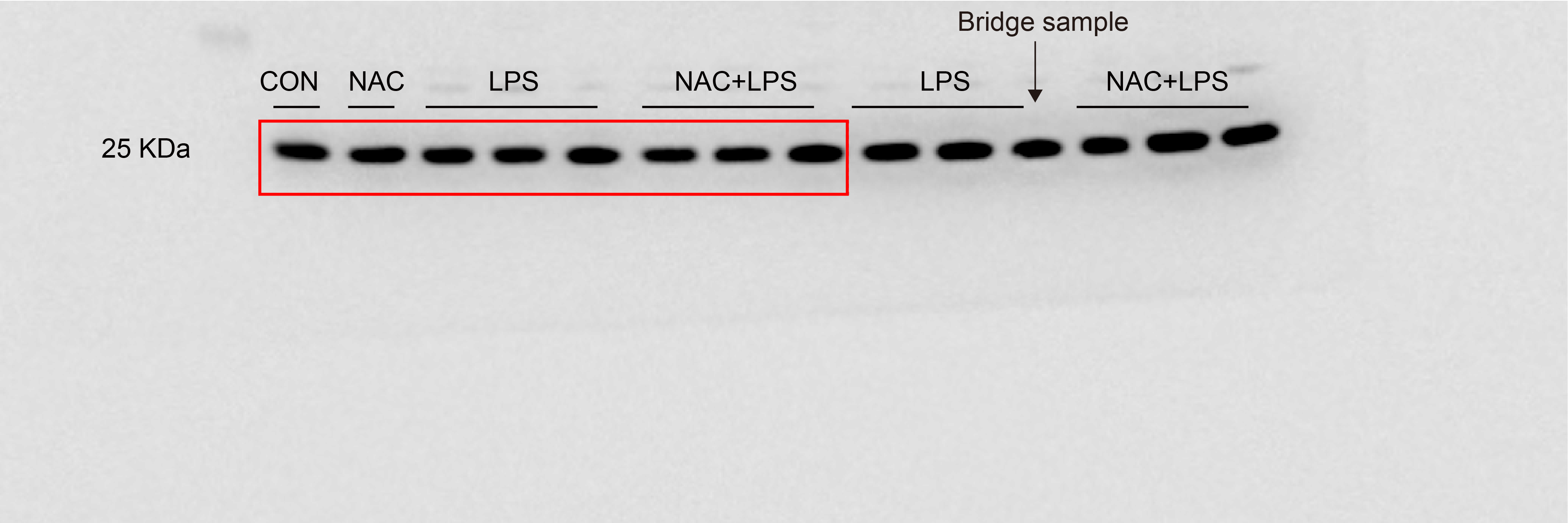

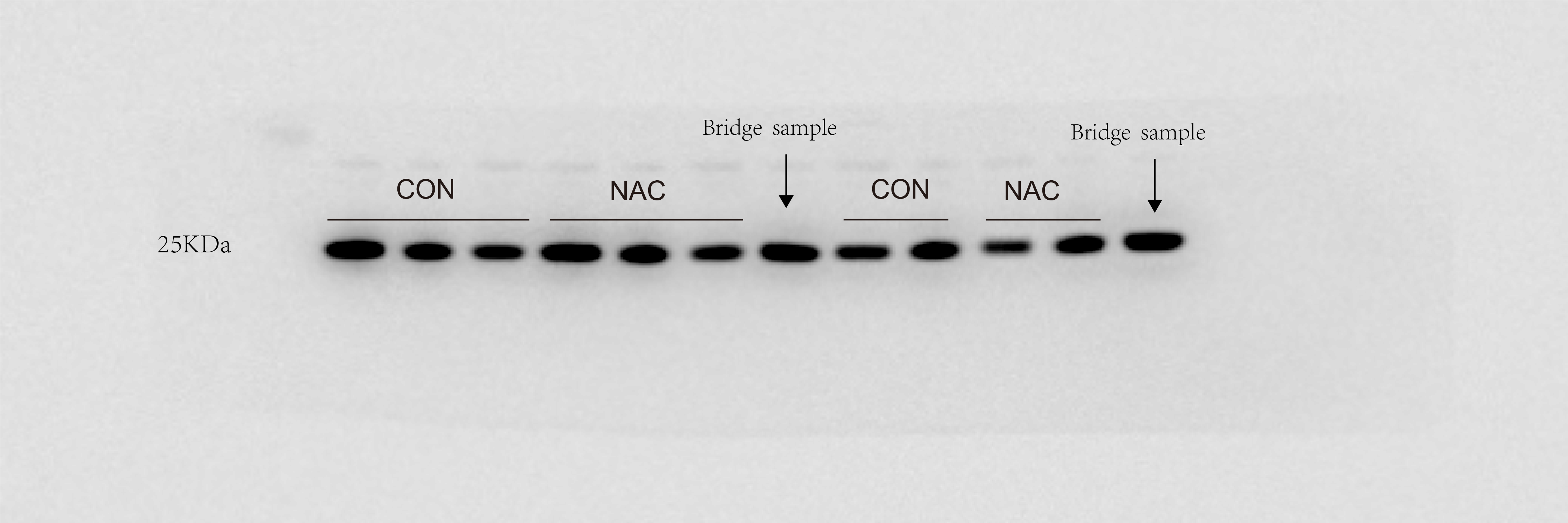


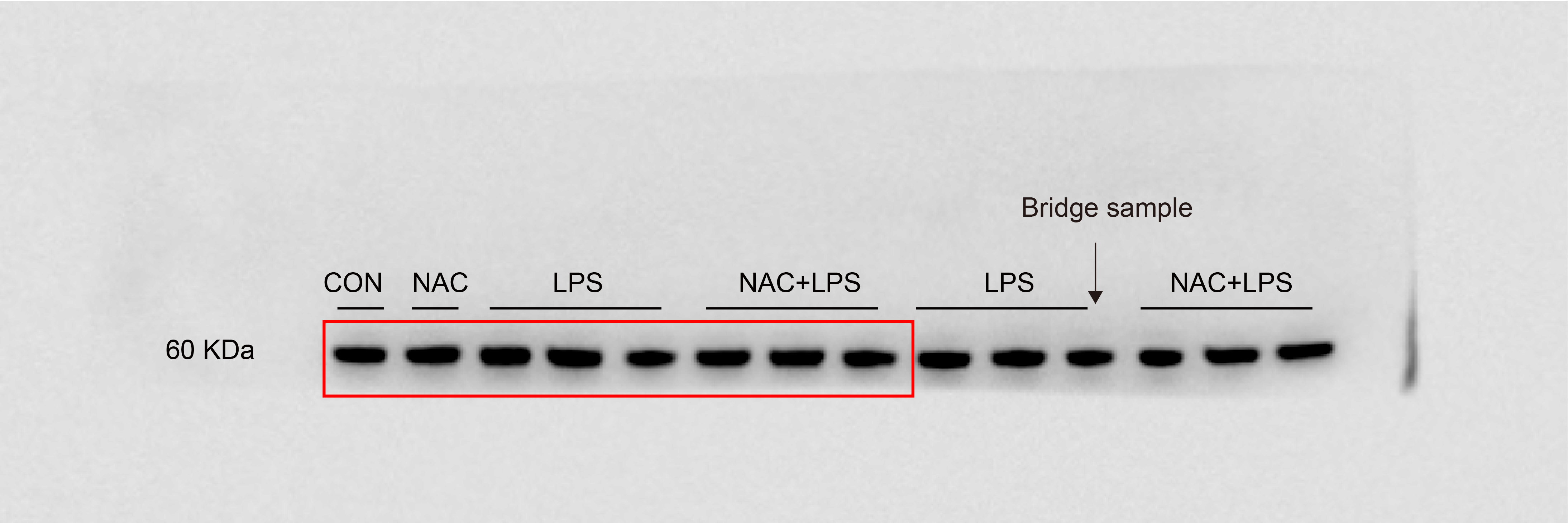

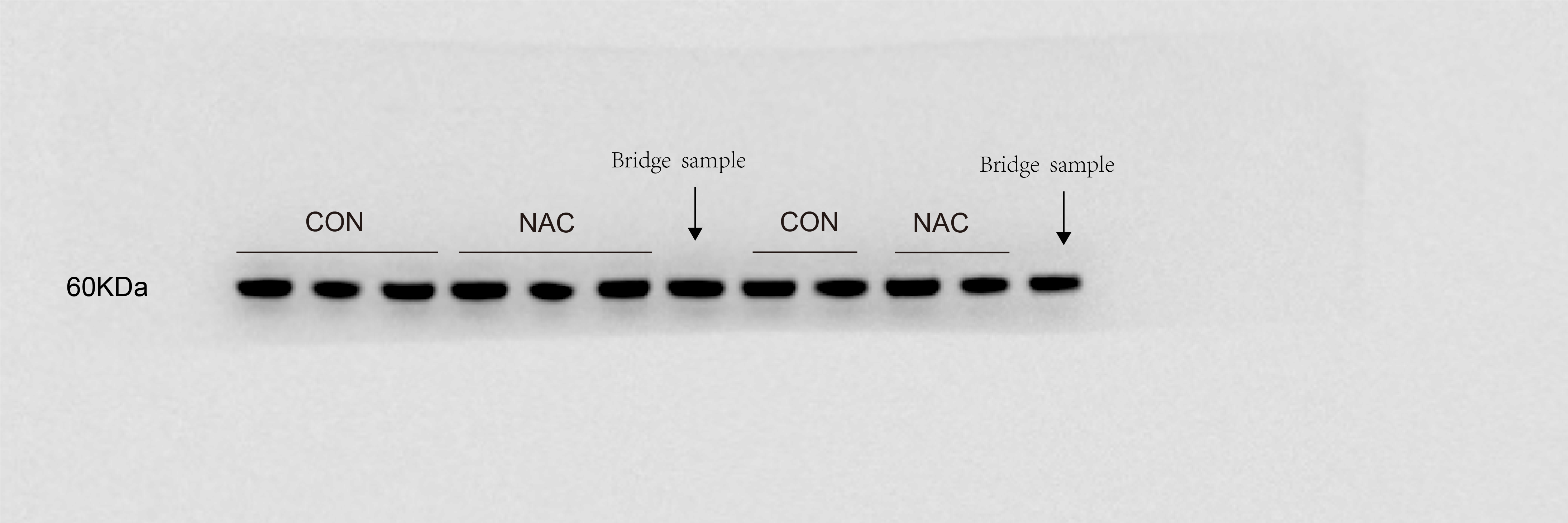


catalase

catalase


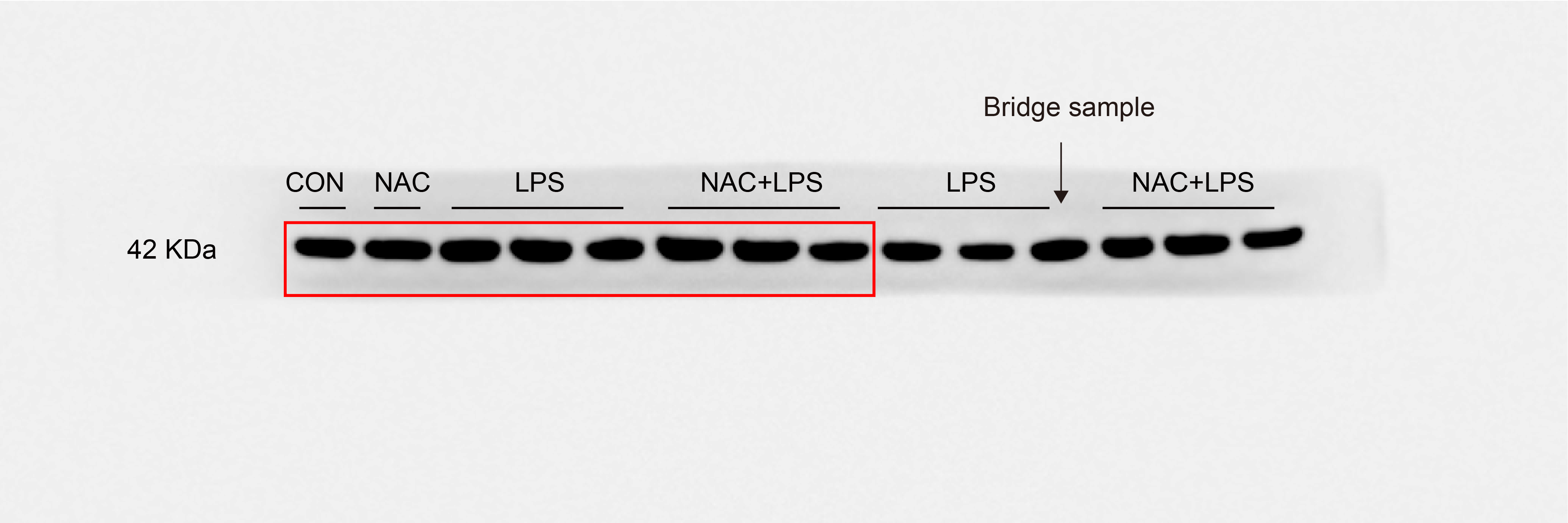

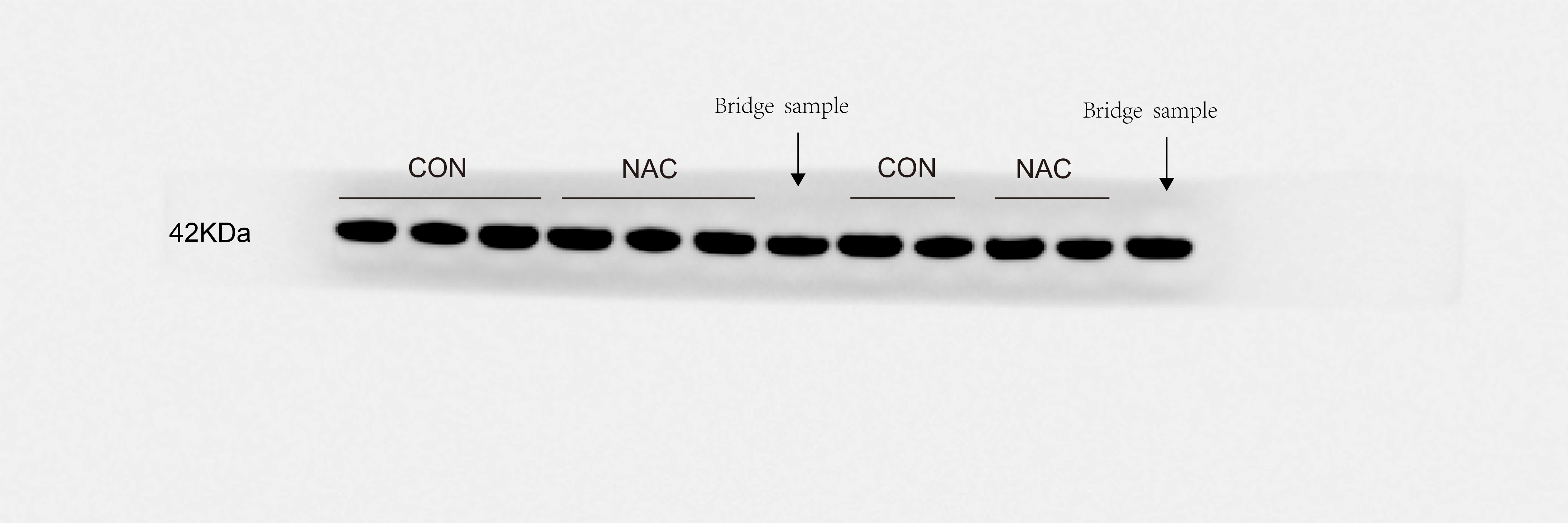


β-actin

β-actin

Female

SOD2

SOD2


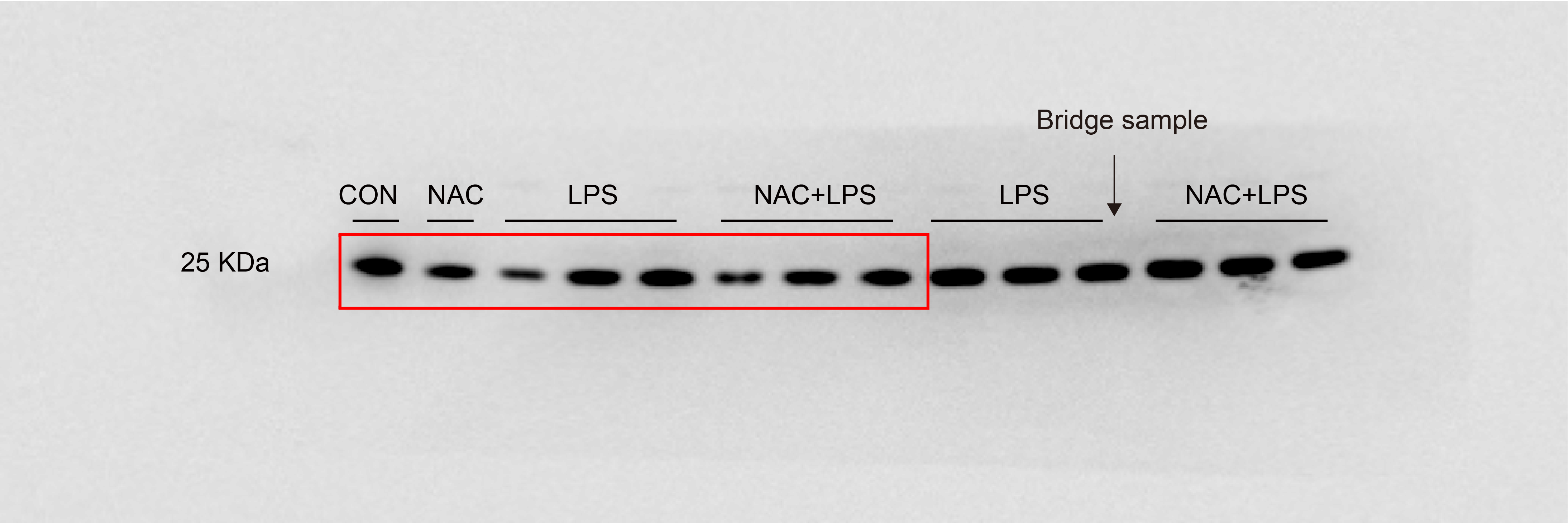

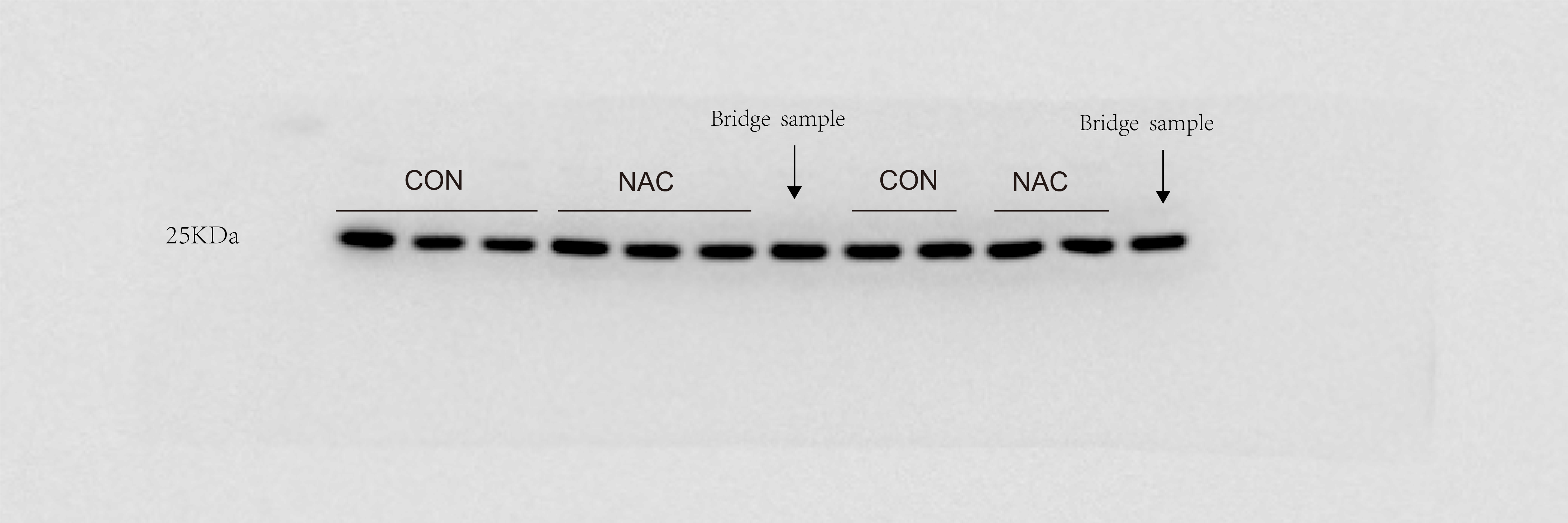


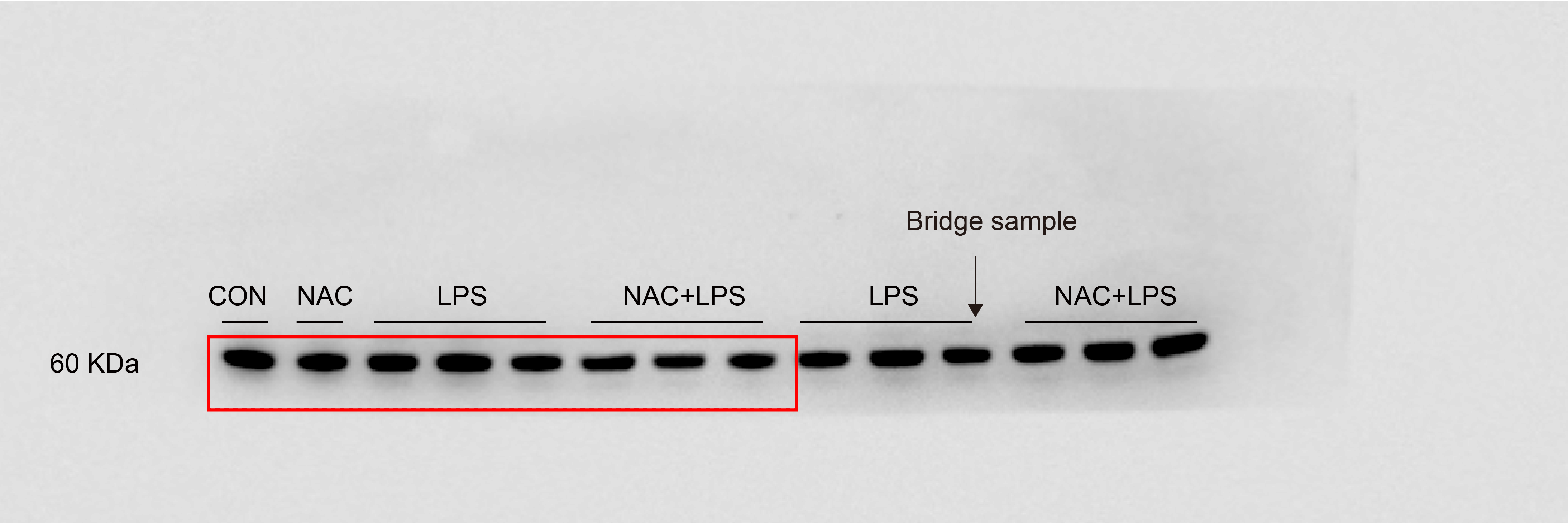

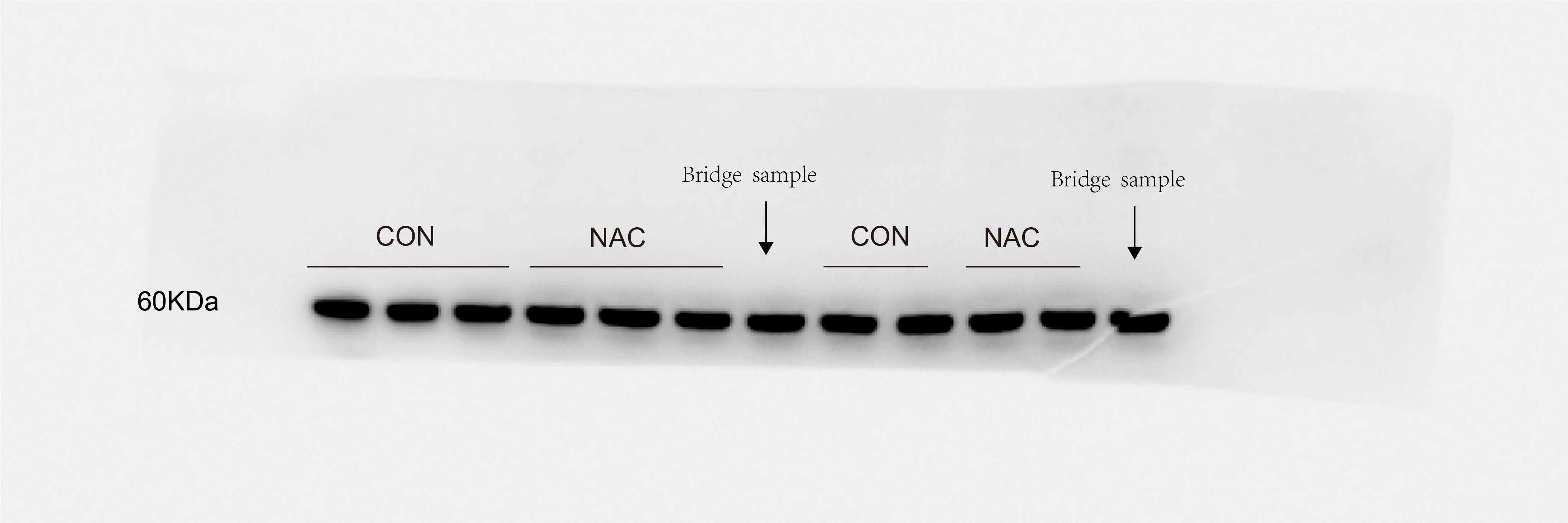


catalase

catalase


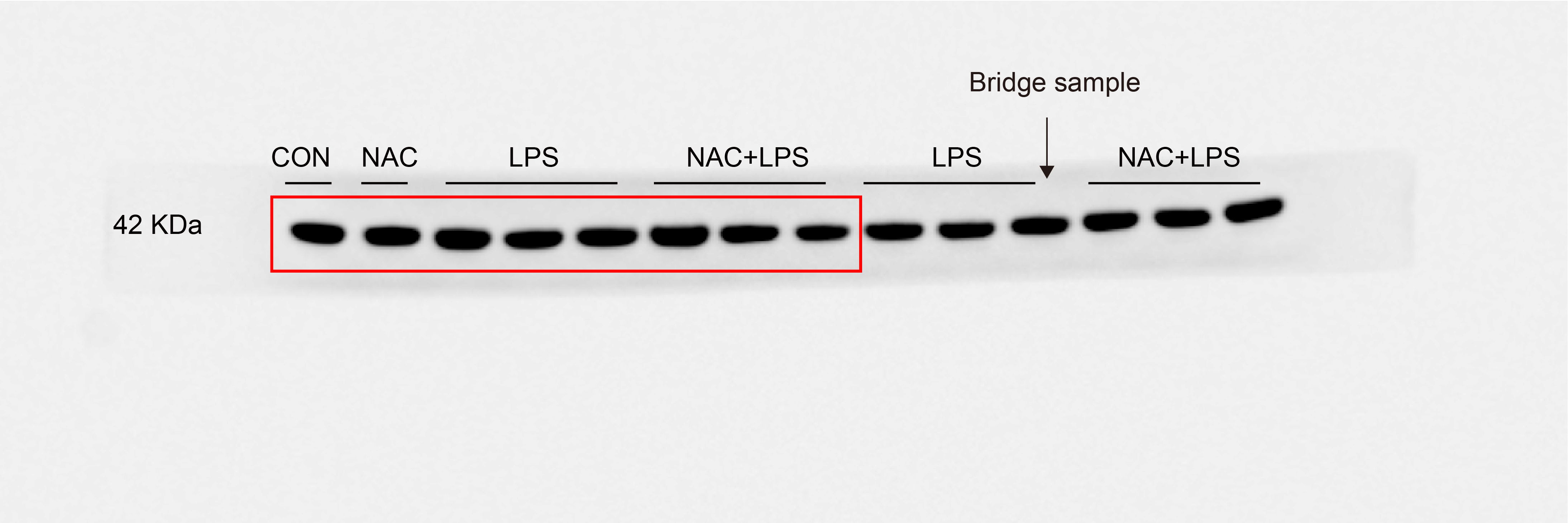

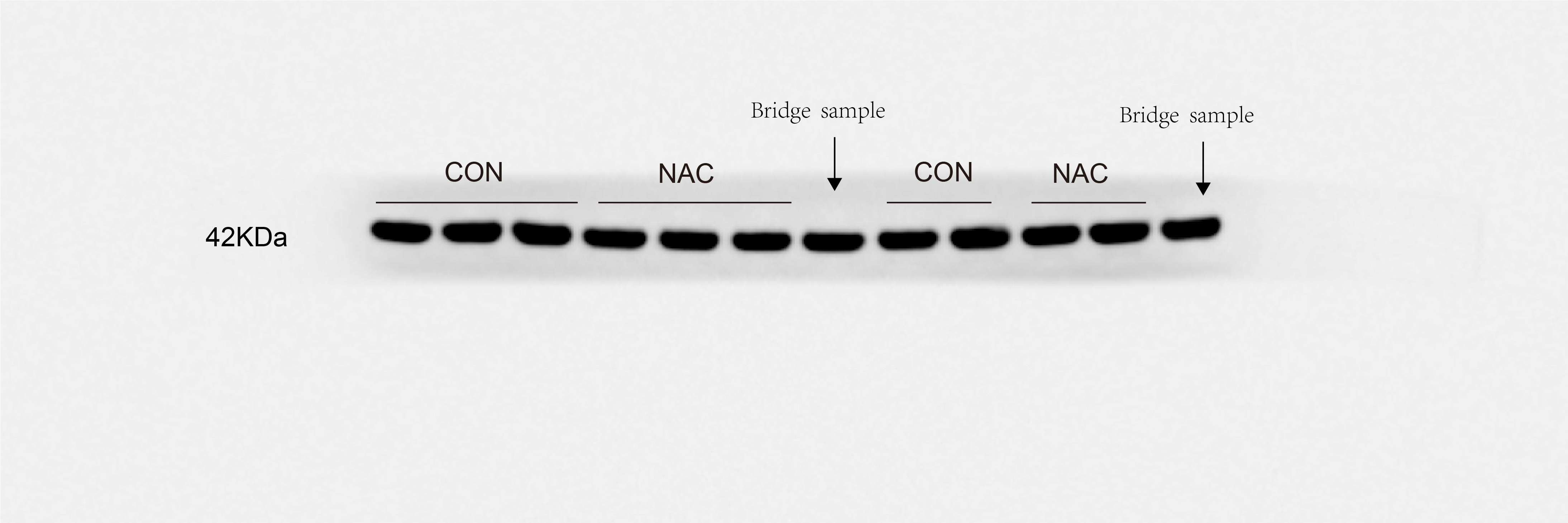


β-actin

β-actin

**Supplementary Figure S6F**

Male

claudin-5

claudin-5


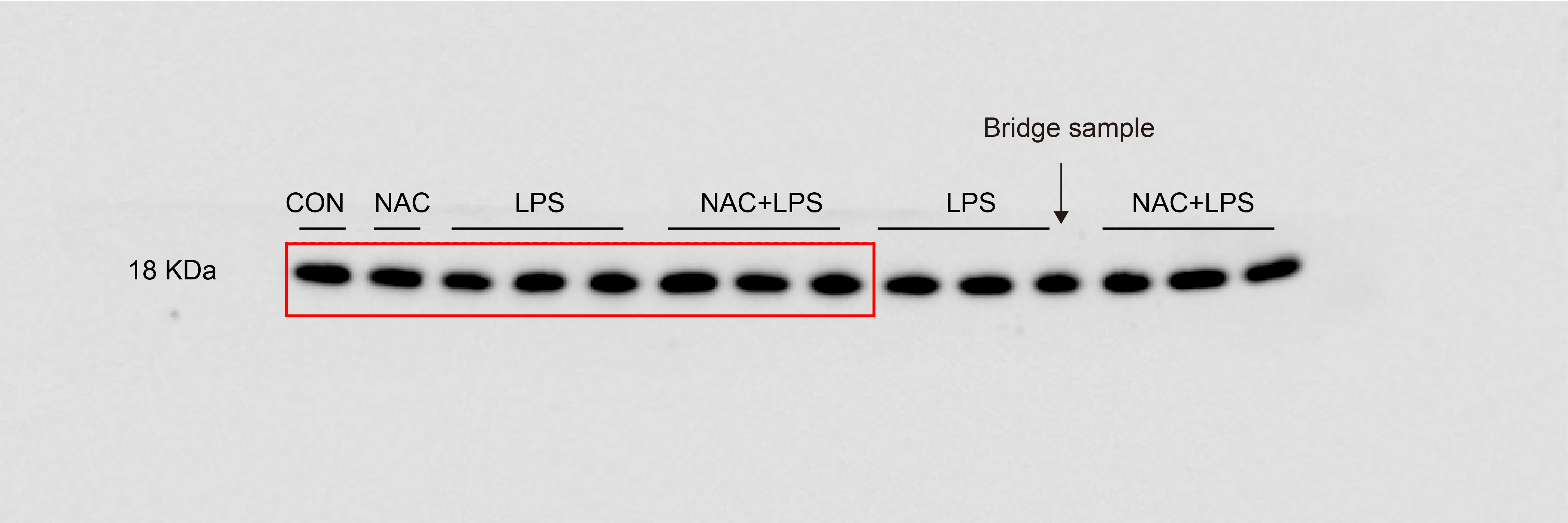

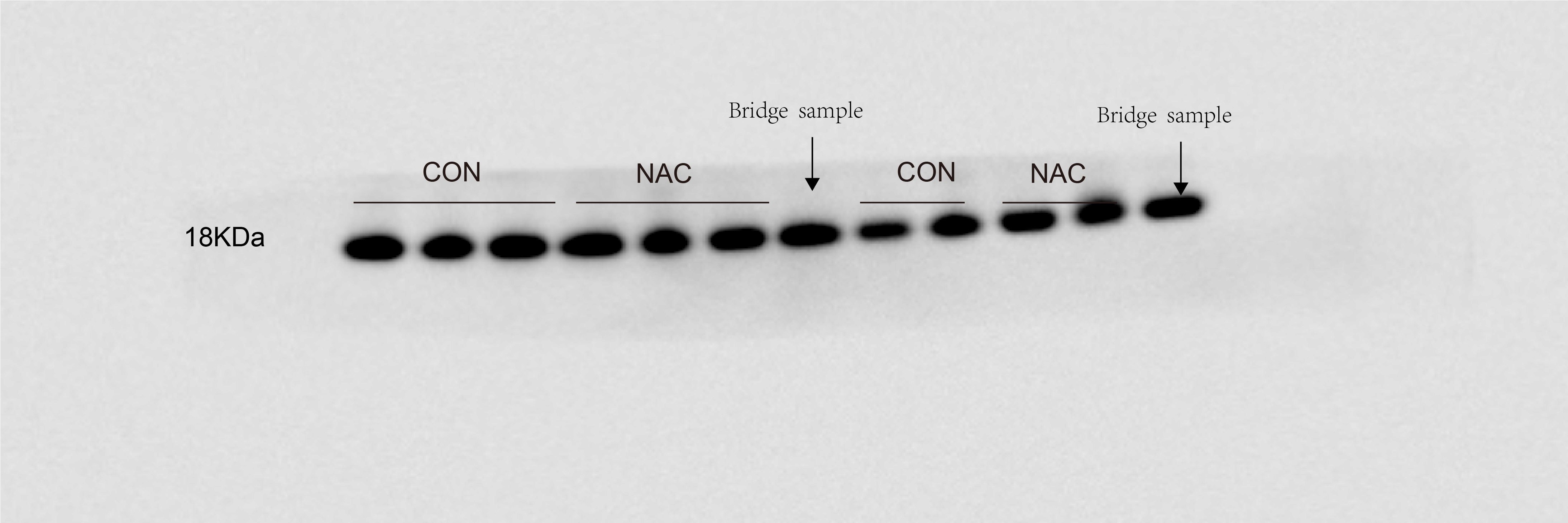


β-actin


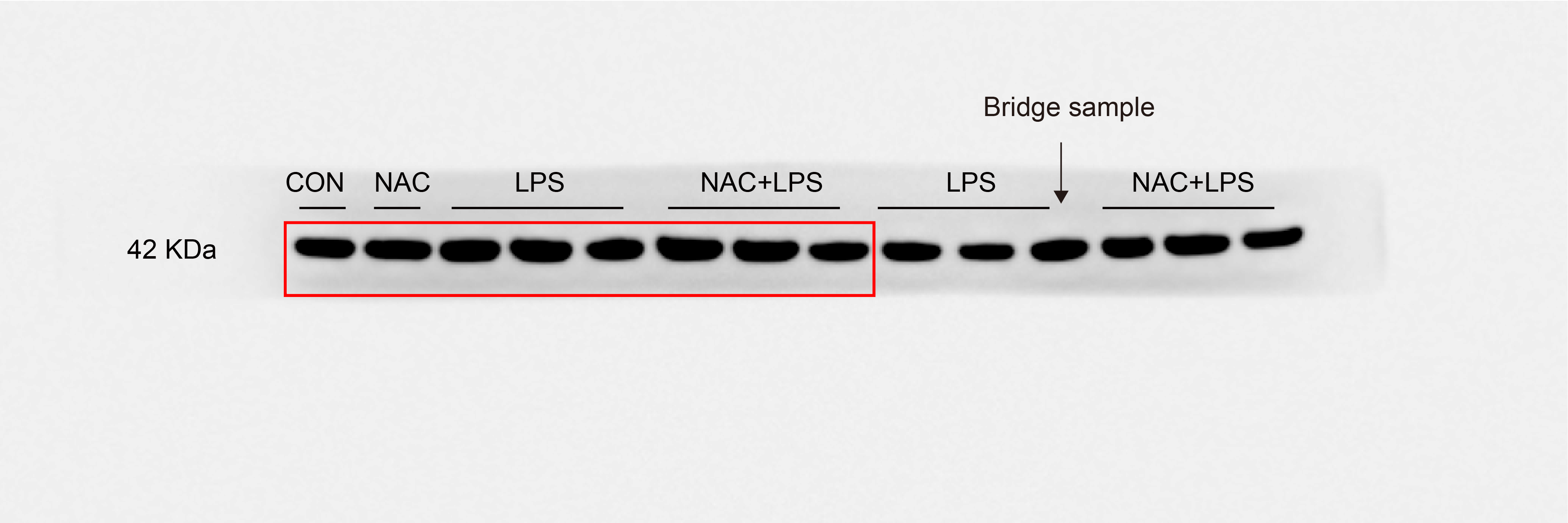

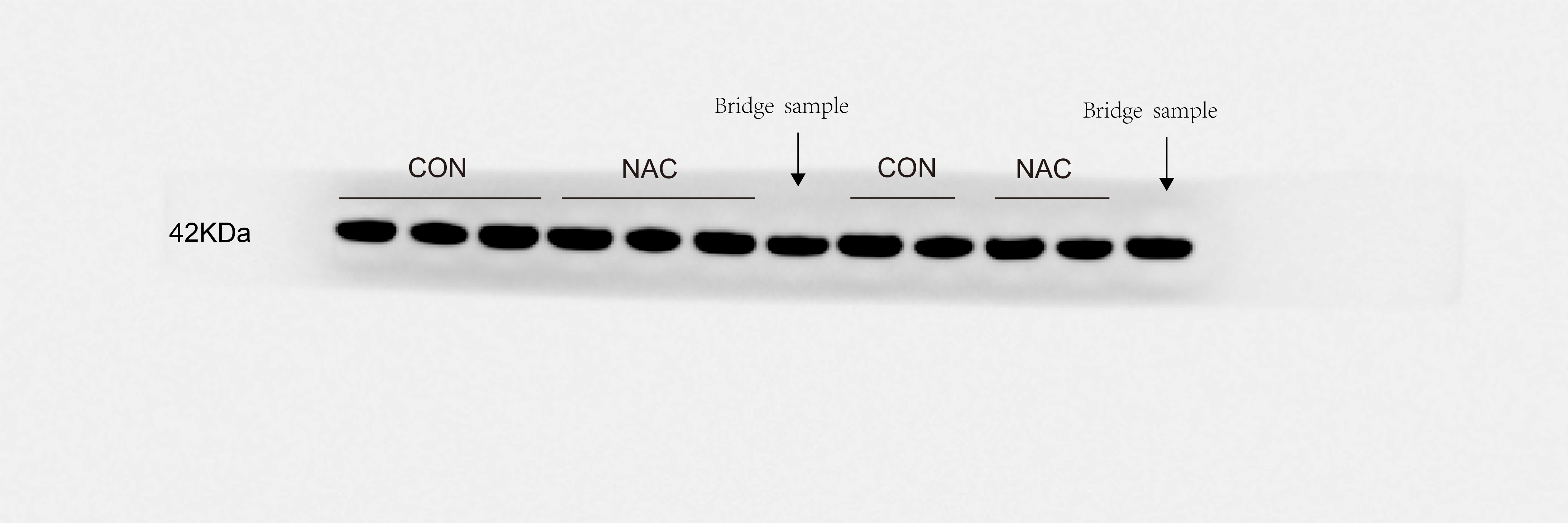


β-actin

Female


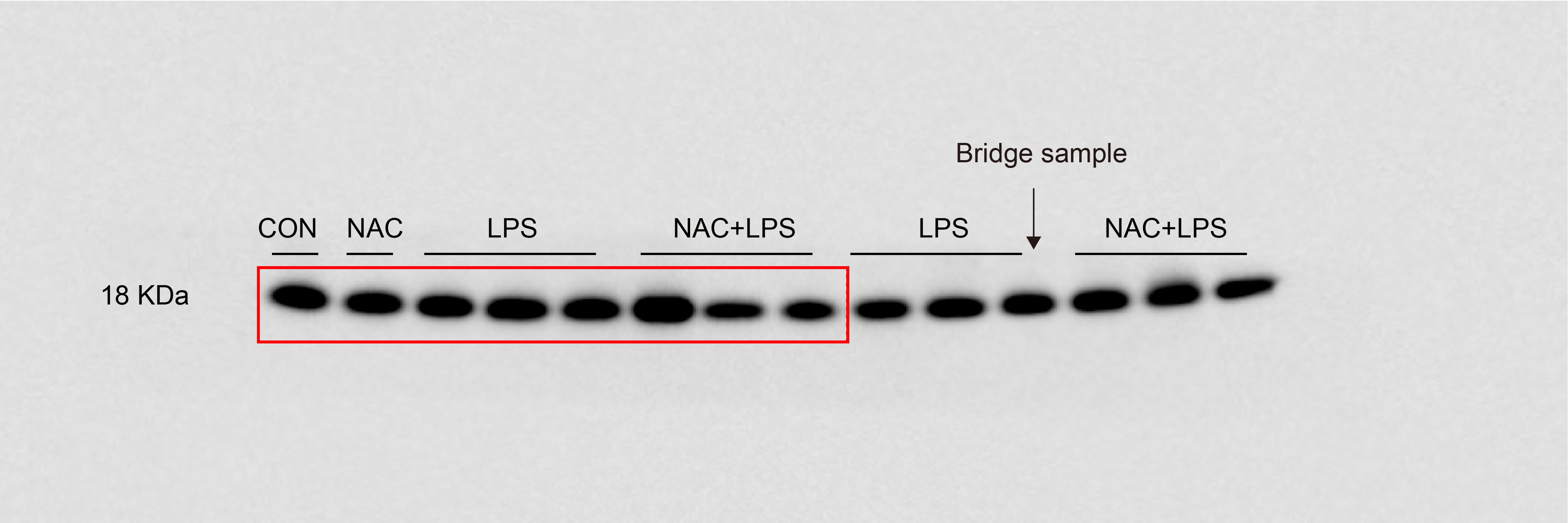

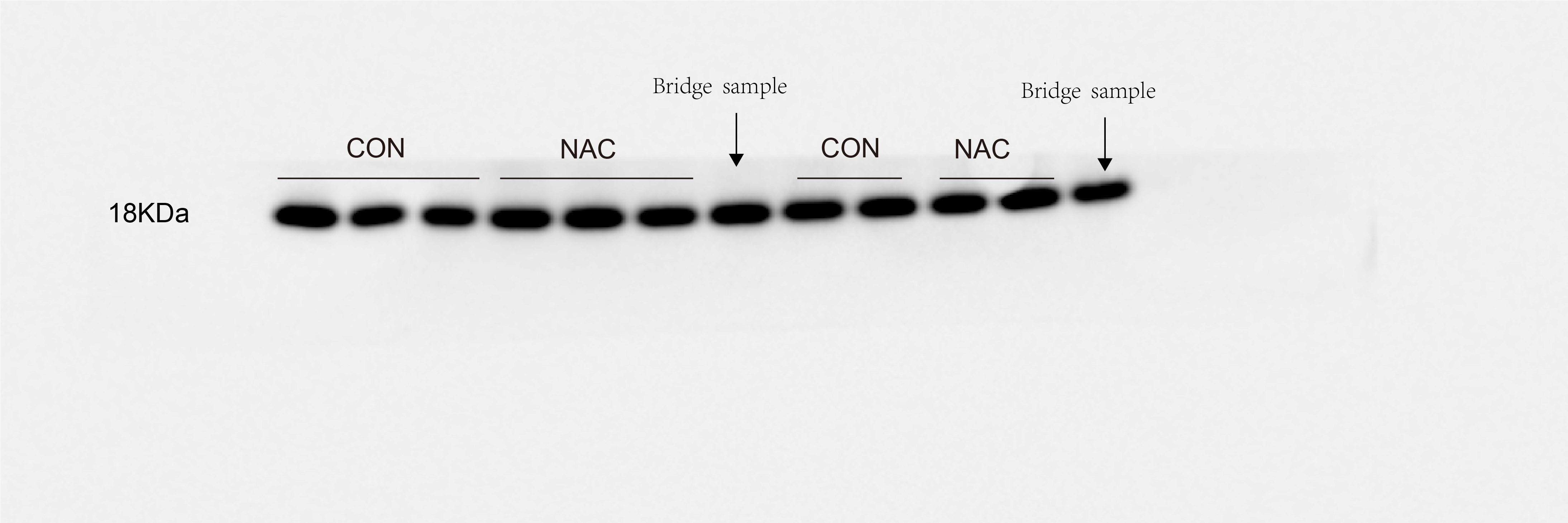


β-actin

claudin-5

β-actin

claudin-5


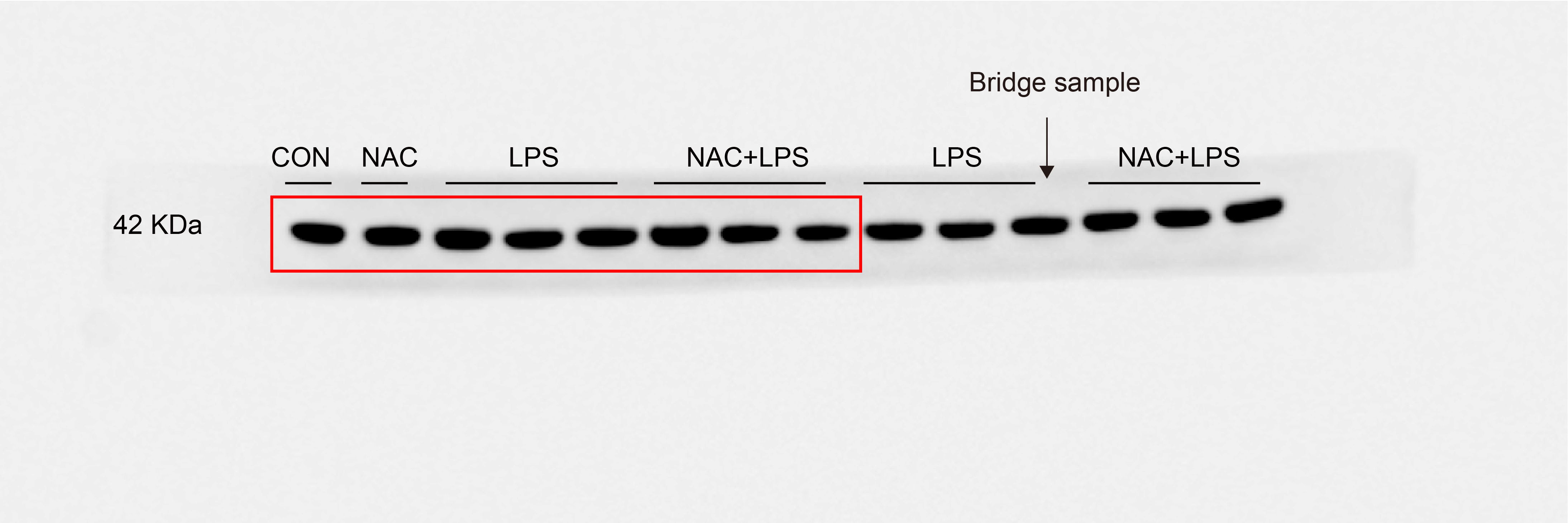

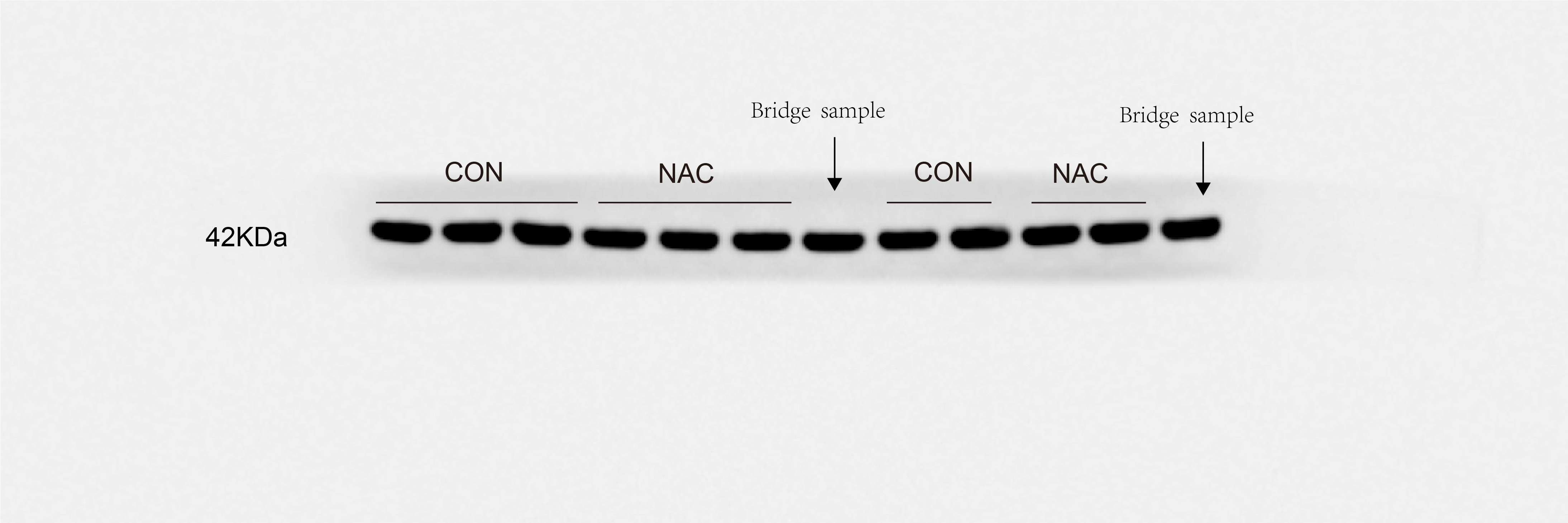

Supplement: Supplementary file 2 — Supplementary Material 2. [file 12974_2026_3942_MOESM2_ESM.docx]
